# Potassium thiocyanate-promoted four-component alkoxysulfenylation of styrenes with imidazo[1,2-*a*]pyridines and alcohols

**DOI:** 10.1039/d6ra02944b

**Published:** 2026-07-02

**Authors:** Chun Ling, Yufeng Yang, Jiale Wu, Zhaohua Yan, Tian Chen

**Affiliations:** a College of Chemistry and Chemical Engineering, Nanchang University Nanchang 330031 Jiangxi Province China yanzh@ncu.edu.cn; b Zhejiang Charioteer Pharmaceutical Co., Ltd Xianju 317321 Zhejiang Province China chentian@charioteer.cn

## Abstract

A green four-component alkoxysulfenylation of styrenes with imidazo[1,2-*a*]pyridines, potassium thiocyanate and alcohols for the synthesis of multifunctionalized imidazo[1,2-*a*]pyridines was developed. In this protocol, inexpensive inorganic salts potassium thiocyanate and potassium persulfate were respectively used as a sulfur source and a radical initiator. A series of C-3 thiolated imidazo[1,2-*a*]pyridines with alkoxy and aryl groups on side chains were efficiently synthesized.

## Abstract 2

A green four-component alkoxysulfenylation of styrenes with imidazo[1,2-*a*]pyridines, potassium thiocyanate and alcohols for the synthesis of multifunctionalized imidazo[1,2-*a*]pyridines was developed.

## Graphical abstract

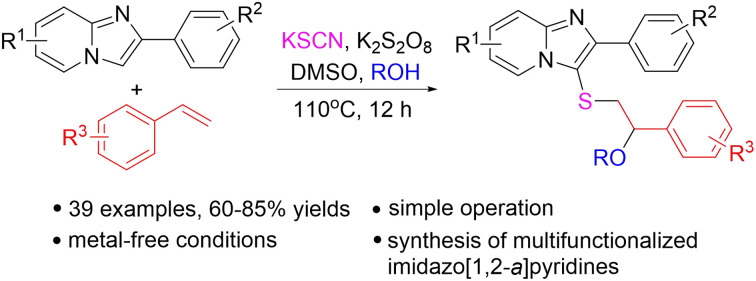

## Introduction

Organosulfur compounds play crucial roles in organic chemistry and they have been widely used in the fields of drug discovery, chiral catalysis, and materials science.^1^ Over the past decade, β-alkoxy sulfides have been attracting increasing attention from chemists. They are not only versatile building blocks in organic synthesis, but also present in many bioactive molecules.^1*b*,2^ Many efficient approaches have been developed for the synthesis of β-alkoxy sulfides. Among them, electrophilic, radical or electrochemical alkene alkoxysulfenylation has proven to be the most attractive and efficient strategy. Sulfonyl hydrazides,^3^ aryl sulfinic acids,^4^ sodium sulfinate,^5^*N*-arylthioimide,^6^ thiophenol,^7^ disulfides,^8^ and sulfoxide^9^ have been successfully employed as sulfur sources in alkoxysulfenylation reactions. Despite these advances, some sulfenylation reagents still suffer from unpleasant odors, and some require prior synthesis before use. Therefore, the development of easily available, inexpensive and efficient sulfenylation reagents for alkoxysulfenylation of alkenes is still highly desirable.

On the other hand, as a type of fused *N*-heterocycle with unique chemical structure, imidazo[1,2-*a*]pyridines exhibit numerous biological activities including anticancer, antifungal, anti-inflammatory, antimicrobial, antiprotozoal, antipyretic, and antiapoptotic properties.^10^ Several imidazo[1,2-*a*]pyridine based drugs, like zolpidem (Sedative and hypnotic agent), alpidem, necopidem, saripidem (anxiolytic agent), zolimidine (treatment of pepticulcer), and olprinone (treatment of acute heart failure) have already been in clinical use.^11^ Furthermore, imidazo[1,2-*a*]pyridines can also serve as molecular sensors and ligands for metallic ions.^12^ In view of their important application value in various fields, the synthesis and structural modification of imidazo[1,2-*a*]pyridines has gained considerable attention from medicinal and synthetic organic chemists over the past decade. Among these, C-3 functionalization of imidazo[1,2-*a*]pyridines has especially become a research hotspot.^13^

**Scheme 1:**
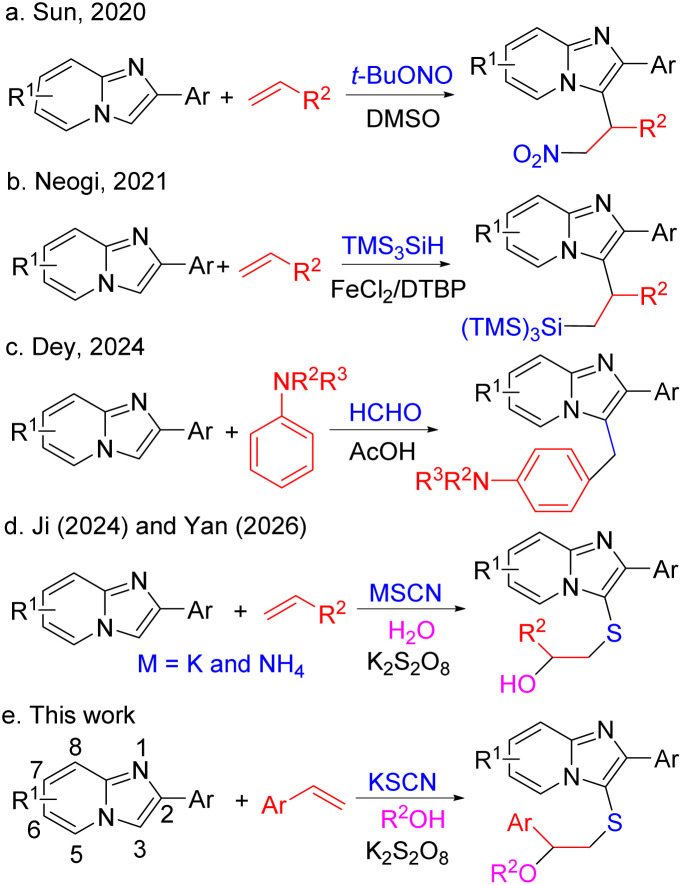
Several multicomponent reactions of imidazo[1,2-*a*]pyridines.

Multicomponent reaction has emerged as a straightforward and efficient strategy for multifunctionalization of candidate compounds. Indeed, in order to synthesize diversely functionalized imidazo[1,2-*a*]pyridines in a single step, several research teams have pioneered their multicomponent reactions in recent years. For example, in 2020, Sun and co-authors described a three-component heteroarylation-nitration of alkenes with imidazo[1,2-*a*]pyridines and *tert*-butylnitrite (Scheme 1b).^14^ In 2021, Neogi's group published a radical three-component carbosilylation reaction of alkenes with imidazopyridines and silanes (Scheme 1c).^15^ In 2024, Dey published an abnormal Mannich-type reaction of imidazo[1,2-*a*]pyridines with anilines and formaldehyde.^16^ Very recently, Ji and Yan respectively reported the four-component hydroxythiolations of styrenes with imidazo[1,2-*a*]pyridines, thiocyanate salt and water.^17^ These novel strategies enabled the synthesis of imidazo[1,2-*a*]pyridines bearing diversified functional groups at C3 position. On the basis of our previous research results,^17*b*^ herein we herein report the potassium thiocyanate-promoted four-component alkoxysulfenylation reaction of styrenes with imidazo[1,2-*a*]pyridines and alcohols. In our protocol, inexpensive, stable and less toxic inorganic salts KSCN and potassium persulfate (K_2_S_2_O_8_) were respectively used as a sulfur source and a free radical initiator.^18^

## Results and discussion

We initiated our optimization studies by selecting the reaction of 2-phenylimidazo[1,2-*a*]pyridine 1a (0.2 mmol) with styrene 2a (0.4 mmol) as a model system and the results were summarized in Table 1. Potassium thiocyanate (KSCN, 0.4 mmol) and the oxidizing agent sodium persulfate (Na_2_S_2_O_8_, 0.6 mmol) can promote the alkoxysulfenylation of styrene to proceed well in a mixed solvent of DMSO (2.0 mL) and methanol (1.0 mL. Acting as methoxy group source and co-solvent) and the desired product β-methoxy sulfide 3a was obtained in 52% yield at 90 °C for 12 hours under N_2_ atmosphere (Table 1, entry 1). Other oxidants, such as hydrogen peroxide, di-*tert*-butyl peroxide (DTBP), *tert*-butyl hydroperoxide (TBHP), azobisisobutyronitrile (AIBN), *m*-chloroperoxybenzoic acid (*m*-CPBA) and K_2_S_2_O_8_ were then screened. Among these, the former five oxidants did not work in this transformation (Table 1, entries 2–6), while K_2_S_2_O_8_ exhibited better result than Na_2_S_2_O_8_ giving 3a in 73% yield (Table 1, entry 7). Additionally, the methoxysulfenylation reaction did not occur in the absence of an oxidant (Table 1, entry 8). Subsequently, various solvents, including *N*,*N*-dimethylformamide (DMF), 1,4-dioxane, acetonitrile (MeCN), tetrahydrofuran (THF), chlorobenzene, toluene and 1,2-dichloroethane were further tested (Table 1, entries 9–15). Among the tested solvents, DMSO was proven to be the best solvent of choice, and this might result from its high polarity facilitating the dissolution of the two inorganic salts KSCN and K_2_S_2_O_8_ in the reaction mixture. The screening results of the reaction temperature indicated that 110 °C can provide the best yield (Table 1, entries 16–18). Furthermore, the use of dry DMSO gave a little bit higher yield of 82% (Table 1, entry 19). Our results disclosed that the trace moisture in conventional DMSO solvent can lead to the formation of by-product β-hydroxy sulfide. Finally NH_4_SCN was used instead of KSCN and only interior yield was obtained (Table 1, entry 20). In summary, the optimum reaction conditions are as follows: 1a (0.2 mmol), 2a (0.4 mmol), KSCN (0.4 mmol), K_2_S_2_O_8_ (0.6 mmol), in dry DMSO/MeOH (v/v, 2/1) at 110 °C for 12 h.

**Table 1:** Optimization of reaction conditions^a^

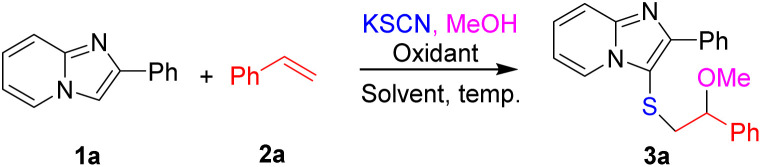				
Entry	Oxidant (equiv.)	Solvent (mL)	Temp.(°C)	Yield (%)^b^
1	Na_2_S_2_O_8_	DMSO/MeOH	90	52
2	H_2_O_2_	DMSO/MeOH	90	Trace
3	DTBP	DMSO/MeOH	90	NR
4	TBHP	DMSO/MeOH	90	NR
5	AIBN	DMSO/MeOH	90	NR
6	*m*-CPBA	DMSO/MeOH	90	NR
7	K_2_S_2_O_8_	DMSO/MeOH	90	73
8	None	DMSO/MeOH	90	Trace
9	K_2_S_2_O_8_	DMF/MeOH	90	63
10	K_2_S_2_O_8_	1,4-Dioane/MeOH	90	46
11	K_2_S_2_O_8_	MeCN/MeOH	90	70
12	K_2_S_2_O_8_	THF/MeOH	90	70
13	K_2_S_2_O_8_	PhCl/MeOH	90	69
14	K_2_S_2_O_8_	PhMe/MeOH	90	65
15	K_2_S_2_O_8_	(ClCH_2_)_2_/MeOH	90	68
16	K_2_S_2_O_8_	DMSO/MeOH	100	76
17	K_2_S_2_O_8_	DMSO/MeOH	110	79
18	K_2_S_2_O_8_	DMSO/MeOH	120	73
**19**	**K**_**2**_ **S**_**2**_ **O**_**8**_	**dry DMSO/MeOH**	**110**	**83**
20^c^	K_2_S_2_O_8_	dry DMSO/MeOH	110	56

a Reaction conditions: 1a (0.4 mmol, 2 equiv.), 2a (0.2 mmol), oxidant (0.6 mmol, 3 equiv.), KSCN (0.4 mmol, 2 equiv.), solvent (2.0 mL) and MeOH (1.0 mL) in a sealed tube, stirred at 80–120 °C under nitrogen for 12 hours.

b Isolated yields.

c Using NH_4_SCN instead of KSCN.

**Scheme 2:**
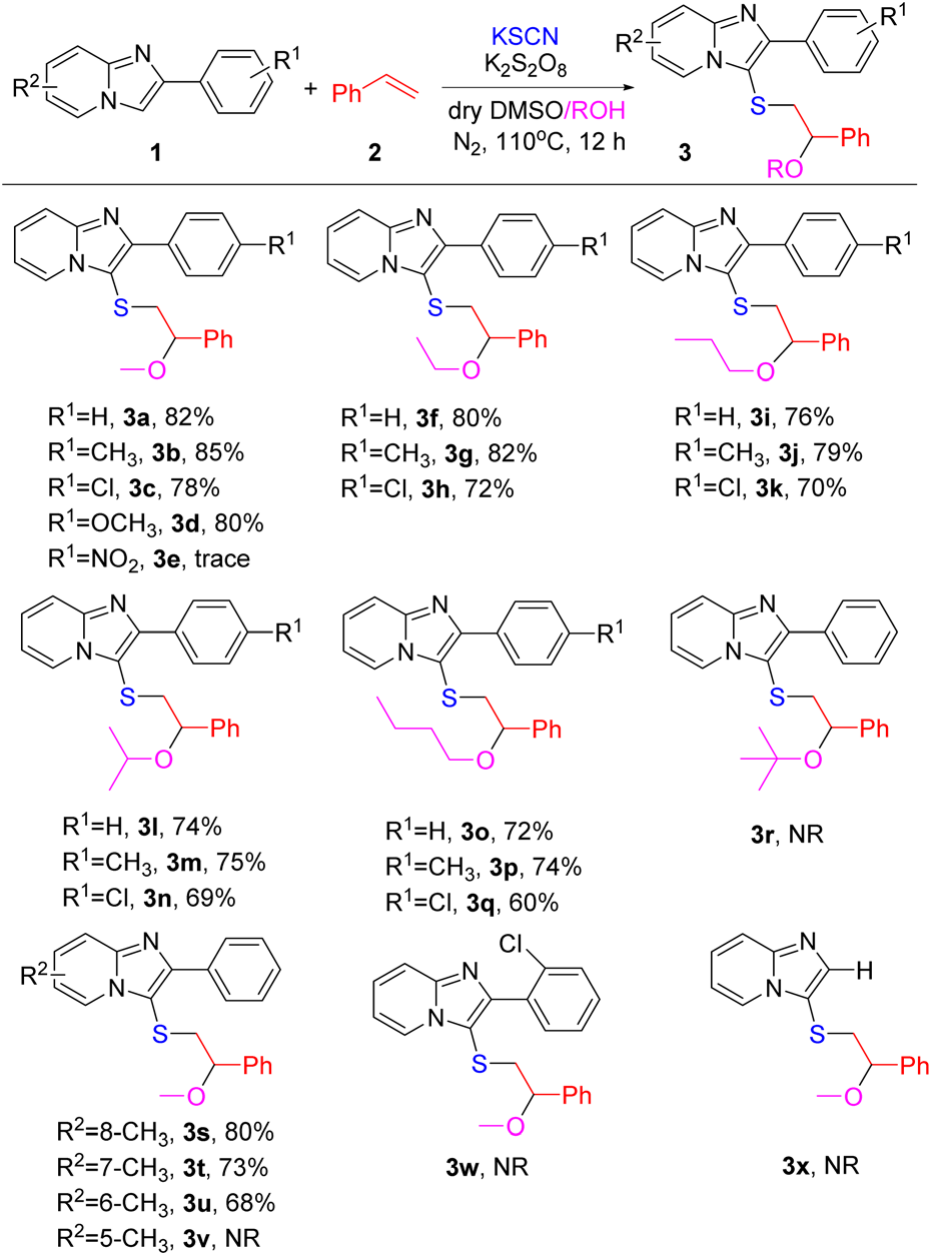
Substrate scope. Reaction conditions: 1 (0.4 mmol, 2 equiv.), 2 (0.2 mmol), K_2_S_2_O_8_ (0.6 mmol, 3 equiv.), KSCN (0.4 mmol, 2 equiv.), dry DMSO (2.0 mL) and ROH (1.0 mL) in a sealed tube, stirred at 110 °C under nitrogen for 12 hours. Isolated yields were given.

With the optimized reaction conditions in hand, the scope of imidazo[1,2-*a*]pyridines and alcohols was investigated to assess the universality of this transformation and the results were illustrated in Scheme 2. When the R^1^ groups at the *para* position of the phenyl ring were electron-donating or weak electron-withdrawing groups (H, CH_3_, Cl, OCH_3_), the corresponding products could be obtained in good yields (3a–3d). When R^1^ was a strong electron-withdrawing group (NO_2_), only trace amount of product 3e was detected. This might be attributed to the fact that the strong electron-withdrawing group can inhibit the formation of C-2 radical of imidazo[1,2-*a*]pyridines. Besides methanol, ethanol, *n*-propanol, isopropanol and *n*-butanol are all applicable to this alkoxysulfenylation reaction (3f–3q). However *tert*-butanol was not a suitable partner for this transformation, probably resulting from its high steric hindrance (3r). The electron-rich substituents at the 6, 7, or 8 position of the pyridine ring have no impact on the reaction (3s–3u). Substrates with a substituent at the 5 position of the pyridine ring and at the *ortho* position of the benzene ring inhibited the reactions (3v, 3w). Likewise, imidazo[1,2-*a*]pyridine is also not a suitable substrate (3x).

**Scheme 3:**
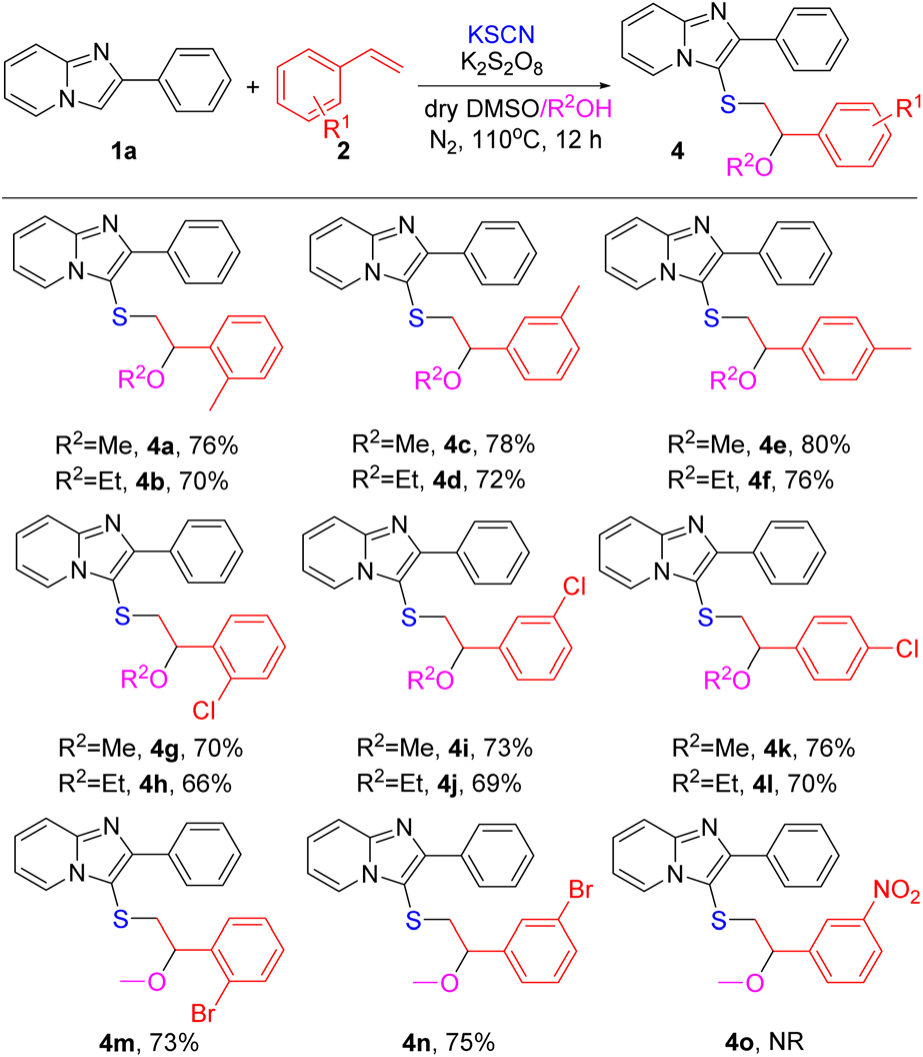
Substrate scope. Reaction conditions: 1a (0.4 mmol, 2 equiv.), 2 (0.2 mmol), K_2_S_2_O_8_ (0.6 mmol, 3 equiv.), KSCN (0.4 mmol, 2 equiv.), dry DMSO (2.0 mL) and R^2^OH (1.0 mL) in a sealed tube, stirred at 110 °C under nitrogen for 12 hours. Isolated yields were given.

Next, a variety of styrene substrates were further evaluated under the optimal reaction conditions in order to broaden the substrate scope. As depicted in Scheme 3, styrene substrates with weak electron-withdrawing and electron-donating groups on the benzene ring are all tolerated under this alkoxysulfenylation reaction conditions affording the corresponding β-alkoxy sulfides in 66–81% yields (4a–4y). However, it is a pity that styrene with a strong electron-withdrawing group on the benzene ring and aliphatic olefins are still not compatible with this reaction (4z).

**Scheme 4:**
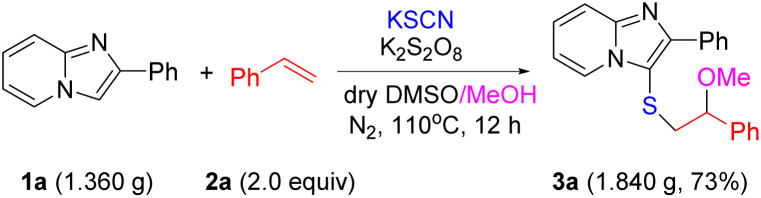
Gram-scale synthesis.

To validate the applicability of the four-component alkoxysulfenylation reaction, we conducted gram-scale experiment using model substrates 1a and 2a under the optimized reaction conditions (Scheme 4). The reaction scale was increased from 0.2 mmol to 7.0 mmol. Upon completion of the reaction, the target product 3a was obtained in 73% yield (1.840 g). This result further demonstrated that this reaction can be applied in organic synthesis.

**Scheme 5:**
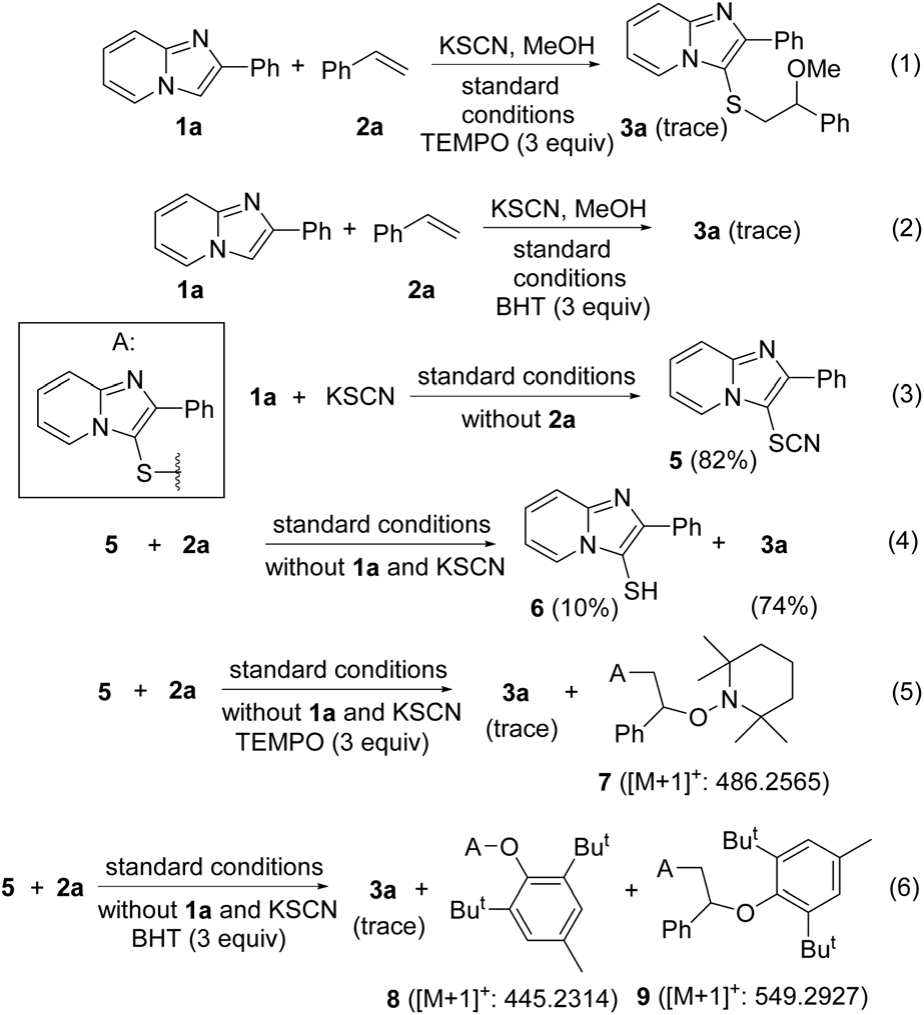
Control experiments.

To clarify the mechanism of this cascade reaction, we conducted several control experiments (Scheme 5). Under standard reaction conditions, the addition of a radical scavenger 2,2,6,6-tetramethylpiperidine-1-oxyl (TEMPO) or antioxidant 2,6-Di-*tert*-butyl-p-cresol (BHT) (Scheme 5 and eqn (1) and (2)) led to the formation of trace amount of target product 3a. This suggests the reaction proceeds *via* a radical pathway. Treating 1a with KSCN without 2a under standard conditions (Scheme 5 and eqn (3)) produced C-3 thiocyanated product 5. This result confirms that 1a can react independently with KSCN and that 5 acts as a crucial precursor for subsequent steps. Subsequently, the reaction of 5 with 2a and MeOH in DMSO (Scheme 5 and eqn (4)) yielded 3a in 74% yield along with the formation of minor amount of thiol product 6. In the presence of TEMPO or BHT (Scheme 5 and eqn (5) and (6)), the reactions of 5 with 2a and MeOH in DMSO were all inhibited and 7, 8 and 9 were all detected by GC-MS.

**Scheme 6:**
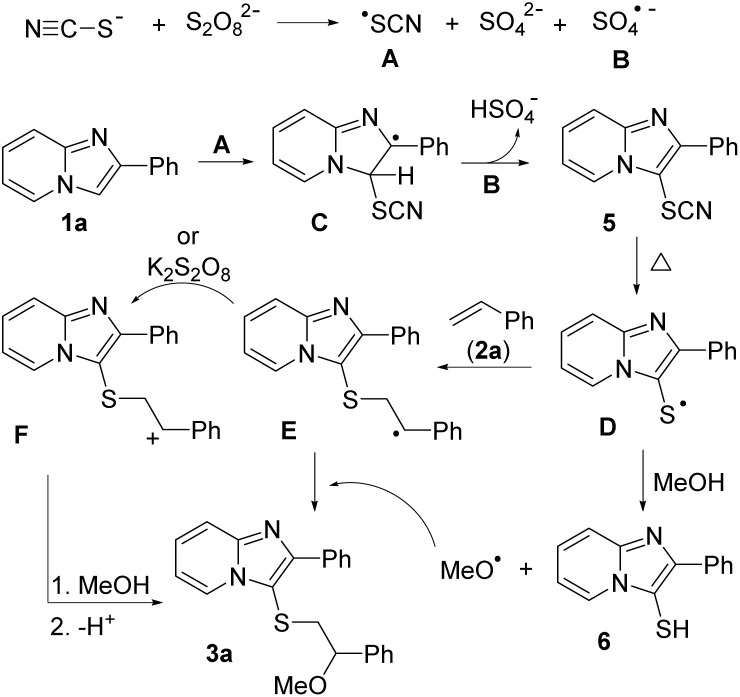
Proposed mechanism.

Based on the above experimental results and literature reports,^17^ a plausible mechanism is proposed (Scheme 6). First, the thiocyanate anion was oxidized by the persulfate anion yielding a radical A and the sulfate radical B. The unsaturated C3 site of 2-phenylimidazo[1,2-*a*]pyridine is attacked by the electrophilic radical A yielding a radical intermediate C. C subsequently loses a H radical and rearomatizes to give the stable key intermediate 5. On heating 5 undergoes homolytic cleavage to generate sulfur radical D. Subsequently, D attacks the double bond of styrene to form radical E. Furthermore, D reacts with methanol to generate a methoxy radical and 6. The methoxy radical then combines with E to produce the target product 3a. Alternatively, E can be further oxidized to cationic F, which also undergoes nucleophilic attack by methanol to yield the same target product 3a.

## Conclusions

In summary, a transition-metal-free, four-component radical cascade alkoxysulfenylation of styrenes with imidazo[1,2-*a*]pyridines, KSCN and alcohols was developed. In this strategy, inexpensive and less toxic inorganic salt KSCN and K_2_S_2_O_8_ were respectively used as a sulfur source and a radical initiator. A series of imidazo[1,2-*a*]pyridine derivatives bearing phenyl, alkoxy and thioether groups at C-3 site were efficiently synthesized in a single step, thus providing a broader range of target molecules for the drug screening of imidazopyridines. The preliminary biological activity assays demonstrated that the products (3 and 4) are potentially excellent lead compounds for Aurora A kinase inhibition.^19^

## Author contributions

CL, YY and JW performed the experiments and analysed the results. TC ran HRMS spectra of all products. ZY and TC supervised the research and analysed the results. ZY conceived the project. ZY drafted the paper. All authors approved the paper.

## Conflicts of interest

There are no conflicts to declare.

## Supplementary Material

RA-016-D6RA02944B-s001

## Data Availability

Supplementary information (SI): this is to confirm that the synthetic procedure, spectroscopic data, H-1/C-13 spectra of all the synthesized products, and biological assay data of product 3a and 3b in our manuscript. See DOI: https://doi.org/10.1039/d6ra02944b.
