# Supplementary material for: Potassium thiocyanate-promoted four-component alkoxysulfenylation of styrenes with imidazo[1,2-a]pyridines and alcohols
Source: RSC Adv. 2026 Jul 2;16(34):33273–7. doi: 10.1039/d6ra02944b (PMC13326684; doi:10.1039/d6ra02944b)

Supplementary Information (SI) for RSC Advances.
This journal is © The Royal Society of Chemistry 2026

Supporting information

Potassium thiocyanate-promoted four-component alkoxysulfenylation of styrenes with imidazo[1,2-*a*]pyridines and alcohols

Chun Ling[a], Yufeng Yang[a], Jiale Wu[a], Zhaohua Yan*[a], Tian Chen*[b]

([a] *College of Chemistry and Chemical Engineering, Nanchang University, Nanchang, 330031, Jiangxi Province, China*)

([b] *Zhejiang Charioteer Pharmaceutical Co., Ltd. Xianju, 317321, Zhejiang Province, China*)

Email: yanzh@ncu.edu.cn (Zhaohua Yan)

CONTENTS

1. General information (page 2)
2. General procedure for the four-component alkoxysulfenylation of styrenes (page 2)
3. Characterization data of products (page 2-12)
4. Bioactivity assay results of **3a** and **3b** (page 13)
5. Microplate reader assay data of **3a** and **3b** (page 14-15)
6. Fitting data of **3a** (page 16)
7. Fitting data of **3b** (page 17-18)
8. ^{1}H and ^{13}C NMR spectra of all the products (page 19-52)

1. General information

Unless otherwise stated, all reagents were commercially available and used without further purification. All reactions were performed in a double-necked flask. 400 MHz ^{1}H NMR and 101 MHz ^{13}C NMR spectra were measured on Agilent spectrometer, using $CDCl_3$, d_6-DMSO as the solvent with tetramethylsilane (TMS) as the internal standard at room temperature and chemical shifts are expressed in δ ppm. HRMS spectra were recorded by Agilent 6545. Flash column chromatographic purification of products was accomplished by forced-flow chromatography on silica gel (300−400 mesh) using petroleum ether/ethyl acetate as eluent.

2. **The general procedure for the four-component alkoxysulfenylation of styrenes with imidazo[1,2-*a*]pyridines, KSCN and alcohols**

All the imidazo[1,2-*a*]pyridine substrates were synthesized according to the following procedure (please see reference: K. Godugu, C. G. R. Nallagondu, "Solvent and catalyst-free synthesis of imidazo[1,2-a]pyridines by grindstone chemistry". *J. Heterocyclic Chem.* **2021**, *58*, 250-259).

The mixture of 2-phenylimidazo[1,2-a]pyridine **1a** (0.2 mmol, 1.0 equiv), styrene **2a** (0.4 mmol, 2.0 equiv), KSCN (0.4 mmol, 2.0 equiv), and $K_2S_2O_8$ (0.6 mmol, 3.0 equiv) in a mixed solvent of dry-DMSO/ROH (3 ml. v/v 2:1) was stirred in an atmosphere of N_2 at 110°C for 12 hours. After the reaction was completed, the reaction mixture was cooled to room temperature, and then water was added. The mixture was then extracted with dichloromethane. The organic phase was collected. It was then dried with anhydrous Na_2SO_4 and rotary evaporated to give a crude product. The crude product was further purified through flash column chromatography using a mixture of petroleum ether and ethyl acetate as eluent (PE/EA = 100/1~20/1, v/v).

3. Characterization data of all the products

3-((2-methoxy-2-phenyl)ethylthio)-2-phenylimidazo[1,2-*a*]pyridine (3a)

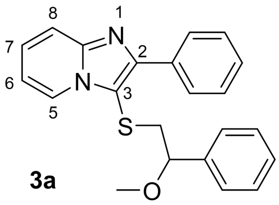

3a

Isolated yield 82%. Pale yellow oil. ^{1}H NMR (600 MHz, $CDCl_3$) δ 8.29 (d, *J* = 7.8 Hz, 1H), 8.22 (d, *J* = 7.2 Hz, 2H), 7.57 (m, 1H), 7.40 (t, *J* = 7.8 Hz, 2H), 7.32 (t, *J* = 7.4 Hz, 2H), 7.21-7.17 (m, 3H), 7.01 (dd, *J* = 7.8, 1.8 Hz, 2H), 6.80 (dd, *J* = 7.2, 2.0 Hz, 1H), 3.91 (dd, *J* = 8.6, 4.1 Hz, 1H), 2.93 (s, 3H), 2.85 (dd, *J* = 13.5, 8.6 Hz, 1H), 2.79 (dd, *J* = 13.6, 4.1 Hz, 1H); ^{13}C NMR (151 MHz, $CDCl_3$) δ 150.39, 146.09, 139.74, 133.40, 132.59, 128.57, 128.54, 128.46, 128.37, 128.21, 126.57, 125.11, 116.30, 114.08, 110.52, 82.14, 56.65, 43.00. HRMS (ESI-TOF) m/z Calcd for $C_{22}H_{21}N_2OS$ [M+H]$^+$: 361.1375, found: 361.1370.

3-((2-methoxy-2-phenylethyl)thio)-2-(4-methylphenyl)imidazo[1,2-*a*]pyridine (**3b**)

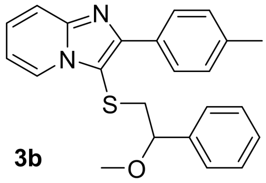

3b

Isolated yield 85%. Pale yellow oil. ^{1}H NMR (600 MHz, $CDCl_3$) δ 8.38 (d, *J* = 6.8 Hz, 1H), 8.15 (d, *J* = 8.04 Hz, 2H), 7.57 (d, *J* = 9.0 Hz, 1H), 7.21-7.14 (m, 6H. The peak of $CDCl_3$ overlap with it), 7.01 (d, *J* = 6.4 Hz, 2H), 6.81 (t, *J* = 6.8 Hz, 1H), 3.91 (dd, *J* = 8.7, 4.0 Hz, 1H), 2.94 (s, 3H), 2.86 (dd, *J* = 13.6, 8.8 Hz, 1H), 2.78 (dd, *J* = 13.5, 4.1 Hz, 1H), 2.34 (s, 3H); ^{13}C NMR (151 MHz, $CDCl_3$) δ 149.77, 146.43, 140.00, 138.11, 130.99, 129.09, 128.49, 128.30, 128.09, 126.60, 125.83, 124.64, 117.38, 112.40, 109.54, 82.20, 56.63, 42.85, 21.34. HRMS (ESI-TOF) m/z Calcd for $C_{23}H_{23}N_2OS$ $[M+H]^+$: 375.1531, found: 375.1546.

2-(4-chlorophenyl)-3-((2-methoxy-2-phenyl)ethylthio)imidazo[1,2-*a*]pyridine (**3c**)

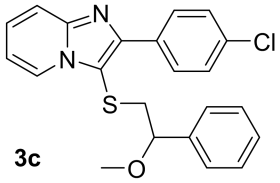

3c

Isolated yield 78%. Pale yellow oil. ^{1}H NMR (600 MHz, $CDCl_3$) δ 8.37 (d, *J* = 6.8 Hz, 1H), 8.23 (d, *J* = 8.5 Hz, 2H), 7.56 (d, *J* = 8.9 Hz, 1H), 7.36 (d, *J* = 8.5 Hz, 2H), 7.23-7.16 (m, 4H. The peak of $CDCl_3$ overlap with it), 7.01 (d, *J* = 6.4 Hz, 2H), 6.82 (t, *J* = 6.7 Hz, 1H), 3.92 (dd, *J* = 8.5, 4.1 Hz, 1H), 2.96 (s, 3H), 2.87 (dd, *J* = 13.4, 8.6 Hz, 1H), 2.78 (dd, *J* = 13.5, 4.1 Hz, 1H); ^{13}C NMR (151 MHz, $CDCl_3$) δ 148.38, 146.47, 139.83, 134.21, 132.40, 129.64, 128.52, 128.17, 126.55, 126.11, 124.67, 117.52, 112.63, 110.13, 82.23, 56.62, 42.76. HRMS (ESI-TOF) m/z Calcd for $C_{22}H_{20}ClN_2OS$ $[M+H]^+$: 395.0985, found: 395.0984.

2-(4-methoxyphenyl)-3-((2-methoxy-2-phenyl)ethylthio)imidazo[1,2-*a*]pyridine (**3d**)

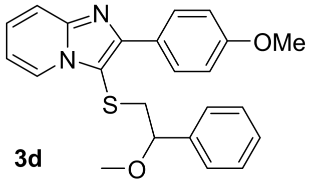

3d

Isolated yield 80%. Pale yellow oil. ^{1}H NMR (600 MHz, $CDCl_3$) δ 8.37 (d, *J* = 6.8 Hz, 1H), 8.22 (d, *J* = 8.9 Hz, 2H), 7.55 (d, *J* = 8.9 Hz, 1H), 7.20-7.15 (m, 4H. The peak of $CDCl_3$ overlap with it), 7.01 (d, *J* = 7.4 Hz, 2H), 6.94-6.92 (m, 2H), 6.79 (d, *J* = 6.7 Hz, 1H), 3.91 (dd, *J* = 8.6, 4.0 Hz, 1H), 3.79 (s, 3H), 2.94 (s, 3H), 2.86 (dd, *J* = 13.6, 8.8 Hz, 1H), 2.78 (dd, *J* = 13.6, 4.1 Hz, 1H); ^{13}C NMR (151 MHz, $CDCl_3$) δ 159.78, 149.56, 146.43, 140.00, 129.70, 128.50, 128.10, 126.60, 126.51, 125.81, 124.59, 117.25, 113.81, 112.34, 108.91, 82.16, 56.65, 55.30, 42.79. HRMS (ESI-TOF) m/z Calcd for $C_{23}H_{23}N_2O_2S$ $[M+H]^+$: 391.1480, found: 391.1472.

2-((2-ethoxy-2-phenylethyl)thio)-2-phenylimidazo[1,2-*a*]pyridine (3f)

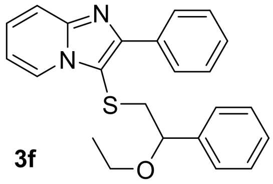

3f

Isolated yield 80%. Pale yellow oil. ^{1}H NMR (600 MHz, $CDCl_3$) δ 8.39 (d, *J* = 6.4 Hz, 1H), 8.24 (d, *J* = 13.8 Hz, 2H), 7.57 (d, *J* = 8.9 Hz, 1H), 7.39 (t, *J* = 7.8 Hz, 2H), 7.30 (t, *J* = 7.4 Hz, 1H), 7.19-7.14 (m, 4H), 7.01 (d, *J* = 6.7 Hz, 2H), 6.80 (t, *J* = 6.7 Hz, 1H), 4.01 (dd, *J* = 8.5, 4.1 Hz, 1H), 3.16-3.11 (m, 1H), 2.97-2.92 (m, 1H), 2.88 (dd, *J* = 13.5, 8.6 Hz, 1H), 2.78 (dd, *J* = 13.5, 4.1 Hz, 1H), 0.98 (t, *J* = 7.0 Hz, 3H); ^{13}C NMR (151 MHz, $CDCl_3$) δ 149.53, 146.43, 140.74, 133.91, 128.46, 128.45, 128.33, 128.21, 127.95, 126.49, 125.86, 124.71, 117.49, 112.45, 110.15, 80.51, 64.39, 42.96, 15.10. HRMS (ESI-TOF) m/z Calcd for $C_{23}H_{23}N_2OS$ $[M+H]^+$: 375.1531, found: 375.1534.

3-((2-ethoxy-2-phenylethyl)thio)-2-(4-methylphenyl)imidazo[1,2-*a*]pyridine (3g)

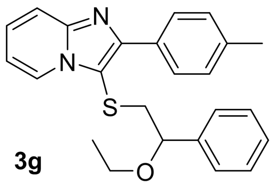

3g

Isolated yield 82%. Pale yellow oil. ^{1}H NMR (600 MHz, $CDCl_3$) δ 8.45 (d, *J* = 6.8 Hz, 1H), 8.22 (d, *J* = 8.0 Hz, 2H), 7.63 (d, *J* = 9.0 Hz, 1H), 7.28-7.21 (m, 6H), 7.09 (d, *J* = 6.8 Hz, 2H), 6.86 (t, *J* = 6.8 Hz, 1H), 4.10 (dd, *J* = 8.5, 4.1 Hz, 1H), 3.24-3.19 (m, 1H), 3.07-3.02 (m, 1H), 2.97 (dd, *J* = 13.5, 8.6 Hz, 1H), 2.85 (dd, *J* = 13.5, 5.6 Hz, 1H), 2.41 (s, 3H), 1.06 (t, *J* = 7.0 Hz, 3H); ^{13}C NMR (151 MHz, $CDCl_3$) δ 149.68, 146.39, 140.79, 138.04, 131.06, 129.05, 128.42, 128.32, 127.92, 126.50, 125.74, 124.66, 117.37, 112.32, 109.74, 80.52, 64.38, 42.93, 21.32, 15.09. HRMS (ESI-TOF) m/z Calcd for $C_{24}H_{25}N_2OS$ $[M+H]^+$: 389.1688, found: 389.1693.

2-(4-chlorophenyl)-3-((2-ethoxy-2-phenylethyl)thio)imidazo[1,2-*a*]pyridine (3h)

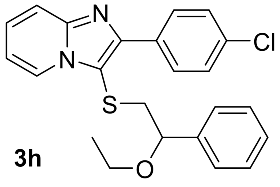

3h

Isolated yield 72%. Pale yellow oil. ^{1}H NMR (600 MHz, $CDCl_3$) δ 8.37 (d, *J* = 6.8 Hz, 1H), 8.21 (d, *J* = 8.5 Hz, 2H), 7.56 (d, *J* = 8.9 Hz, 1H), 7.35 (d, *J* = 8.5 Hz, 2H), 7.21-7.16 (m, 4H), 7.02 (d, *J* = 6.8 Hz, 2H), 6.82 (t, *J* = 6.7 Hz, 1H), 4.05 (dd, *J* = 8.4, 4.2 Hz, 1H), 3.17-3.15 (m, 1H), 3.00-2.97 (m, 1H), 2.90 (dd, *J* = 13.4, 8.5 Hz, 1H), 2.79 (dd, *J* = 13.4, 4.2 Hz, 1H), 0.99 (t, *J* = 7.0 Hz, 3H); ^{13}C NMR (151 MHz, $CDCl_3$) δ 148.23, 146.41, 140.59, 134.19, 132.39, 129.65, 128.51, 128.47, 128.02, 126.46, 126.09, 124.71, 117.48, 112.62, 110.35, 80.50, 64.41, 42.86, 15.05. HRMS (ESI-TOF) m/z Calcd for $C_{23}H_{22}ClN_2OS$ $[M+H]^+$: 409.1141, found: 409.1148.

2-phenyl-3-((2-phenyl-2-propoxy)ethylthio)imidazo[1,2-*a*]pyridine (3i)

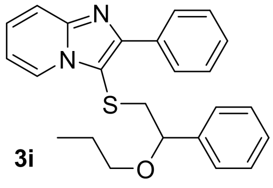

3i

Isolated yield 76%. Pale yellow oil. ^{1}H NMR (600 MHz, $CDCl_3$) δ 8.40 (d, *J* = 6.8 Hz, 1H), 8.24 (d, *J* = 7.1 Hz, 2H), 7.58 (d, *J* = 9.0 Hz, 1H), 7.39 (t, *J* = 7.8 Hz, 2H), 7.31 (t, *J* = 7.4 Hz, 1H), 7.22-7.14 (m, 4H), 7.03-7.02 (m, 2H), 6.81 (t, *J* = 6.7 Hz, 1H), 4.03 (dd, *J* = 8.5, 4.1 Hz, 1H), 3.05-3.02 (m, 1H), 2.92-2.88 (m, 2H), 2.78 (dd, *J* = 13.4, 4.1 Hz, 1H), 1.43-1.36 (m, 2H), 0.76 (t,

J = 7.4 Hz, 3H); ^{13}C NMR (151 MHz, $CDCl_3$) δ 149.42, 146.36, 140.79, 133.81, 128.47, 128.42, 128.35, 128.23, 127.93, 126.50, 125.93, 124.71, 117.45, 112.52, 110.29, 80.62, 70.79, 43.04, 22.93, 10.52. HRMS (ESI-TOF) m/z Calcd for $C_{24}H_{25}N_2OS$ $[M+H]^+$: 389.1688, found: 361.1713.

2-(4-methylphenyl)-3-((2-phenyl-2-propoxy)ethylthio)imidazo[1,2-*a*]pyridine (3j)

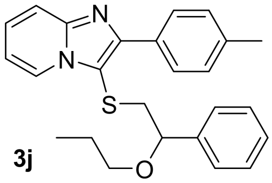

3j

Isolated yield 79%. Pale yellow oil. ^{1}H NMR (600 MHz, $CDCl_3$) δ 8.39 (d, J = 6.8 Hz, 1H), 8.14 (d, J = 8.1 Hz, 2H), 7.57 (d, J = 5.9 Hz, 1H), 7.21-7.16 (m, 6H), 7.03-7.02 (m, 2H), 6.79 (t, J = 6.6 Hz, 1H), 4.03 (dd, J = 8.5, 4.1 Hz, 1H), 3.05-3.01 (m, 1H), 2.91-2.85 (m, 2H), 2.77 (dd, J = 13.4, 4.1 Hz, 1H), 2.34 (s, 3H), 1.42-1.38 (m, 2H), 0.76 (t, J = 7.4 Hz, 3H); ^{13}C NMR (151 MHz, $CDCl_3$) δ 149.59, 146.35, 140.84, 138.06, 131.00, 129.07, 128.41, 128.33, 127.90, 126.52, 125.78, 124.65, 117.35, 112.37, 109.84, 80.61, 70.79, 43.01, 22.94, 21.34, 10.51. HRMS (ESI-TOF) m/z Calcd for $C_{25}H_{27}N_2OS$ $[M+H]^+$: 403.1844, found: 403.1866.

2-(4-chlorophenyl)-3-((2-phenyl-2-propoxy)ethylthio)imidazo[1,2-*a*]pyridine (3k)

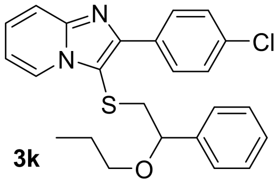

3k

Isolated yield 70%. Pale yellow oil. ^{1}H NMR (600 MHz, $CDCl_3$) δ 8.36 (d, J = 6.8 Hz, 1H), 8.20 (d, J = 8.5 Hz, 2H), 7.55 (d, J = 8.9 Hz, 1H), 7.34 (d, J = 8.5 Hz, 2H), 7.21-7.15 (m, 4H), 7.03-7.02 (m, 2H), 6.81 (t, J = 6.8 Hz, 1H), 4.04 (dd, J = 8.3, 4.1 Hz, 1H), 3.07-3.03 (m, 1H), 2.92-2.88 (m, 2H), 2.78 (dd, J = 13.4, 4.2 Hz, 1H), 2.34 (s, 3H), 1.43-1.36 (m, 2H), 0.76 (t, J = 7.4 Hz, 3H); ^{13}C NMR (151 MHz, $CDCl_3$) δ 148.21, 146.41, 140.65, 134.18, 132.41, 129.66, 128.51, 128.45, 127.99, 126.48, 126.07, 124.69, 117.49, 112.62, 110.43, 80.61, 70.82, 42.94, 22.93, 10.48. HRMS (ESI-TOF) m/z Calcd for $C_{24}H_{24}ClN_2OS$ $[M+H]^+$: 423.1298, found: 423.1309.

2-phenyl-3-((2-isopropoxy-2-phenyl)ethylthio)imidazo[1,2-*a*]pyridine (3l)

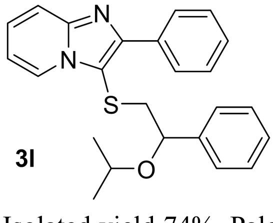

3l

Isolated yield 74%. Pale yellow oil. ^{1}H NMR (600 MHz, CD_3SOCD_3) δ 8.51 (d, J = 6.8 Hz, 1H), 8.24 (d, J = 7.2 Hz, 2H), 7.66 (d, J = 8.9 Hz, 1H), 7.48 (t, J = 7.8 Hz, 2H), 7.42-7.38 (m, 2H), 7.30-7.20 (m, 5H), 7.05 (t, J = 5.9 Hz, 1H), 4.49 (dd, J = 7.5, 4.4 Hz, 1H), 3.33 (septet, J = 6.1 Hz, 1H), 3.02 (dd, J = 13.1, 7.7 Hz, 1H), 2.95 (dd, J = 13.0, 4.4 Hz, 1H), 0.94 (d, J = 6.0 Hz, 3H), 0.90 (d, J = 6.2 Hz, 3H); ^{13}C NMR (151 MHz, $CDCl_3$) δ 148.34, 146.12, 141.70, 134.26, 128.77, 128.72, 128.55, 128.45, 128.13, 126.90, 125.57, 117.38, 113.38, 110.25, 77.40, 69.24, 43.05, 23.36, 21.39. HRMS (ESI-TOF) m/z Calcd for $C_{24}H_{24}N_2OS$ M^+: 388.1609, found: 388.1612.

3-((2-isopropoxy-2-phenyl)ethylthio)-2-(4-methylphenyl)imidazo[1,2-*a*]pyridine (3m)

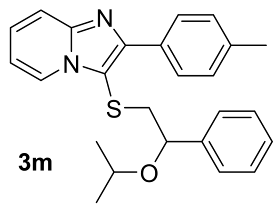

3m

Isolated yield 75%. Pale yellow oil. ^{1}H NMR (600 MHz, $CDCl_3$) δ 8.34 (d, *J* = 6.8 Hz, 1H), 8.10 (d, *J* = 8.2 Hz, 2H), 7.58 (d, *J* = 8.9 Hz, 1H), 7.21-7.16 (m, 6H), 7.08-7.06 (m, 2H), 6.80 (t, *J* = 6.7 Hz, 1H), 4.29 (dd, *J* = 7.9, 4.7 Hz, 1H), 3.33 (septet, *J* = 6.1 Hz, 1H), 2.92 (dd, *J* = 13.1, 7.9 Hz, 1H), 2.78 (dd, *J* = 13.1, 4.7 Hz, 1H), 2.34 (S, 3H), 0.96 (d, *J* = 6.0 Hz, 3H), 0.92 (d, *J* = 6.2 Hz, 3H); ^{13}C NMR (151 MHz, $CDCl_3$) δ 149.02, 146.09, 141.38, 138.13, 130.79, 129.10, 128.38, 128.34, 127.83, 126.58, 124.78, 117.23, 112.46, 110.32, 77.98, 69.54, 43.54, 23.16, 21.34, 21.07. HRMS (ESI-TOF) m/z Calcd for $C_{25}H_{27}N_2OS$ $[M+H]^+$: 403.1844, found: 403.1875.

2-(4-chlorophenyl)-3-((2-isopropoxy-2-phenyl)ethylthio)imidazo[1,2-*a*]pyridine (3n)

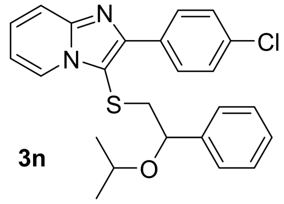

3n

Isolated yield 69%. Pale yellow oil. ^{1}H NMR (600 MHz, $CDCl_3$) δ 8.34 (d, *J* = 6.8 Hz, 1H), 8.17 (d, *J* = 8.5 Hz, 2H), 7.56 (d, *J* = 8.9 Hz, 1H), 7.35 (d, *J* = 8.6 Hz, 2H), 7.22-7.16 (m, 4H. The peak of $CDCl_3$ overlap with it), 7.08-7.07 (m, 2H), 6.81 (t, *J* = 6.7 Hz, 1H), 4.31 (dd, *J* = 7.9, 4.7 Hz, 1H), 3.35 (septet, *J* = 6.1 Hz, 1H), 2.93 (dd, *J* = 13.0, 7.9 Hz, 1H), 2.79 (dd, *J* = 13.0, 4.7 Hz, 1H), 0.98 (d, *J* = 6.0 Hz, 3H), 0.93 (d, *J* = 6.2 Hz, 3H); ^{13}C NMR (151 MHz, $CDCl_3$) δ 147.90, 146.29, 141.22, 134.17, 132.38, 129.70, 128.52, 128.38, 127.91, 126.54, 126.02, 124.79, 117.44, 112.60, 110.79, 77.92, 69.56, 43.48, 23.13, 21.06. HRMS (ESI-TOF) m/z Calcd for $C_{24}H_{24}ClN_2OS$ $[M+H]^+$: 423.1298, found: 423.1303.

3-((2-butoxy-2-phenyl)ethylthio)-2-phenylimidazo[1,2-*a*]pyridine (3o)

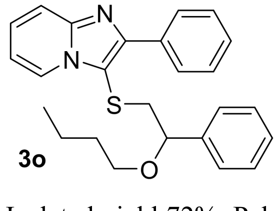

3o

Isolated yield 72%. Pale yellow oil. ^{1}H NMR (600 MHz, $CDCl_3$) δ 8.39 (d, *J* = 6.8 Hz, 1H), 8.24 (d, *J* = 8.2 Hz, 2H), 7.57 (d, *J* = 8.9 Hz, 1H), 7.39 (t, *J* = 7.7 Hz, 2H), 7.30 (t, *J* = 7.4 Hz, 1H), 7.21-7.14 (m, 4H), 7.02-7.01 (m, 2H), 6.80 (t, *J* = 6.8 Hz, 1H), 4.02 (dd, *J* = 8.5, 4.1 Hz, 1H), 3.09-3.05 (m, 1H), 2.93-2.88 (m, 2H), 2.77 (dd, *J* = 13.4, 4.1 Hz, 1H), 1.40-1.30 (m, 2H), 1.22-1.18 (m, 2H), 0.77 (t, *J* = 7.4 Hz, 3H); ^{13}C NMR (151 MHz, $CDCl_3$) δ 149.46, 146.40, 140.79, 133.87, 128.45, 128.44, 128.36, 128.23, 127.94, 126.51, 125.91, 124.71, 117.47, 112.51, 110.25, 80.64, 68.94, 43.05, 31.84, 19.30, 13.93. HRMS (ESI-TOF) m/z Calcd for $C_{25}H_{27}N_2OS$ $[M+H]^+$: 403.1844, found: 403.1850.

3-((2-butoxy-2-phenyl)ethylthio)-2-(4-methylphenyl)imidazo[1,2-*a*]pyridine (3p)

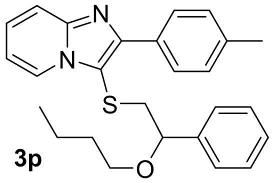

3p

Isolated yield 74%. Pale yellow oil. ^{1}H NMR (600 MHz, CDCl$_3$) δ 8.44 (d, *J* = 6.8 Hz, 1H), 8.21 (d, *J* = 8.2 Hz, 2H), 7.63 (d, *J* = 8.4 Hz, 1H), 7.28-7.21 (m, 6H), 7.10-7.09 (m, 2H), 6.86 (t, *J* = 7.5 Hz, 1H), 4.09 (dd, *J* = 8.5, 4.1 Hz, 1H), 3.17-3.13 (m, 1H), 3.01-2.95 (m, 2H), 2.84 (dd, *J* = 13.4, 4.1 Hz, 1H), 2.41 (s, 3H), 1.48-1.38 (m, 2H), 1.30-1.24 (m, 2H), 0.85 (t, *J* = 7.4 Hz, 3H); ^{13}C NMR (151 MHz, CDCl$_3$) δ 149.60, 146.37, 140.85, 138.03, 131.05, 129.07, 128.41, 128.32, 127.90, 126.51, 125.74, 124.65, 117.36, 112.36, 109.84, 80.66, 68.94, 43.00, 31.86, 21.34, 19.29, 13.90. HRMS (ESI-TOF) m/z Calcd for $C_{26}H_{29}N_2OS$ [M+H]$^+$: 417.2001, found: 417.2023.

3-((2-butoxy-2-phenyl)ethylthio)-2-(4-chlorophenyl)imidazo[1,2-*a*]pyridine (**3q**)

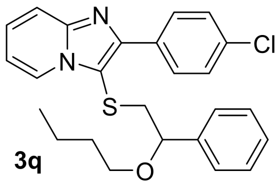

3q

Isolated yield 60%. Pale yellow oil. ^{1}H NMR (600 MHz, CDCl$_3$) δ 8.36 (d, *J* = 6.8 Hz, 1H), 8.21 (d, *J* = 8.6 Hz, 2H), 7.55 (d, *J* = 8.9 Hz, 1H), 7.34 (d, *J* = 8.5 Hz, 2H), 7.20-7.16 (m, 4H), 7.03-7.02 (m, 2H), 6.81 (t, *J* = 6.7 Hz, 1H), 4.04 (dd, *J* = 8.4, 4.1 Hz, 1H), 3.12-3.08 (m, 1H), 2.96-2.88 (m, 2H), 2.78 (dd, *J* = 13.3, 4.2 Hz, 1H), 1.40-1.31 (m, 2H), 1.24-1.18 (m, 2H), 0.78 (t, *J* = 7.4 Hz, 3H); ^{13}C NMR (151 MHz, CDCl$_3$) δ 148.20, 146.41, 140.66, 134.18, 132.43, 129.65, 128.51, 128.44, 127.99, 126.47, 126.05, 124.69, 117.49, 112.62, 110.43, 80.65, 68.96, 42.92, 31.83, 19.26, 13.83. HRMS (ESI-TOF) m/z Calcd for $C_{25}H_{26}ClN_2OS$ [M+H]$^+$: 437.1454, found: 437.1467.

3-((2-methoxy-2-phenyl)ethylthio)-8-methyl-2-phenylimidazo[1,2-*a*]pyridine (**3s**)

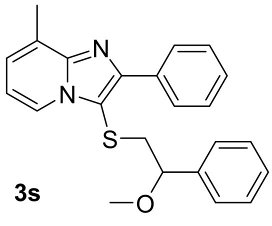

3s

Isolated yield 80%. Pale yellow oil. ^{1}H NMR (600 MHz, CDCl$_3$) δ 8.24 (t, *J* = 7.5 Hz, 3H), 7.38 (t, *J* = 8.1 Hz, 2H), 7.29 (t, *J* = 7.4 Hz, 1H), 7.18-7.14 (m, 3H), 7.00-6.98 (m, 3H), 6.72 (t, *J* = 6.8 Hz, 1H), 3.90-3.86 (m, 1H), 2.91 (s, 3H), 2.84 (dd, *J* = 13.5, 8.7 Hz, 1H), 2.75 (dd, *J* = 13.6, 4.1 Hz, 1H), 2.60 (s, 3H); ^{13}C NMR (151 MHz, CDCl$_3$) δ 149.42, 146.82, 140.10, 134.22, 128.62, 128.46, 128.30, 128.03, 127.54, 126.60, 124.64, 122.44, 112.47, 110.16, 82.22, 56.61, 42.89, 16.71. HRMS (ESI-TOF) m/z Calcd for $C_{23}H_{23}N_2OS$ [M+H]$^+$: 375.1531, found: 375.1525.

3-((2-methoxy-2-phenyl)ethylthio)-7-methyl-2-phenylimidazo[1,2-*a*]pyridine (**3t**)

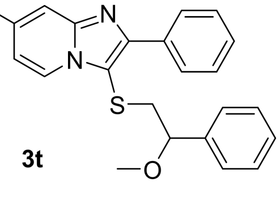

3t

Isolated yield 73%. Pale yellow oil. ^{1}H NMR (600 MHz, $CDCl_3$) δ 8.26 (d, *J* = 7.0 Hz, 1H), 8.23 (d, *J* = 7.3 Hz, 2H), 7.39 (t, *J* = 7.8 Hz, 2H), 7.34-7.33 (m, 1H), 7.30-7.28 (m, 1H), 7.19-7.16 (m, 3H), 7.01-7.00 (m, 2H), 6.65 (d, *J* = 6.9 Hz, 1H), 3.88 (dd, *J* = 8.8, 4.0 Hz, 1H), 2.92 (s, 3H), 2.84 (dd, *J* = 13.6, 8.8 Hz, 1H), 2.75 (dd, *J* = 13.6, 4.0 Hz, 1H), 2.36 (s, 3H); ^{13}C NMR (151 MHz, $CDCl_3$) δ 149.63, 146.88, 140.07, 137.04, 134.03, 128.48, 128.40, 128.29, 128.08, 128.06, 126.59, 123.83, 116.04, 115.11, 109.14, 82.16, 56.60, 42.99, 21.30. HRMS (ESI-TOF) m/z Calcd for $C_{23}H_{23}N_2OS$ $[M+H]^+$: 375.1531, found: 375.1529.

3-((2-methoxy-2-phenyl)ethylthio)-6-methyl-2-phenylimidazo[1,2-*a*]pyridine (3u)

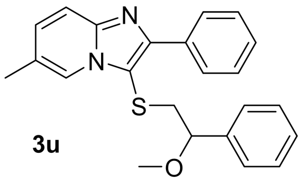

3u

Isolated yield 68%. Pale yellow oil. ^{1}H NMR (600 MHz, $CDCl_3$) δ 8.24 (d, *J* = 7.4 Hz, 2H), 8.18-8.17 (m, 1H), 7.48 (d, *J* = 9.1 Hz, 1H), 7.39 (t, *J* = 7.7 Hz, 2H), 7.30-7.28 (m, 1H), 7.19-7.16 (m, 3H), 7.06 (d, *J* = 9.0 Hz, 1H), 7.01 (d, *J* = 6.5 Hz, 2H), 3.88 (dd, *J* = 8.7, 4.1 Hz, 1H), 2.93 (s, 3H), 2.86 (dd, *J* = 13.6, 8.7 Hz, 1H), 2.78 (dd, *J* = 13.6, 4.1 Hz, 1H), 2.30 (s, 3H); ^{13}C NMR (151 MHz, $CDCl_3$) δ 149.56, 145.52, 140.01, 134.02, 129.02, 128.48, 128.36, 128.31, 128.10, 126.63, 122.48, 122.23, 116.84, 109.41, 82.15, 56.59, 42.90, 18.37. HRMS (ESI-TOF) m/z Calcd for $C_{23}H_{23}N_2OS$ $[M+H]^+$: 375.1531, found: 375.1536.

3-((2-methoxy-2-(2-methyl)phenyl)ethylthio)-2-phenylimidazo[1,2-*a*]pyridine (4a)

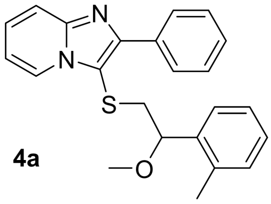

4a

Isolated yield 76%. Pale yellow oil. ^{1}H NMR (600 MHz, $CDCl_3$) δ 8.45 (d, *J* = 6.8 Hz, 1H), 8.30-8.28 (m, 2H), 7.60 (d, *J* = 9.0 Hz, 1H), 7.40 (t, *J* = 7.9 Hz, 2H), 7.30 (t, *J* = 7.3 Hz, 1H), 7.24-7.21 (m, 1H), 7.18-7.16 (m, 1H), 7.06 (t, *J* = 7.3 Hz, 1H), 7.02 (t, *J* = 8.6 Hz, 1H), 6.90 (d, *J* = 7.3 Hz, 1H), 6.84 (t, *J* = 3.2 Hz, 1H), 4.07 (dd, *J* = 8.9, 3.1 Hz, 1H), 2.84 (s, 3H), 2.74-2.66 (m, 2H), 1.69 (s, 3H); ^{13}C NMR (151 MHz, $CDCl_3$) δ 149.90, 146.49, 137.95, 135.12, 133.75, 130.54, 128.49, 128.39, 128.32, 127.57, 126.30, 126.01, 125.37, 124.63, 117.53, 112.61, 110.00, 78.46, 56.60, 41.77, 18.15. HRMS (ESI-TOF) m/z Calcd for $C_{23}H_{23}N_2OS$ $[M+H]^+$: 375.1531, found: 375.1544.

3-((2-ethoxy-2-(2-methyl)phenyl)ethylthio)-2-phenylimidazo[1,2-*a*]pyridine (4b)

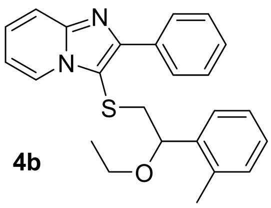

4b

Isolated yield 70%. Pale yellow oil. ^{1}H NMR (600 MHz, $CDCl_3$) δ 8.43 (d, *J* = 6.8 Hz, 1H), 8.26 (d, *J* = 7.8 Hz, 2H), 7.57 (d, *J* = 8.9 Hz, 1H), 7.38 (t, *J* = 7.7 Hz, 2H), 7.28 (t, *J* = 7.3 Hz, 1H), 7.20-7.18 (m, 2H), 7.04 (t, *J* = 7.6 Hz, 1H), 7.00 (t, *J* = 7.3 Hz, 1H), 6.88 (d, *J* = 7.4 Hz, 1H), 6.81 (t, *J* = 6.7 Hz, 1H), 4.19 (dd, *J* = 9.2, 2.5 Hz, 1H), 3.10-3.05 (m, 1H), 2.80-2.73 (m, 2H), 2.68 (dd,

J = 13.7, 2.6 Hz, 1H), 1.70 (s, 3H), 0.97 (t, J = 7.0 Hz, 1H); ^{13}C NMR (151 MHz, $CDCl_3$) δ 149.79, 146.46, 138.77, 134.93, 133.82, 130.47, 128.50, 128.32, 128.24, 127.44, 126.23, 125.90, 125.48, 124.65, 117.50, 112.51, 110.22, 76.94, 64.38, 41.80, 18.15, 15.11. HRMS (ESI-TOF) m/z Calcd for $C_{24}H_{25}N_2OS$ $[M+H]^+$: 389.1688, found: 389.1697.

3-((2-methoxy-2-(3-methyl)phenyl)ethylthio)-2-phenylimidazo[1,2-*a*]pyridine (4c)

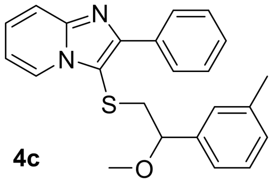

4c

Isolated yield 78%. Pale yellow oil. ^{1}H NMR (600 MHz, $CDCl_3$) δ 8.39 (d, J = 6.8 Hz, 1H), 8.26 (d, J = 7.4 Hz, 2H), 7.58 (d, J = 8.9 Hz, 1H), 7.40 (t, J = 7.7 Hz, 2H), 7.30 (t, J = 7.4 Hz, 1H), 7.21 (t, J = 7.6 Hz, 1H), 7.06 (t, J = 7.4 Hz, 1H), 6.96 (d, J = 7.4 Hz, 1H), 6.83-6.80 (m, 3H), 3.86 (dd, J = 8.7, 4.1 Hz, 1H), 2.92 (s, 3H), 2.86 (dd, J = 13.6, 8.7 Hz, 1H), 2.78 (dd, J = 13.6, 4.1 Hz, 1H), 2.20 (s, 3H); ^{13}C NMR (151 MHz, $CDCl_3$) δ 149.65, 146.45, 139.87, 138.19, 133.87, 128.86, 128.45, 128.38, 128.35, 128.24, 127.22, 125.91, 124.71, 123.68, 117.47, 112.47, 110.03, 82.26, 56.61, 42.82, 21.33. HRMS (ESI-TOF) m/z Calcd for $C_{23}H_{23}N_2OS$ $[M+H]^+$: 375.1531, found: 375.1527.

3-((2-ethoxy-2-(3-methyl)phenyl)ethylthio)-2-phenylimidazo[1,2-*a*]pyridine (4d)

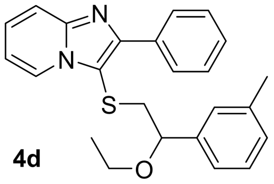

4d

Isolated yield 72%. Pale yellow oil. ^{1}H NMR (600 MHz, $CDCl_3$) δ 8.38 (d, J = 6.8 Hz, 1H), 8.24 (d, J = 7.9 Hz, 2H), 7.56 (d, J = 8.9 Hz, 1H), 7.38 (t, J = 7.7 Hz, 2H), 7.29 (t, J = 7.4 Hz, 1H), 7.20-7.18 (m, 1H), 7.05 (t, J = 7.5 Hz, 1H), 6.94 (d, J = 7.4 Hz, 1H), 6.83-6.78 (m, 3H), 3.99 (dd, J = 8.5, 4.3 Hz, 1H), 3.16-3.13 (m, 1H), 2.98-2.95 (m, 1H), 2.89 (dd, J = 13.5, 8.5 Hz, 1H), 2.78 (dd, J = 13.4, 4.3 Hz, 1H), 2.19 (s, 3H), 0.98 (t, J = 7.0 Hz, 3H); ^{13}C NMR (151 MHz, $CDCl_3$) δ 149.48, 146.39, 140.66, 138.08, 133.90, 128.69, 128.46, 128.30, 128.18, 127.15, 124.74, 123.58, 117.43, 112.39, 110.28, 80.61, 64.36, 42.92, 21.31, 15.08. HRMS (ESI-TOF) m/z Calcd for $C_{24}H_{25}N_2OS$ $[M+H]^+$: 389.1688, found: 389.1653.

3-((2-methoxy-2-(4-methyl)phenyl)ethylthio)-2-phenylimidazo[1,2-*a*]pyridine (4e)

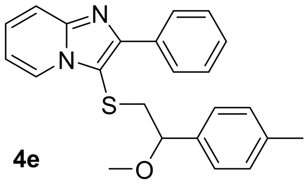

4e

Isolated yield 80%. Pale yellow oil. ^{1}H NMR (600 MHz, $CDCl_3$) δ 8.39 (d, J = 6.8 Hz, 1H), 8.24 (d, J = 7.2 Hz, 2H), 7.58 (d, J = 9.0 Hz, 1H), 7.40 (t, J = 7.8 Hz, 2H), 7.31 (t, J = 7.4 Hz, 1H), 7.23-7.20 (m, 1H), 6.98 (d, J = 7.9 Hz, 2H), 6.89 (d, J = 8.0 Hz, 2H), 6.82 (t, J = 6.7 Hz, 1H), 3.86 (dd, J = 8.7, 4.1 Hz, 1H), 2.91 (s, 3H), 2.86 (dd, J = 13.6, 8.8 Hz, 1H), 2.76 (dd, J = 13.6, 4.2 Hz, 1H), 2.22 (s, 3H); ^{13}C NMR (151 MHz, $CDCl_3$) δ 149.63, 146.44, 137.90, 136.83, 133.82, 129.18, 128.45, 128.37, 128.26, 126.56, 125.97, 124.71, 117.45, 112.51, 110.03, 81.98, 56.50, 42.82,

21.12. HRMS (ESI-TOF) m/z Calcd for $C_{23}H_{23}N_2OS$ $[M+H]^+$: 375.1531, found: 375.1523.

3-((2-ethoxy-2-(4-methyl)phenyl)ethylthio)-2-phenylimidazo[1,2-*a*]pyridine (4f)

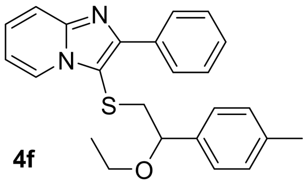

Isolated yield 76%. Pale yellow oil. ^{1}H NMR (600 MHz, $CDCl_3$) δ 8.38 (d, *J* = 6.8 Hz, 1H), 8.23 (d, *J* = 7.3 Hz, 2H), 7.56 (d, *J* = 8.9 Hz, 1H), 7.38 (t, *J* = 7.8 Hz, 2H), 7.30 (t, *J* = 7.4 Hz, 1H), 7.21-7.17(m, 1H), 6.97 (d, *J* = 7.8 Hz, 2H), 6.90 (d, *J* = 7.9 Hz, 2H), 6.80 (t, *J* = 6.7 Hz, 1H), 4.00 (dd, *J* = 8.3, 4.3 Hz, 1H), 3.14-3.12 (m, 1H), 2.97-2.94 (m, 1H), 2.89 (dd, *J* = 13.4, 8.5 Hz, 1H), 2.77 (dd, *J* = 13.4, 4.3 Hz, 1H), 2.21 (s, 3H), 0.97 (t, *J* = 7.0 Hz, 3H); ^{13}C NMR (151 MHz, $CDCl_3$) δ 149.48, 146.39, 137.67, 133.90, 129.48, 129.10, 128.45, 128.30, 128.18, 126.46, 125.80, 124.74, 117.43, 112.38, 110.28, 80.36, 64.24, 42.92, 21.05, 15.07. HRMS (ESI-TOF) m/z Calcd for $C_{24}H_{25}N_2OS$ $[M+H]^+$: 389.1688, found: 389.1688.

3-((2-(2-chlorophenyl)-2-methoxy)ethylthio)-2-phenylimidazo[1,2-*a*]pyridine (4g)

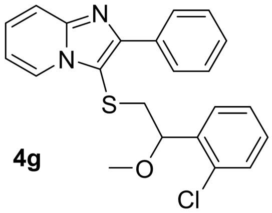

Isolated yield 76%. Pale yellow oil. ^{1}H NMR (600 MHz, $CDCl_3$) δ 8.44 (d, *J* = 6.8 Hz, 1H), 8.26 (d, *J* = 8.2 Hz, 2H), 7.60 (d, *J* = 8.9 Hz, 1H), 7.40 (t, *J* = 7.9 Hz, 2H), 7.31-7.21 (m, 3H), 7.14-7.05(m, 3H), 6.84 (t, *J* = 5.7 Hz, 1H), 4.35 (dd, *J* = 9.4, 2.4 Hz, 1H), 2.87 (s, 3H), 2.85 (dd, *J* = 13.7, 2.5 Hz, 1H), 2.63 (dd, *J* = 13.7, 9.4 Hz, 1H); ^{13}C NMR (151 MHz, $CDCl_3$) δ 150.14, 146.50, 137.44, 133.78, 132.71, 129.60, 128.87, 128.63, 128.31, 128.25, 127.09, 126.89, 125.99, 124.63, 117.52, 112.55, 109.69, 78.47, 57.07, 41.03. HRMS (ESI-TOF) m/z Calcd for $C_{22}H_{20}ClN_2OS$ $[M+H]^+$: 395.0985, found: 395.0997.

3-((2-(2-chlorophenyl)-2-ethoxy)ethylthio)-2-phenylimidazo[1,2-*a*]pyridine (4h)

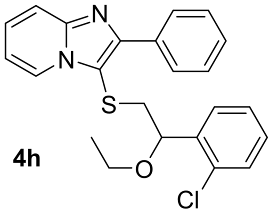

Isolated yield 66%. Pale yellow oil. ^{1}H NMR (600 MHz, $CDCl_3$) δ 8.43 (d, *J* = 6.8 Hz, 1H), 8.25 (d, *J* = 7.3 Hz, 2H), 7.58 (d, *J* = 9.0 Hz, 1H), 7.39 (t, *J* = 7.8 Hz, 2H), 7.30-7.28 (m, 2H), 7.22-7.19 (m, 1H), 7.13-7.09 (m, 2H), 7.06-7.03 (m, 1H), 6.82 (t, *J* = 6.8 Hz, 1H), 4.48 (dd, *J* = 9.2, 2.4 Hz, 1H), 3.13-3.08 (m, 1H), 2.89-2.83 (m, 2H), 2.68 (dd, *J* = 13.6, 9.2 Hz, 1H), 0.97 (t, *J* = 7.0 Hz, 3H); ^{13}C NMR (151 MHz, $CDCl_3$) δ 150.04, 146.49, 138.26, 133.90, 132.57, 129.50, 128.73, 128.62, 128.25, 128.15, 127.02, 125.82, 124.66, 117.53, 112.43, 109.90, 76.85, 64.96, 41.14, 15.06. HRMS (ESI-TOF) m/z Calcd for $C_{23}H_{22}ClN_2OS$ $[M+H]^+$: 409.1141, found: 409.1142.

3-((2-(3-chlorophenyl)-2-methoxy)ethylthio)-2-phenylimidazo[1,2-*a*]pyridine (4i)

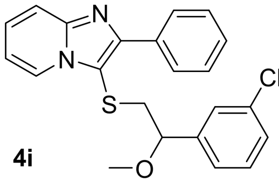

4i

Isolated yield 73%. Pale yellow oil. ^{1}H NMR (600 MHz, $CDCl_3$) δ 8.46 (d, J = 6.8 Hz, 1H), 8.31-8.30 (m, 2H), 7.68 (d, J = 8.9 Hz, 1H), 7.49 (t, J = 7.9 Hz, 2H), 7.40 (t, J = 7.4 Hz, 1H), 7.32-7.29 (m, 1H), 7.21-7.15 (m, 2H), 7.09-7.08 (m, 1H), 7.06-7.03 (m, 1H), 6.93 (t, J = 7.0 Hz, 2H), 3.93 (dd, J = 8.4, 4.4 Hz, 1H), 2.98 (s, 3H), 2.90 (dd, J = 13.6, 8.4 Hz, 1H), 2.82 (dd, J = 13.7, 4.4 Hz, 1H); ^{13}C NMR (151 MHz, $CDCl_3$) δ 149.64, 146.39, 142.13, 134.53, 133.61, 129.79, 128.49, 128.43, 128.41, 128.28, 126.69, 124.75, 124.59, 117.51, 112.71, 109.73, 81.72, 56.79, 42.53. HRMS (ESI-TOF) m/z Calcd for $C_{22}H_{20}ClN_2OS$ $[M+H]^+$: 395.0985, found: 395.0985.

3-((2-(3-chlorophenyl)-2-ethoxy)ethylthio)-2-phenylimidazo[1,2-*a*]pyridine (4j)

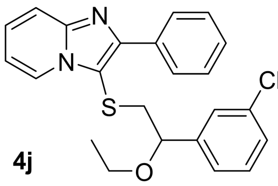

4j

Isolated yield 69%. Pale yellow oil. ^{1}H NMR (600 MHz, $CDCl_3$) δ 8.37 (d, J = 6.8 Hz, 1H), 8.22 (d, J = 8.0 Hz, 2H), 7.57 (d, J = 8.9 Hz, 1H), 7.40 (t, J = 7.7 Hz, 2H), 7.31 (t, J = 7.2 Hz, 1H), 7.22-7.18 (m, 1H), 7.11-7.06 (m, 2H), 7.09-7.08 (m, 1H), 7.06-7.03 (m, 1H), 6.85 (d, J = 7.4 Hz, 1H), 6.82 (t, J = 6.7 Hz, 1H), 3.99 (dd, J = 8.1, 4.4 Hz, 1H), 3.14-3.09 (m, 1H), 2.98-2.93 (m, 1H), 2.85 (dd, J = 13.6, 8.2 Hz, 1H), 2.75 (dd, J = 13.5, 4.4 Hz, 1H), 0.97 (s, 3H); ^{13}C NMR (151 MHz, $CDCl_3$) δ 149.64, 146.44, 142.92, 134.47, 133.82, 129.70, 128.47, 128.36, 128.29, 128.09, 126.62, 125.91, 124.65, 124.59, 117.53, 112.54, 109.88, 80.07, 64.63, 42.61, 15.02. HRMS (ESI-TOF) m/z Calcd for $C_{23}H_{22}ClN_2OS$ $[M+H]^+$: 409.1141, found: 409.1141.

3-((2-(4-chlorophenyl)-2-methoxy)ethylthio)-2-phenylimidazo[1,2-*a*]pyridine (4k)

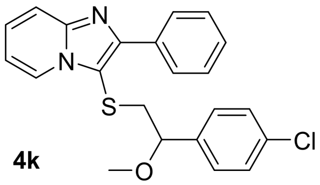

4k

Isolated yield 76%. Pale yellow oil. ^{1}H NMR (600 MHz, CD_3SOCD_3) δ 8.53 (d, J = 6.8 Hz, 1H), 8.24 (d, J = 7.9 Hz, 2H), 7.66 (d, J = 8.9 Hz, 1H), 7.48 (t, J = 7.7 Hz, 2H), 7.44-7.39 (m, 2H), 7.32 (d, J = 8.3 Hz, 2H), 7.18 (d, J = 8.3 Hz, 2H), 7.07 (t, J = 6.7 Hz, 1H), 4.2 (t, J = 6.2 Hz, 1H), 3.01 (d, J = 6.2 Hz, 2H), 2.96 (s, 3H); ^{13}C NMR (151 MHz, $CDCl_3$) δ 148.64, 146.21, 139.31, 134.19, 132.90, 128.95, 128.77, 128.59, 128.37, 127.05, 125.51, 125.91, 117.38, 113.41, 109.69, 81.29, 56.58, 41.96. HRMS (ESI-TOF) m/z Calcd for $C_{22}H_{20}ClN_2OS$ $[M+H]^+$: 395.0985, found: 395.0973.

3-((2-(4-chlorophenyl)-2-ethoxy)ethylthio)-2-phenylimidazo[1,2-*a*]pyridine (4l)

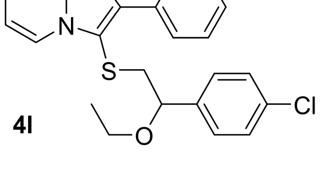

4l

Isolated yield 70%. Pale yellow oil. ^{1}H NMR (600 MHz, $CDCl_3$) δ 8.35 (d, *J* = 6.4 Hz, 1H), 8.21 (d, *J* = 7.6 Hz, 2H), 7.57 (d, *J* = 8.8 Hz, 1H), 7.40-7.38 (m, 2H), 7.32-7.29 (m, 1H), 7.21-7.19 (m, 1H), 7.11 (d, *J* = 7.9 Hz, 2H), 6.92 (d, *J* = 7.4 Hz, 2H), 6.82-6.80 (m, 1H), 3.99-3.97 (m, 1H), 3.10-3.08 (m, 1H), 2.96-2.93 (m, 1H), 2.86-2.84 (m, 1H), 2.75-2.73 (m, 1H), 0.96 (t, *J* = 6.8 Hz, 3H); ^{13}C NMR (151 MHz, $CDCl_3$) δ 149.64, 146.44, 139.18, 133.84, 133.69, 128.60, 128.47, 128.35, 128.27, 127.88, 125.90, 124.59, 117.54, 112.51, 109.89, 79.91, 64.48, 42.59, 15.04. HRMS (ESI-TOF) m/z Calcd for $C_{23}H_{22}ClN_2OS$ $[M+H]^+$: 409.1141, found: 409.1143.

3-((2-(2-bromophenyl)-2-methoxy)ethylthio)-2-phenylimidazo[1,2-*a*]pyridine (4m)

4m

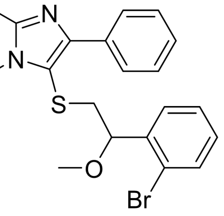

Isolated yield 73%. Pale yellow oil. ^{1}H NMR (600 MHz, $CDCl_3$) δ 8.45 (d, *J* = 6.8 Hz, 1H), 8.26 (d, *J* = 7.3 Hz, 2H), 7.60 (d, *J* = 8.9 Hz, 1H), 7.41 (t, *J* = 7.8 Hz, 2H), 7.31-7.16 (m, 5H), 6.98 (td, *J* = 7.9, 1.6 Hz, 1H), 6.85 (t, *J* = 7.4 Hz, 2H), 4.24 (dd, *J* = 9.6, 1.9 Hz, 1H), 2.82 (s, 3H), 2.84-2.81 (m, 1H), 2.55 (dd, *J* = 13.9, 9.7 Hz, 1H); ^{13}C NMR (151 MHz, $CDCl_3$) δ 150.43, 146.57, 138.93, 133.83, 132.89, 129.21, 128.72, 128.33, 128.26, 127.73, 127.06, 126.02, 124.62, 122.68, 117.56, 112.60, 109.60, 80.56, 57.05, 41.08. HRMS (ESI-TOF) m/z Calcd for $C_{22}H_{20}BrN_2OS$ $[M+H]^+$: 439.0480, found: 439.0470.

3-((2-(3-bromophenyl)-2-methoxy)ethylthio)-2-phenylimidazo[1,2-*a*]pyridine (4n)

4n

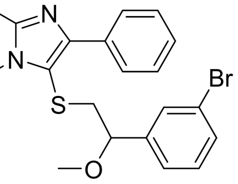

Isolated yield 75%. Pale yellow oil. ^{1}H NMR (600 MHz, CD_3SOCD_3) δ 8.53 (d, *J* = 6.8 Hz, 1H), 8.26 -8.24 (m, 2H), 7.66 (d, *J* = 8.9 Hz, 1H), 7.48 (t, *J* = 7.8 Hz, 2H), 7.45-7.37 (m, 4H), 7.24 (t, *J* = 7.8 Hz, 1H), 7.17 (d, *J* = 7.7 Hz, 1H), 7.08 (td, *J* = 6.8, 1.1 Hz, 1H), 4.22 (t, *J* = 5.5 Hz, 1H), 3.04-3.03 (m, 2H), 2.97 (s, 3H); ^{13}C NMR (151 MHz, $CDCl_3$) δ 153.39, 150.96, 147.96, 138.93, 135.99, 135.75, 134.53, 133.53, 133.36, 133.13, 131.82, 130.87, 130.26, 126.97, 122.12, 118.18, 114.40, 86.06, 61.46, 46.66. HRMS (ESI-TOF) m/z Calcd for $C_{22}H_{20}BrN_2OS$ $[M+H]^+$: 439.0480, found: 439.0470.

4. Bioactivity assay results of products 3a and 3b

The inhibitory effects of two synthesized compounds **3a** and **3b** against the target protease were determined via a fluorescence-based enzymatic inhibition assay. Different concentration gradients were designed for each compound: 0, 1, 5, 10, 20, 50, 100 and 200 μM for **3a**, and 0, 2, 8, 15, 30, 60, 120 and 250 μM for **3b**. Three technical replicates were set for every concentration point to guarantee data reproducibility. Outliers were removed using the Grubbs test ($p < 0.05$), and four-parameter logistic (4-PL) nonlinear regression was applied to fit the dose–response curves.

Both compounds exerted evident concentration-dependent inhibitory effects on the target protease. For compound **3a**, the relative enzyme activity decreased progressively from 100.00 ± 0.40% at 0 μM (blank control) to 4.28 ± 0.16% at 200 μM. The residual enzyme activity was 98.56 ± 0.36% (1 μM), 88.07 ± 0.44% (5 μM), 75.23 ± 0.48% (10 μM), 50.76 ± 0.50% (20 μM), 26.55 ± 0.33% (50 μM) and 11.02 ± 0.28% (100 μM). All standard deviation (SD) values were less than 0.50%, suggesting low data dispersion and stable experimental performance.

For compound **3b**, the relative enzyme activity declined from 100.00 ± 0.38% at 0 μM to 3.86 ± 0.14% at 250 μM. At the tested concentrations of 2, 8, 15, 30, 60, 120 and 250 μM, the residual activity was 97.25 ± 0.32%, 85.18 ± 0.42%, 68.36 ± 0.47%, 49.82 ± 0.53%, 23.15 ± 0.30%, 9.64 ± 0.27% and 3.86 ± 0.14%, respectively. The maximum SD across all groups was 0.53%, which confirmed favorable repeatability of the assay system.

Sigmoidal dose–response curves were obtained for both compounds after 4-PL fitting. The key inhibitory parameters are summarized as follows. Compound **3a** gave an IC_{50} value of 20.10 μM with a 95% confidence interval (CI) of 18.50–22.00 μM, a coefficient of determination (R^2) of 0.988, and a Hill slope of 1.25. For compound **3b**, the IC_{50} was 29.75 μM (95% CI: 27.62–32.08 μM), with an R^2 of 0.991 and a Hill slope of 1.31. The narrow confidence intervals indicated high accuracy of IC_{50} values. The R^2 values above 0.95 reflected excellent consistency between experimental data and the fitted model. Additionally, the Hill slopes of both compounds ranged from 0.5 to 2.0, demonstrating specific and cooperative binding between the compounds and target protease, with no obvious non-specific inhibition.

In conclusion, both **3a** and **3b** possessed potent concentration-dependent inhibitory activity against the target protease. Comparatively, compound **3a** exhibited stronger inhibitory potency than **3b**. The full set of reliable data provides a solid basis for subsequent structure–activity relationship analysis, structural modification and mechanistic research.

5. Microplate reader assay data of 3a and 3b

Characterization Category	Characterization Item	Specific Information	Remarks
**Enzyme Characterization**	abbreviation of the enzyme	Aurora A kinase	Target protease
**Enzyme Characterization**	Enzyme source	Prokaryotic expression and purification in laboratory	Self-purified sample
**Enzyme Characterization**	Enzyme purity	≥95% as determined by SDS-PAGE	Protein purity indicator
**Enzyme Characterization**	Working concentration of the enzyme	0.2 U/mL	Working concentration for experiments
**Enzyme Characterization**	Enzyme storage and pre-treatment conditions	Stored in aliquots at -80°C; thawed in water bath before use	Ensures enzyme activity stability
**Small Molecule Compound Characterization**	Compound name	Small molecule inhibitor **3a** & **3b**	Test sample
**Small Molecule Compound Characterization**	Compound purity	≥98% as determined by HPLC	Determined by high-performance liquid chromatography
**Small Molecule Compound Characterization**	Solvent for compound dissolution	Dimethyl sulfoxide (DMSO)	Common organic solvent
**Small Molecule Compound Characterization**	Final solvent concentration	≤0.5% (no solvent effect)	No solvent inhibition effect
**Reaction System & Conditions**	Reaction buffer system	50 mM Tris-HCl, pH 7.5	Standard enzyme reaction buffer
**Reaction System & Conditions**	Buffer additives	10 mM $MgCl_2$， 1 mM DTT	Essential for maintaining enzyme activity

**Reaction System & Conditions**	Total reaction volume	100 µL (96-well plate system)	Standard volume for microplate reader assays
**Reaction System & Conditions**	Reaction temperature	37°C (incubated at constant temperature)	Physiological temperature condition
**Reaction System & Conditions**	Enzyme-substrate incubation time	30 min	Optimal reaction time
**Reaction System & Conditions**	Substrate name and concentration	Specific fluorogenic substrate, 200 μM	Enzyme-specific substrate
**Instrument & Detection**	Microplate reader brand and model	Thermo Scientific Multiskan FC	Commercially available microplate reader
**Instrument & Detection**	Detection mode	End-point method (fluorescence detection)	Fluorescence end-point detection
**Instrument & Detection**	Detection wavelength	Excitation: 360 nm, Emission: 460 nm	Specific wavelengths for fluorescence detection
**Instrument & Detection**	Number of replicate wells per concentration	3 replicates (parallel measurements)	Ensures data reproducibility
**Data Quality Control & Analysis Characterization**	Blank control setup	Enzyme-substrate reaction system without compounds (100% enzyme activity baseline)	Baseline reference for enzyme activity
**Data Quality Control & Analysis Characterization**	Positive/negative control setup	Positive control (reference inhibitor with IC_{50} = 18.5 μM); Negative control (enzyme-free system)	Validates the effectiveness of the experimental system
**Data Quality Control & Analysis Characterization**	IC_{50} fitting method	Four-parameter logistic (4-PL) curve fitting	Standard method for enzyme inhibition fitting
**Data Quality Control & Analysis Characterization**	Outlier rejection criteria	Grubbs' test; values with $P < 0.05$ are rejected and retested	Ensures data reliability

6. Fitting data of 3a

Small molecule concentration (μM)	Relative Enzyme Activity (%)	Average relative enzyme activity (%)	Relative Enzyme Activity (%)	Notes
0	-∞	100	0.4	Blank control group (100% enzyme activity)
1	0	98.56	0.36	Slight inhibition
5	0.7	88.07	0.44	Mild inhibition
10	1	75.23	0.48	Moderate inhibition
20	1.3	50.76	0.5	Near 50% inhibition (around IC_{50})
50	1.7	26.55	0.33	High-level inhibition
100	2	11.02	0.28	Near-complete inhibition
200	2.3	4.28	0.16	Complete inhibition

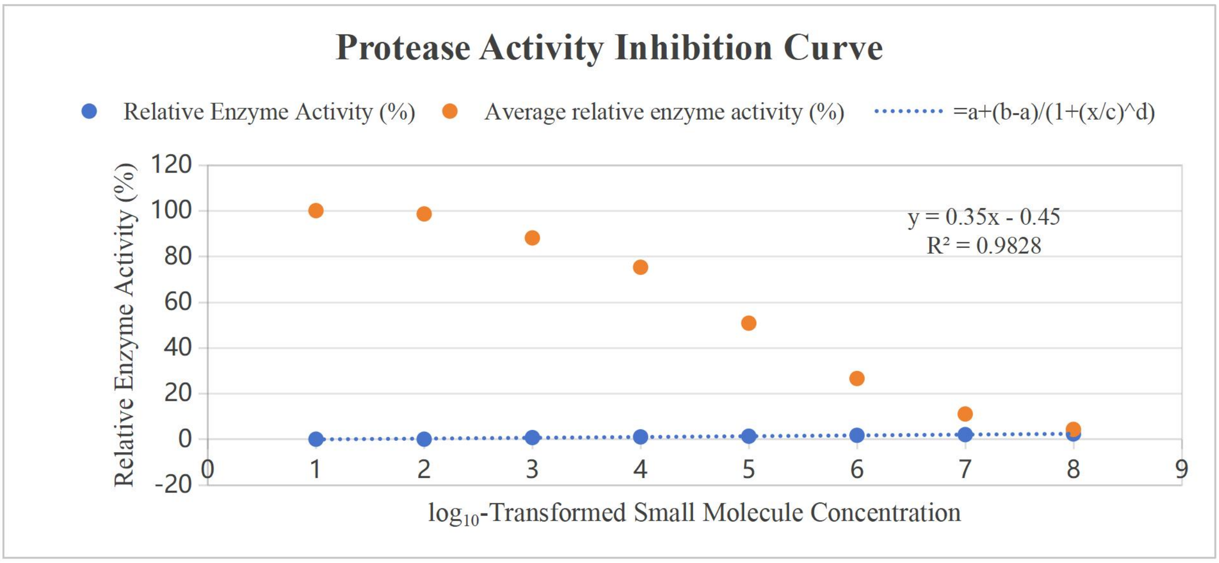

Fitting parameter	Value	Acceptance criteria	Validity
IC_{50} value	20.10 μM	-	Valid
95% confidence interval (CI)	18.50 ~ 22.00 μM	Narrower intervals indicate higher reliability	Valid
Coefficient of determination (R^2)	0.988	≥0.95	Valid
Hill slope	1.25	Normal range: 0.5–2	Normal
Enzyme activity at full inhibition	0.04	Lower values indicate stronger inhibition	Good
Enzyme activity of the blank control	1	≈100	Normal

Core conclusion: The half-maximal inhibitory concentration (IC_{50}) of the unknown small molecule against the target protease was determined to be 20.10 μM (95% CI: 18.50–22.00 μM). The fitted dose-response curve exhibited a typical sigmoidal profile, indicating good data reproducibility and high validity.

7. Fitting data of 3b

Small molecule concentration (μM)	Relative Enzyme Activity (%)	Average relative enzyme activity (%)	Standard deviation (SD) of relative enzyme activity (%)	Notes
0	-∞	100	0.38	Blank control (100% enzyme activity)
2	0.3	97.25	0.32	Slight inhibition
8	0.9	85.18	0.42	Mild inhibition
15	1.18	68.36	0.47	Moderate inhibition
30	1.48	49.82	0.53	Near 50% inhibition (around IC_{50})
60	1.78	23.15	0.3	Strong inhibition
120	2.08	9.64	0.27	Near-complete inhibition
250	2.4	3.86	0.14	Complete inhibition

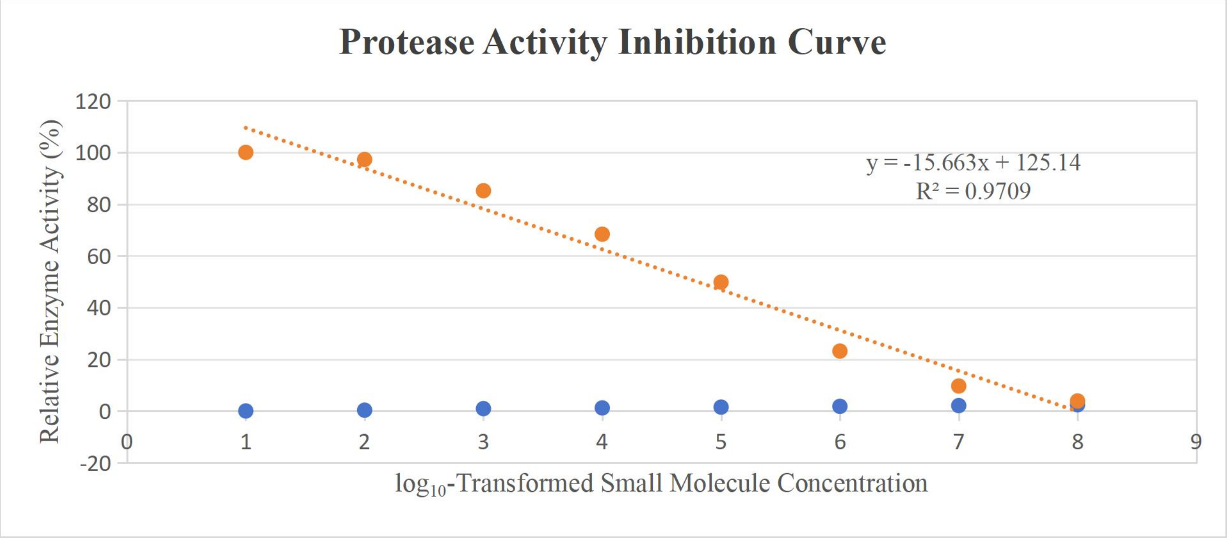

Fitting parameter	Results of the novel small molecule	Experimental requirements	Compliance status
IC_{50} value	29.75 μM	-	Compliant
95% confidence interval (CI)	27.62 ~ 32.08 μM	Output interval to ensure reliability	Compliant
Coefficient of determination (R^2)	0.991	≥0.95	Compliant
Hill slope	1.31	Normal range: 0.5–2	Normal
Enzyme activity at full inhibition	0.0386	Achieved full inhibition	Compliant

Enzyme activity of the blank control	1	100% baseline at 0 concentration	Compliant
Maximum SD of replicate wells	0.0053	Low variability is acceptable	Compliant

8. ^{1}H and ^{13}C NMR spectra of all the products

3-((2-methoxy-2-phenyl)ethylthio)-2-phenylimidazo[1,2-*a*]pyridine (3a)

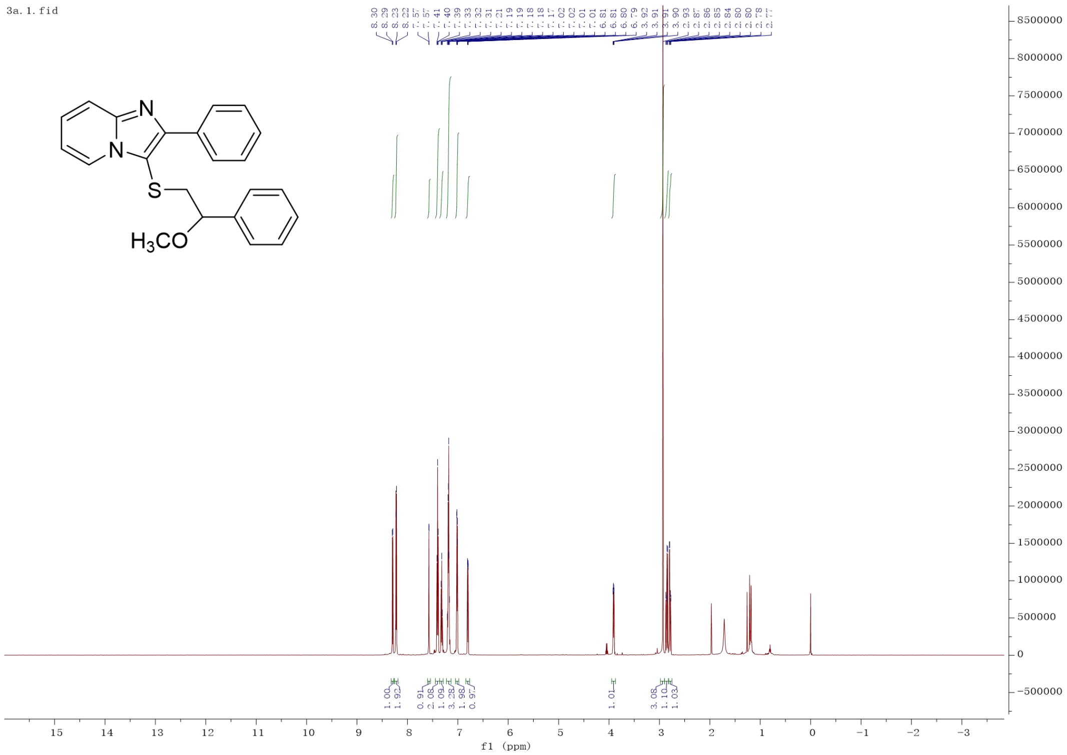

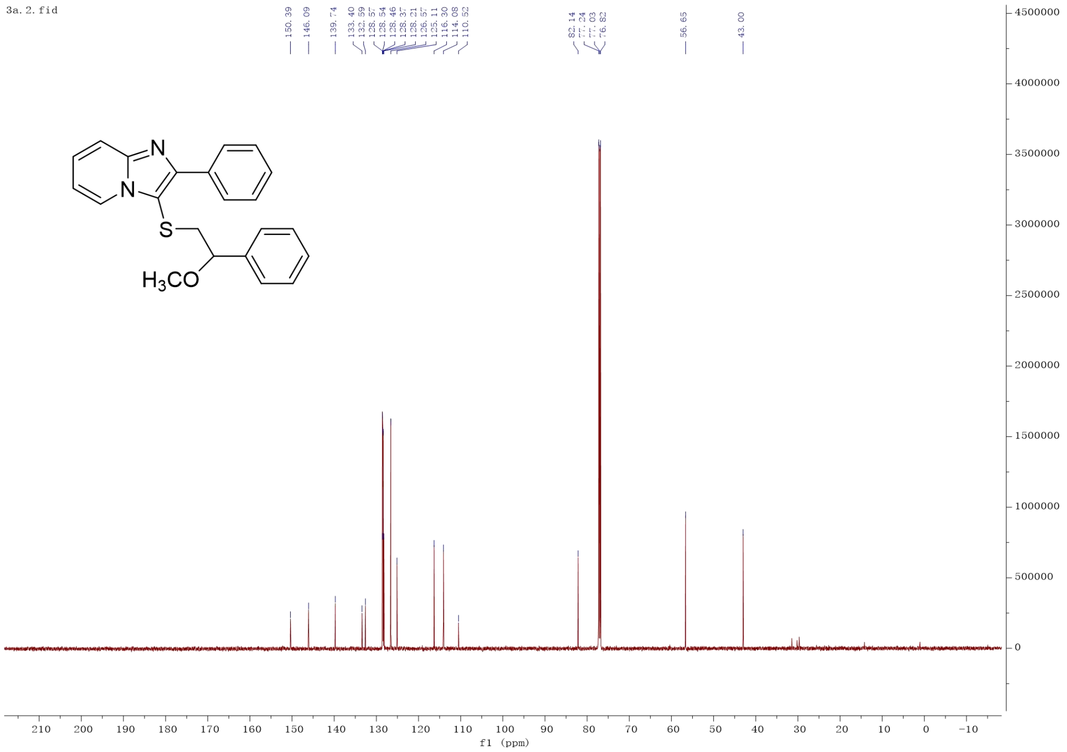

3-((2-methoxy-2-phenylethyl)thio)-2-(4-methylphenyl)imidazo[1,2-*a*]pyridine (3b)

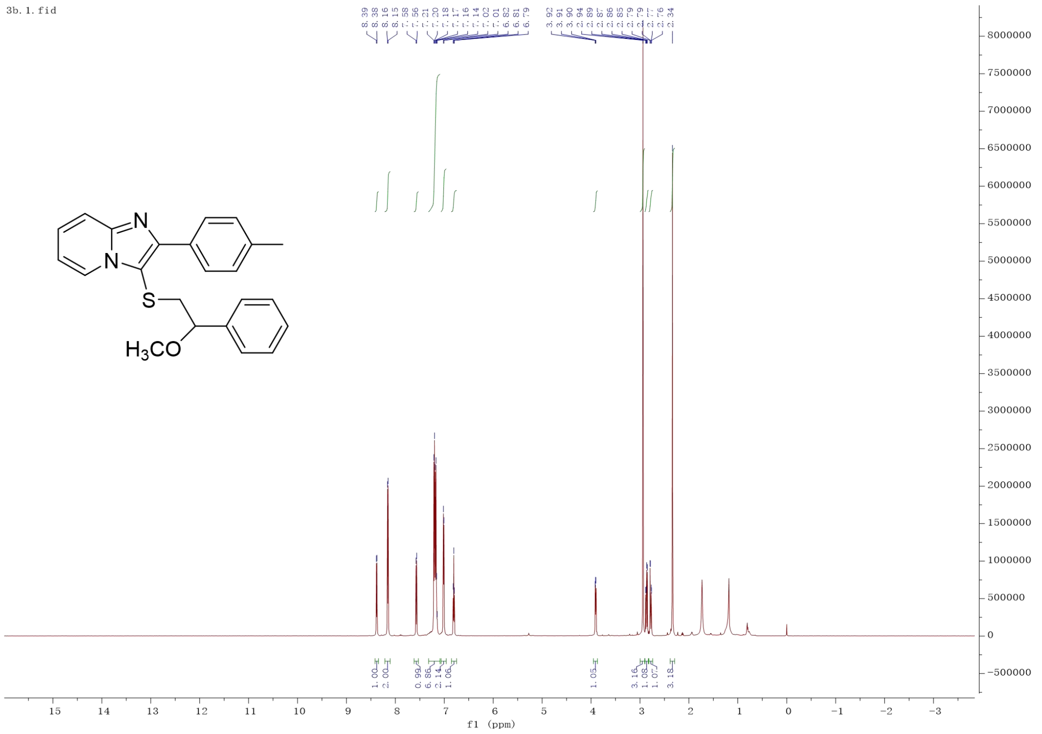

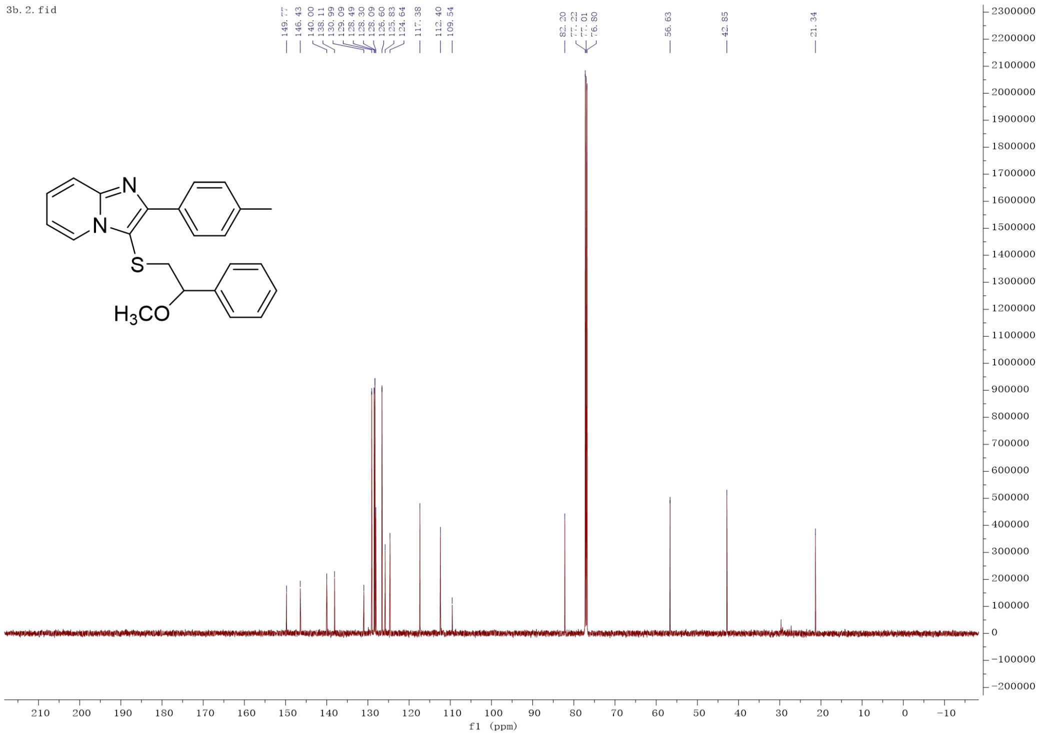

2-(4-chlorophenyl)-3-((2-methoxy-2-phenyl)ethylthio)imidazo[1,2-*a*]pyridine (3c)

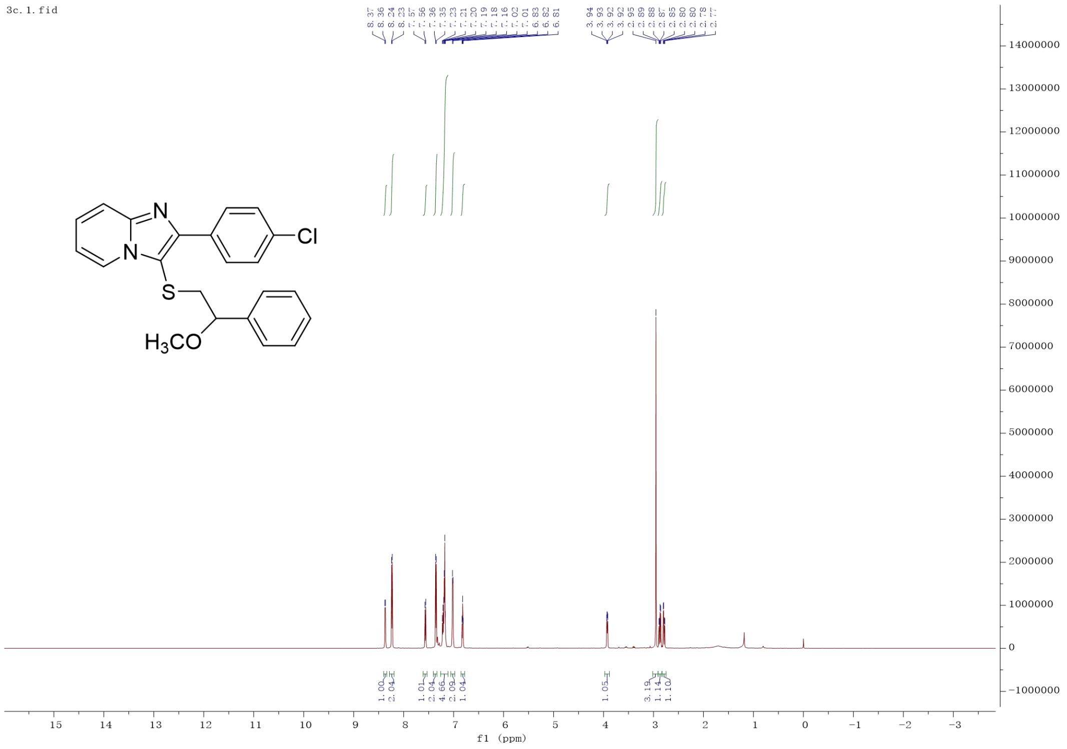

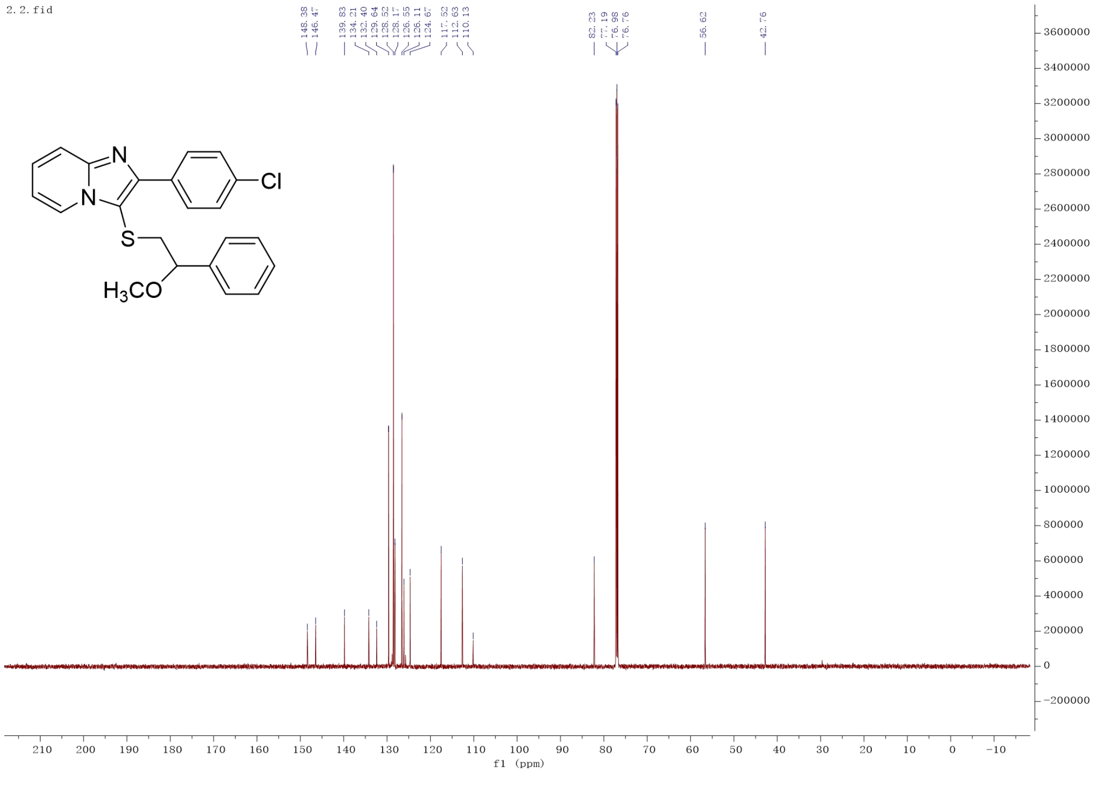

2-(4-methoxyphenyl)-3-((2-methoxy-2-phenyl)ethylthio)imidazo[1,2-*a*]pyridine (3d)

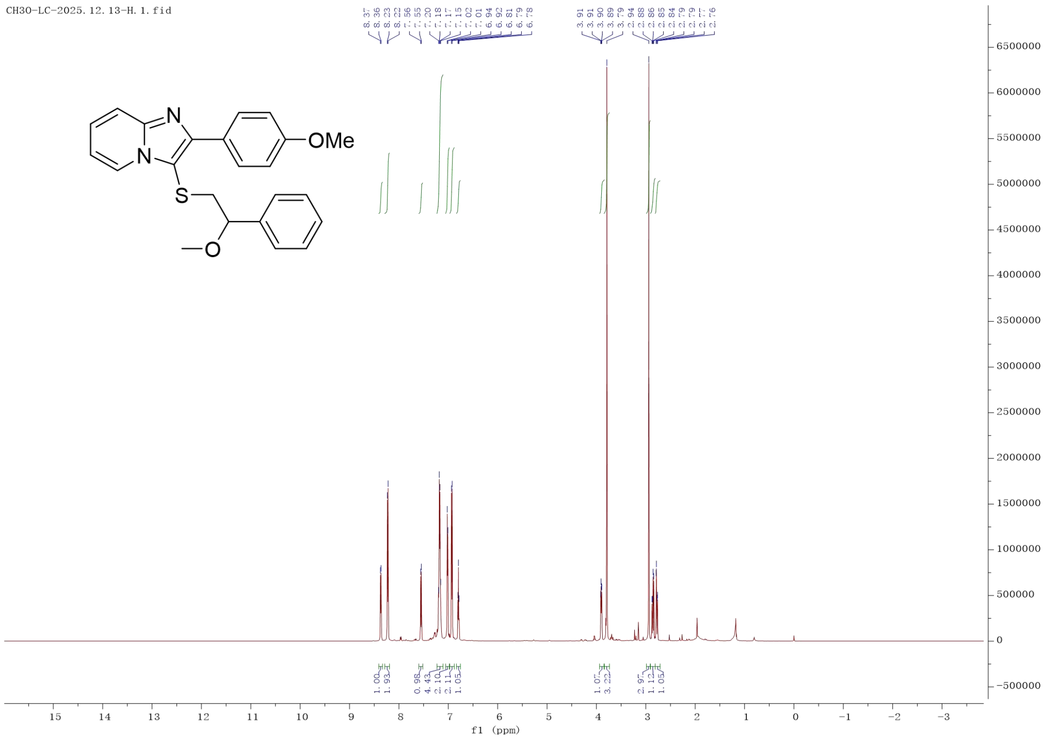

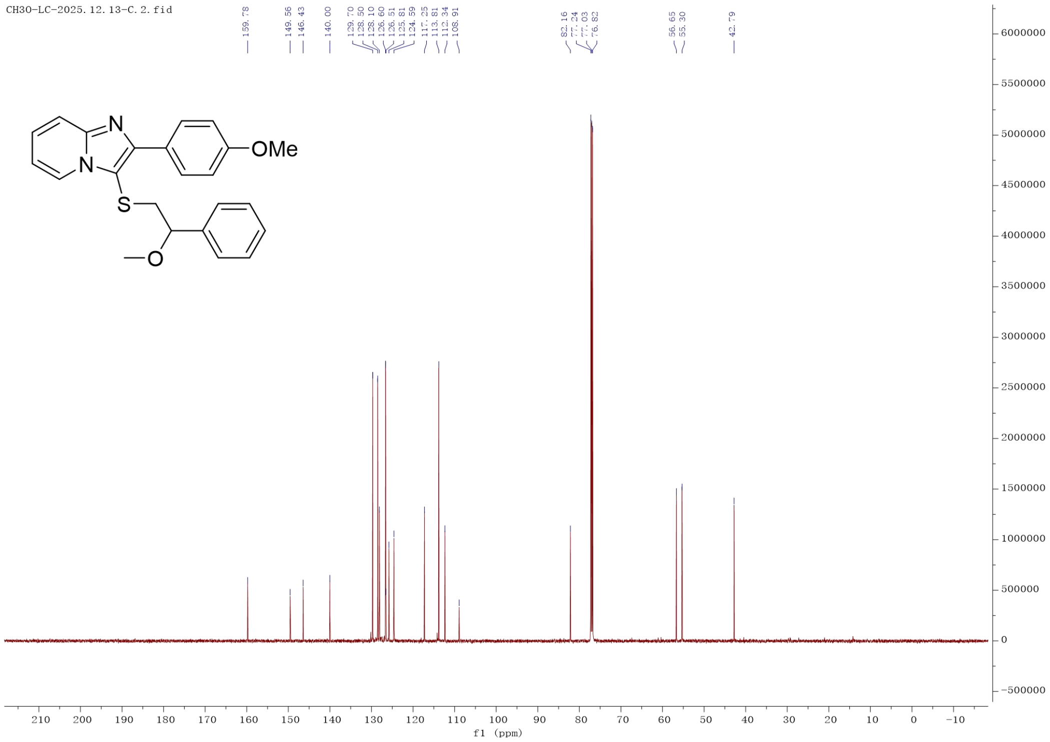

3-((2-ethoxy-2-phenylethyl)thio)-2-phenylimidazo[1,2-*a*]pyridine (3f)

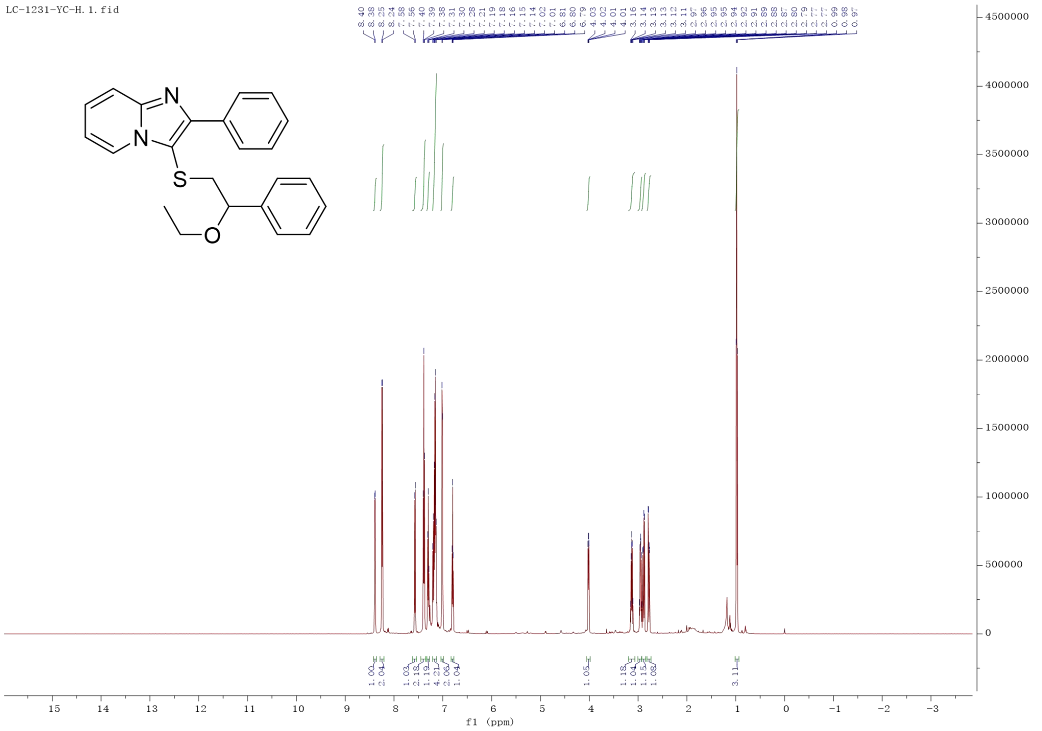

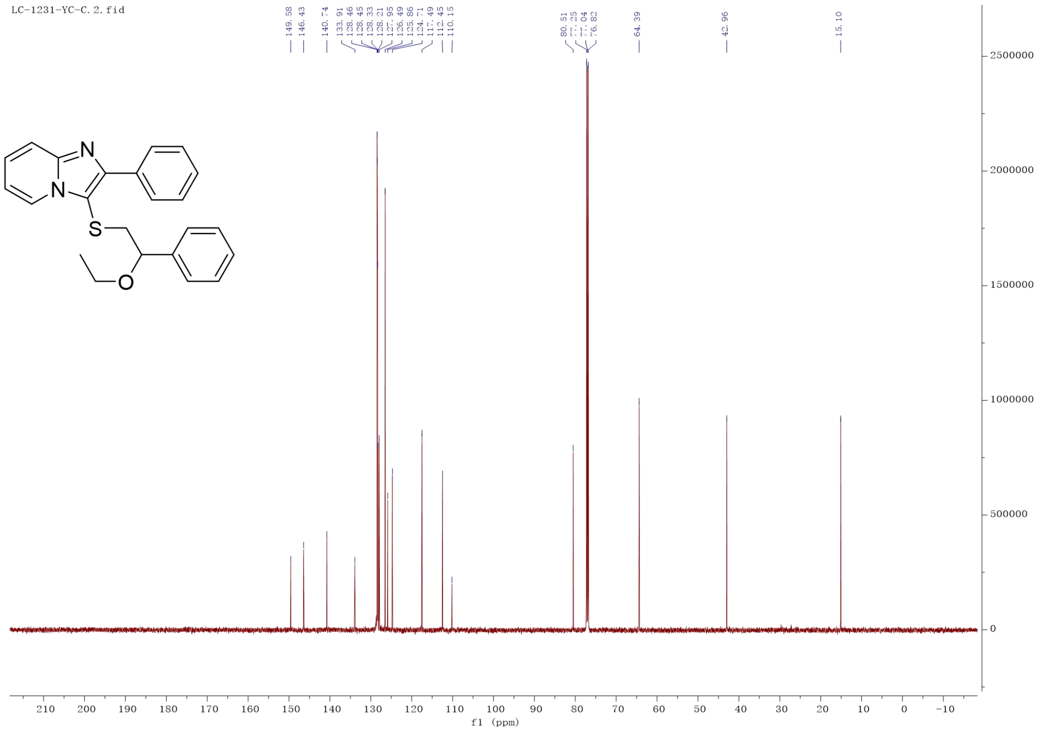

3-((2-ethoxy-2-phenylethyl)thio)-2-(4-methylphenyl)imidazo[1,2-*a*]pyridine (3g)

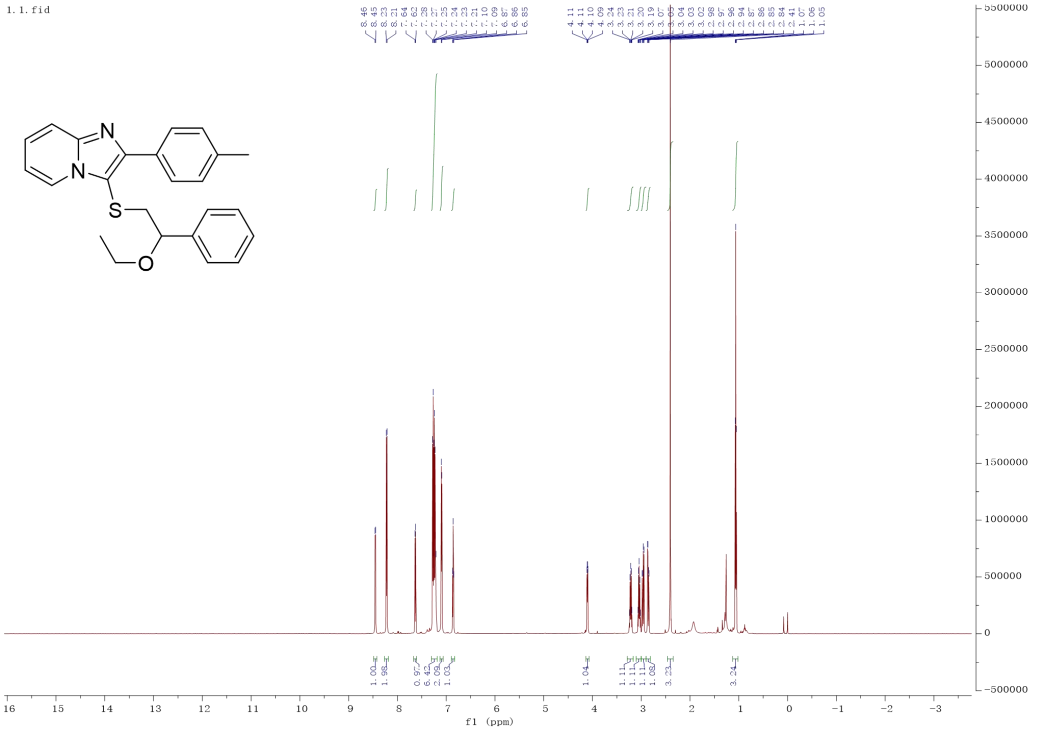

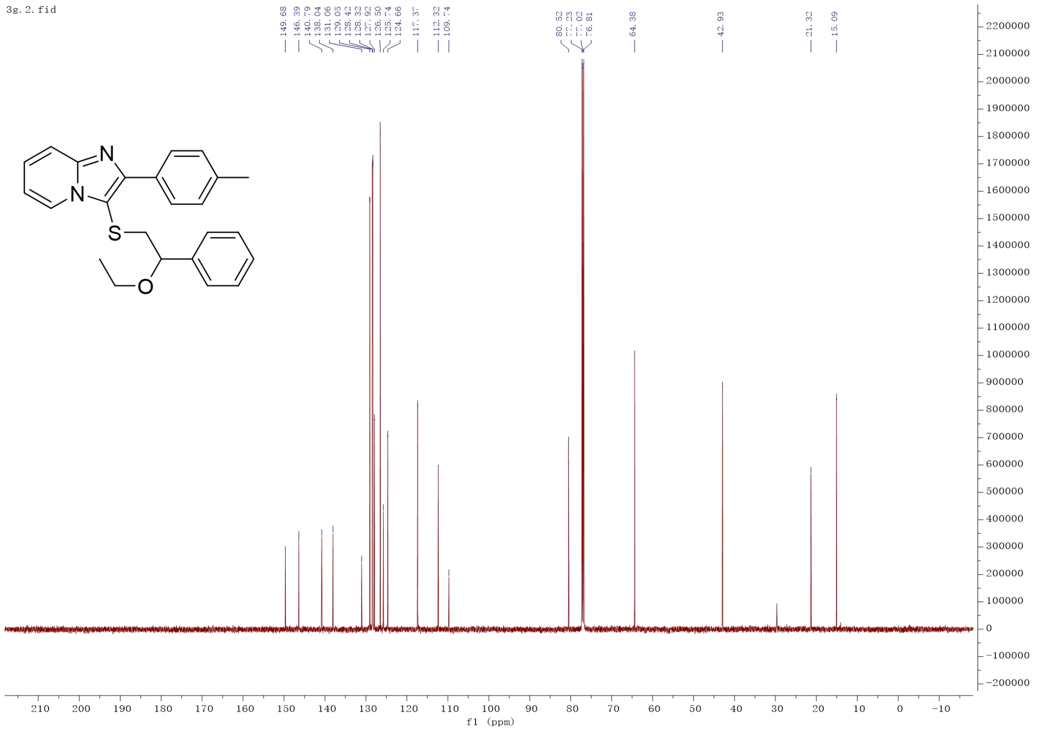

2-(4-chlorophenyl)-3-((2-ethoxy-2-phenylethyl)thio)imidazo[1,2-*a*]pyridine (3h)

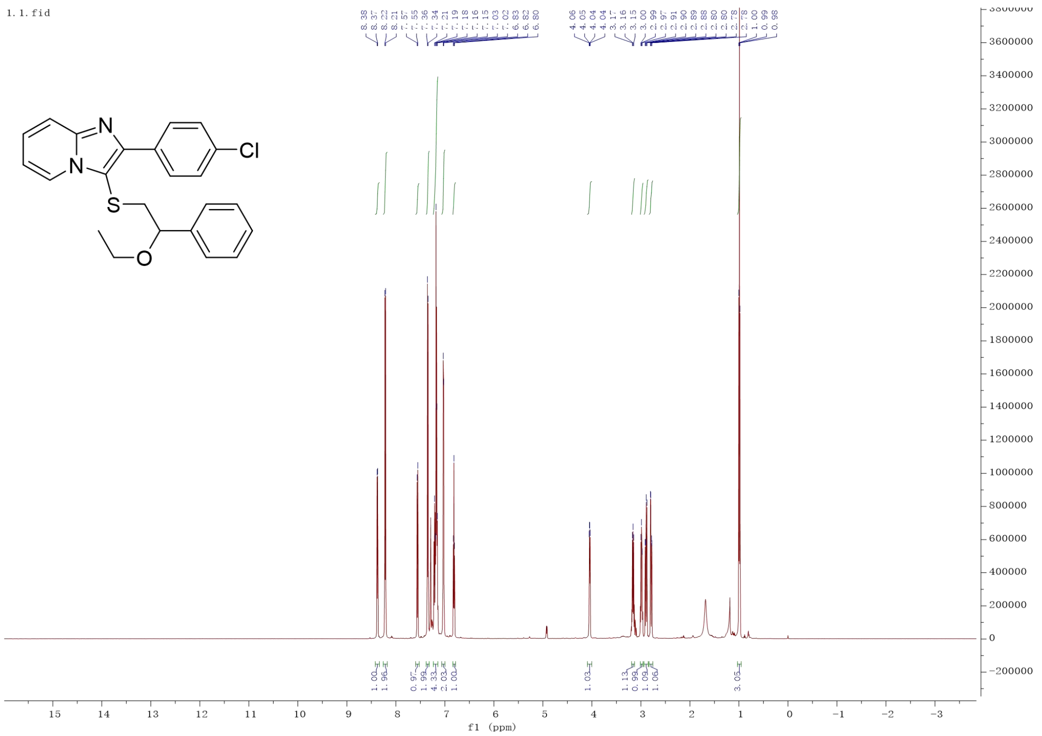

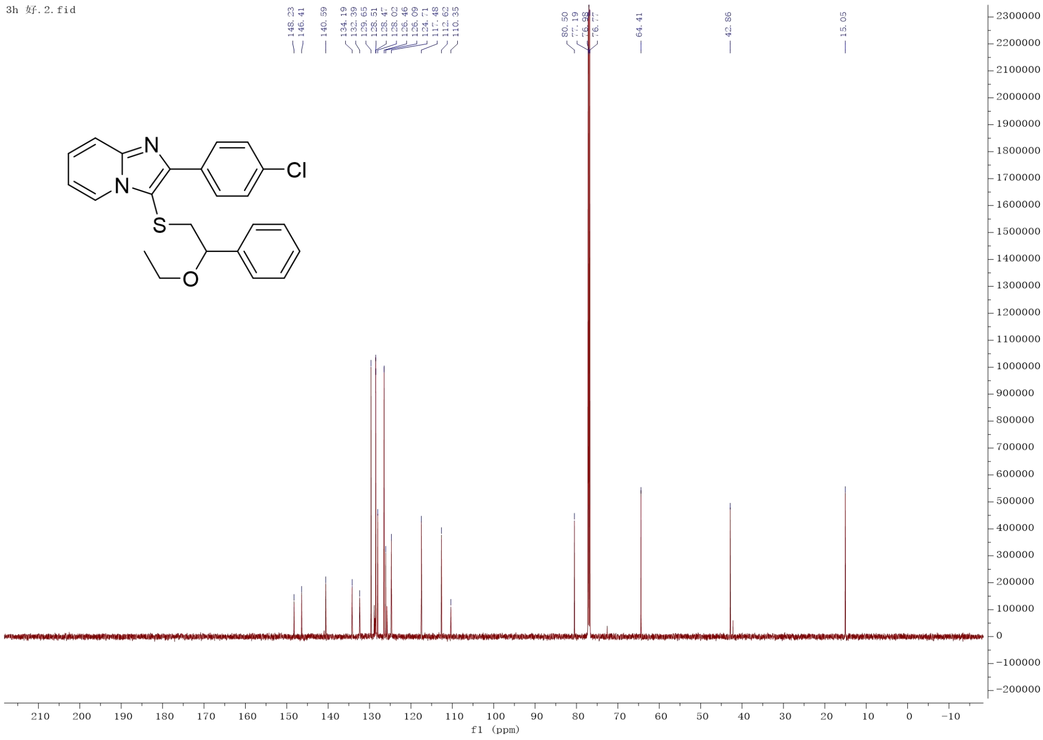

2-phenyl-3-((2-phenyl-2-propoxy)ethylthio)imidazo[1,2-*a*]pyridine (3i)

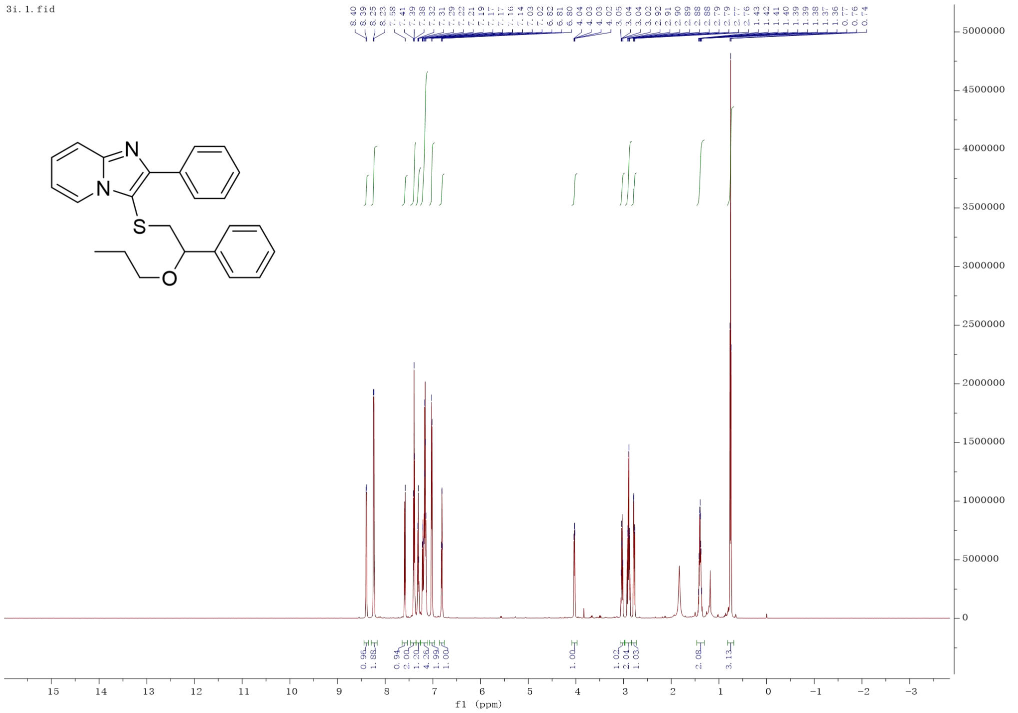

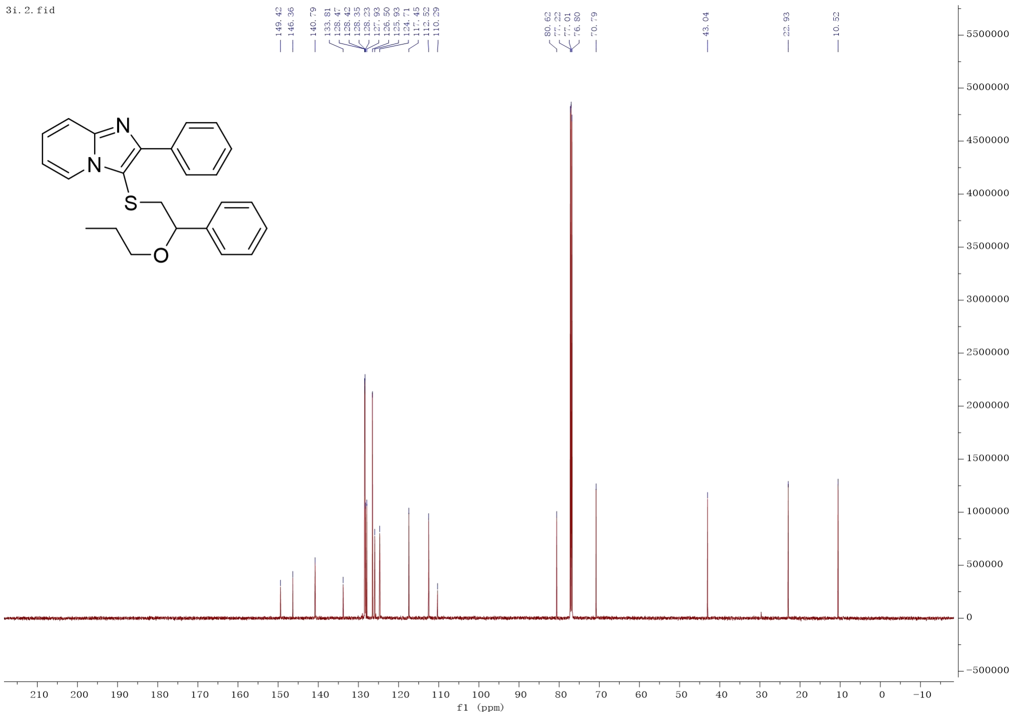

2-(4-methylphenyl)-3-((2-phenyl-2-propoxy)ethylthio)imidazo[1,2-*a*]pyridine (3j)

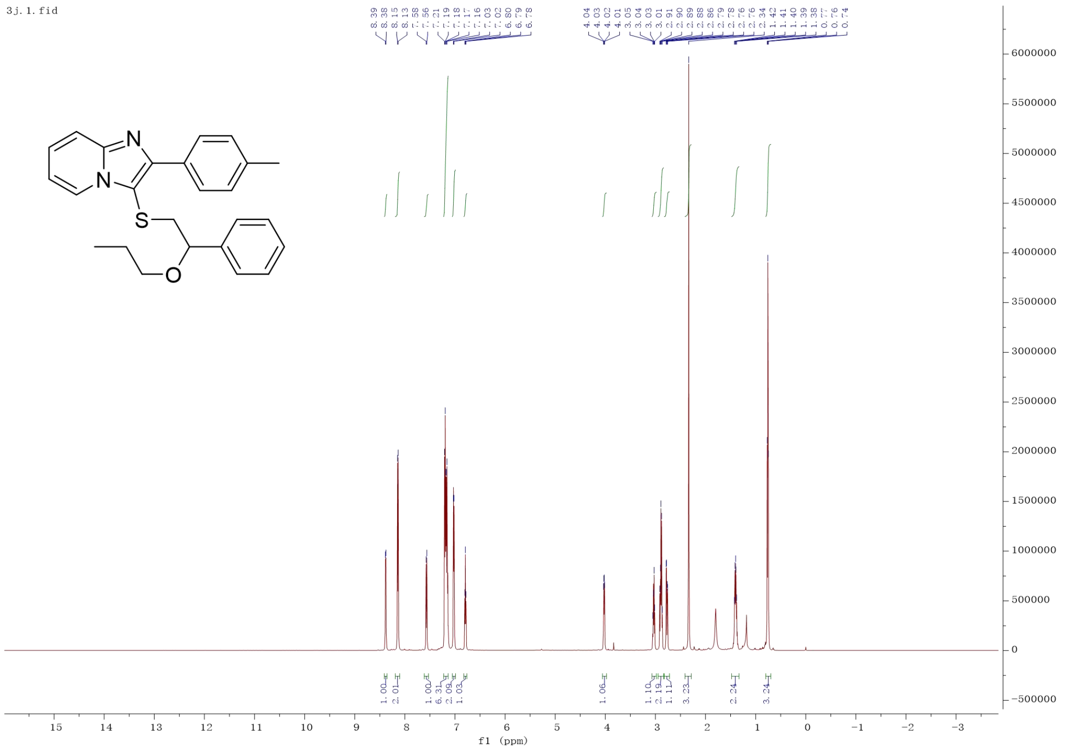

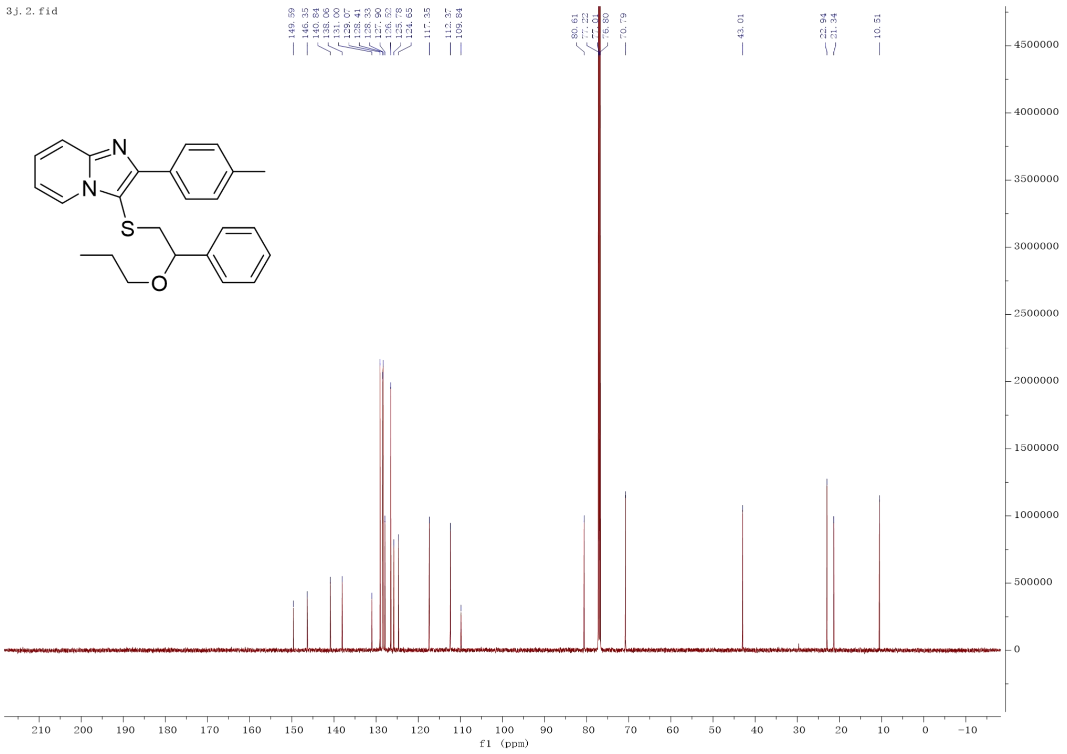

2-(4-chlorophenyl)-3-((2-phenyl-2-propoxy)ethylthio)imidazo[1,2-*a*]pyridine (3k)

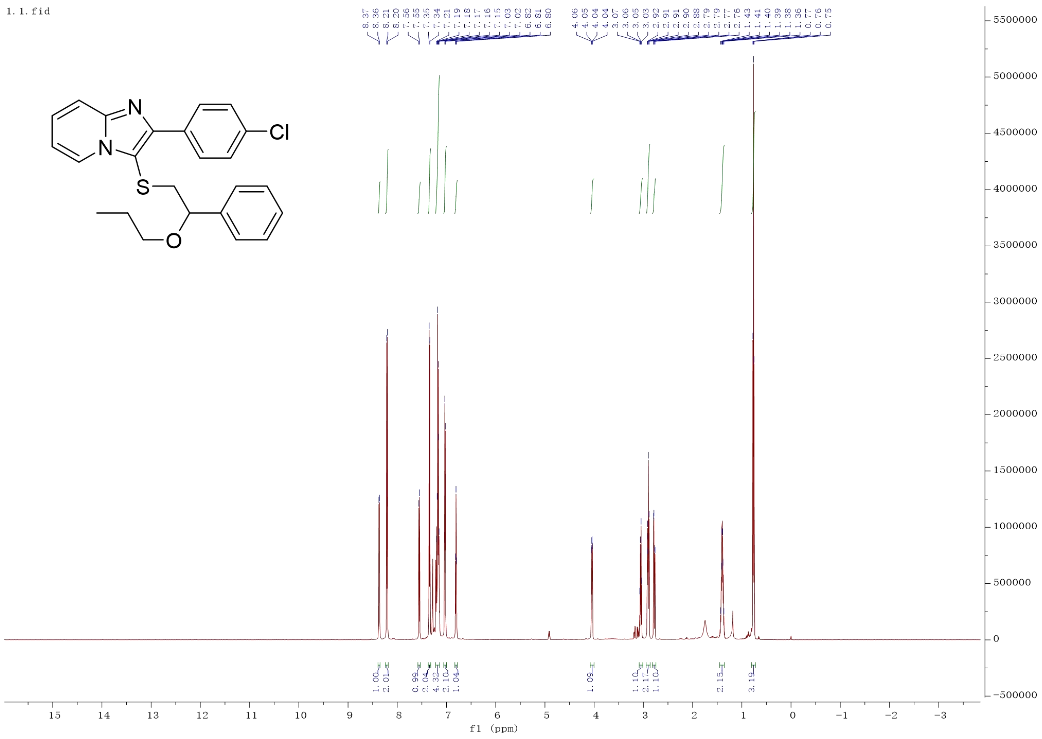

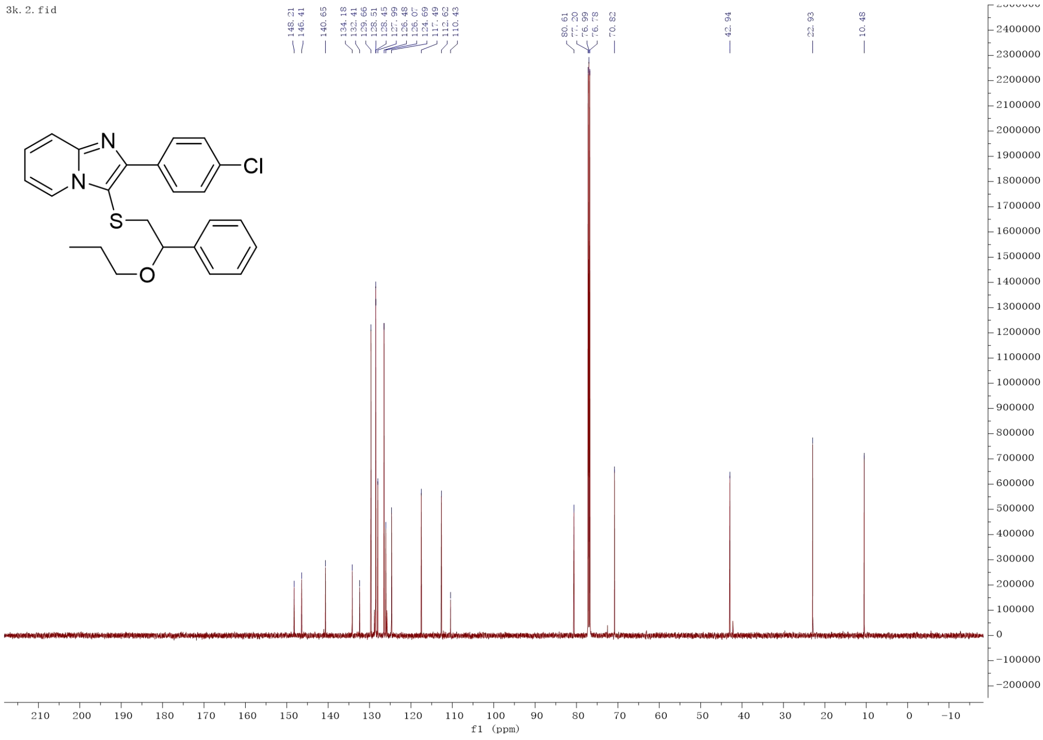

3-((2-isopropoxy-2-phenyl)ethylthio)-2-phenylimidazo[1,2-*a*]pyridine (3l)

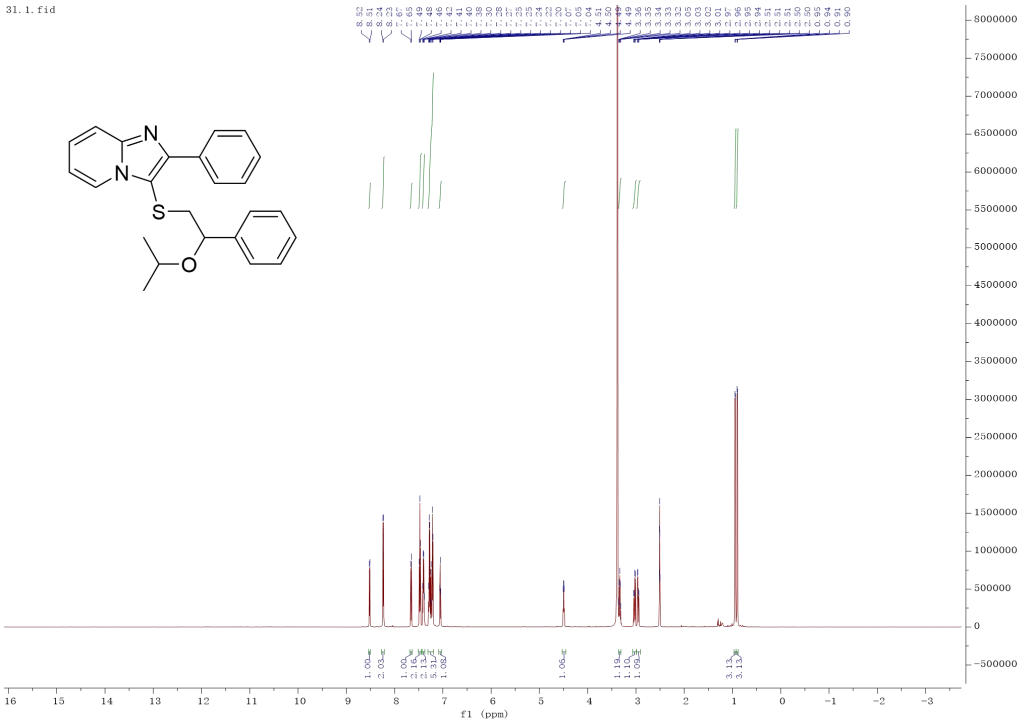

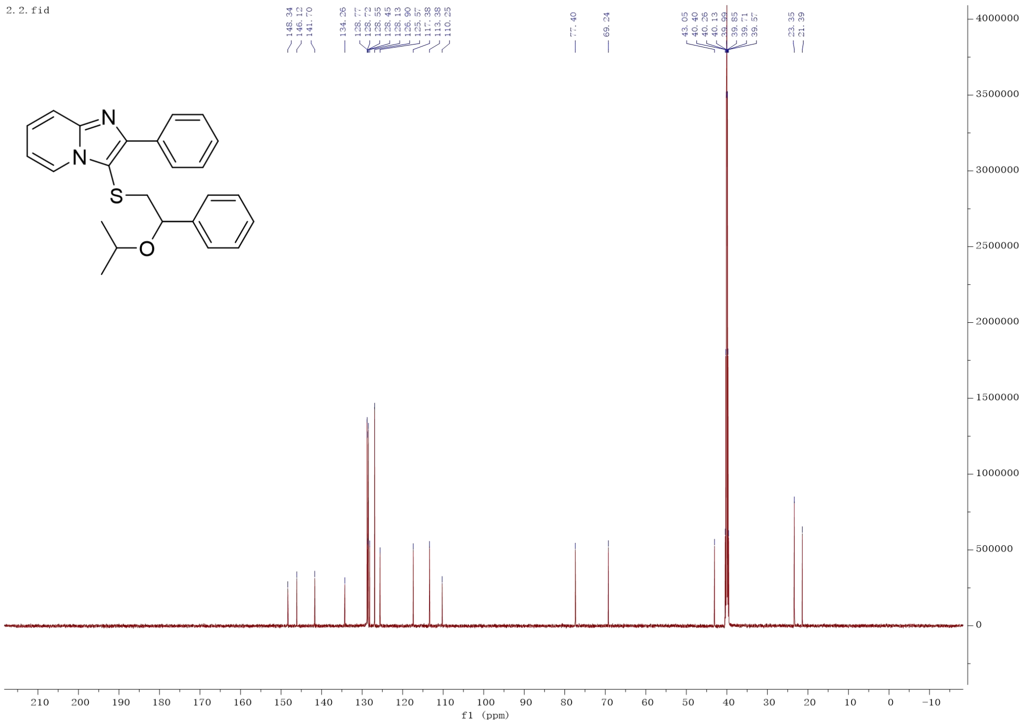

3-((2-isopropoxy-2-phenyl)ethylthio)-2-(4-methylphenyl)imidazo[1,2-*a*]pyridine (3m)

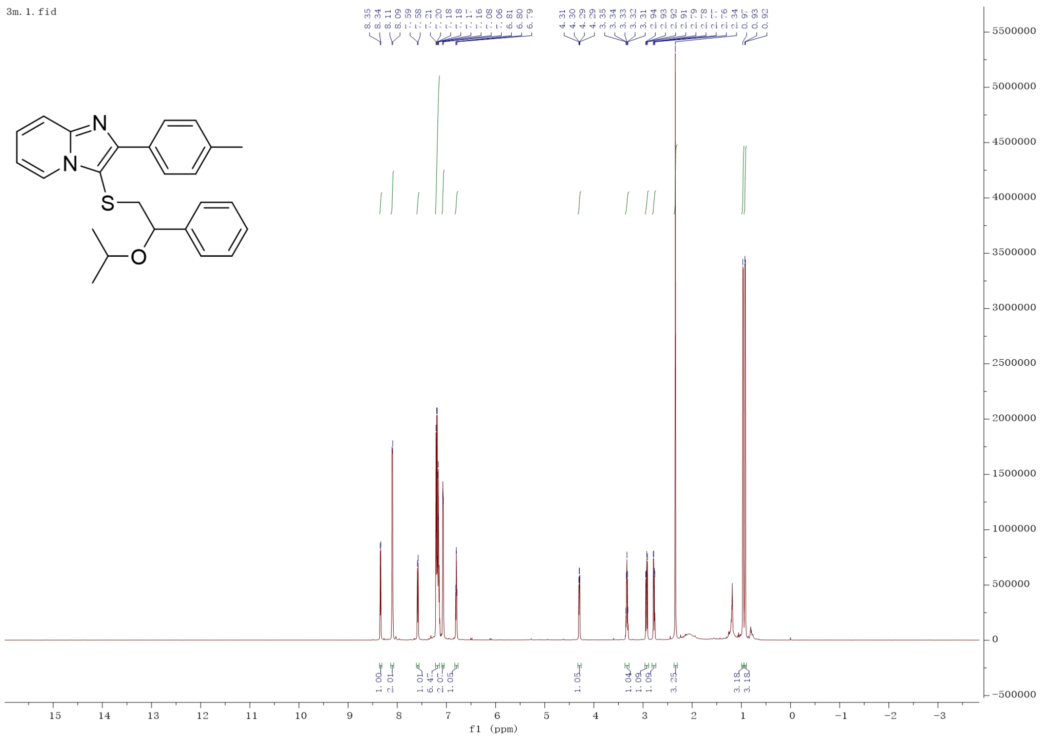

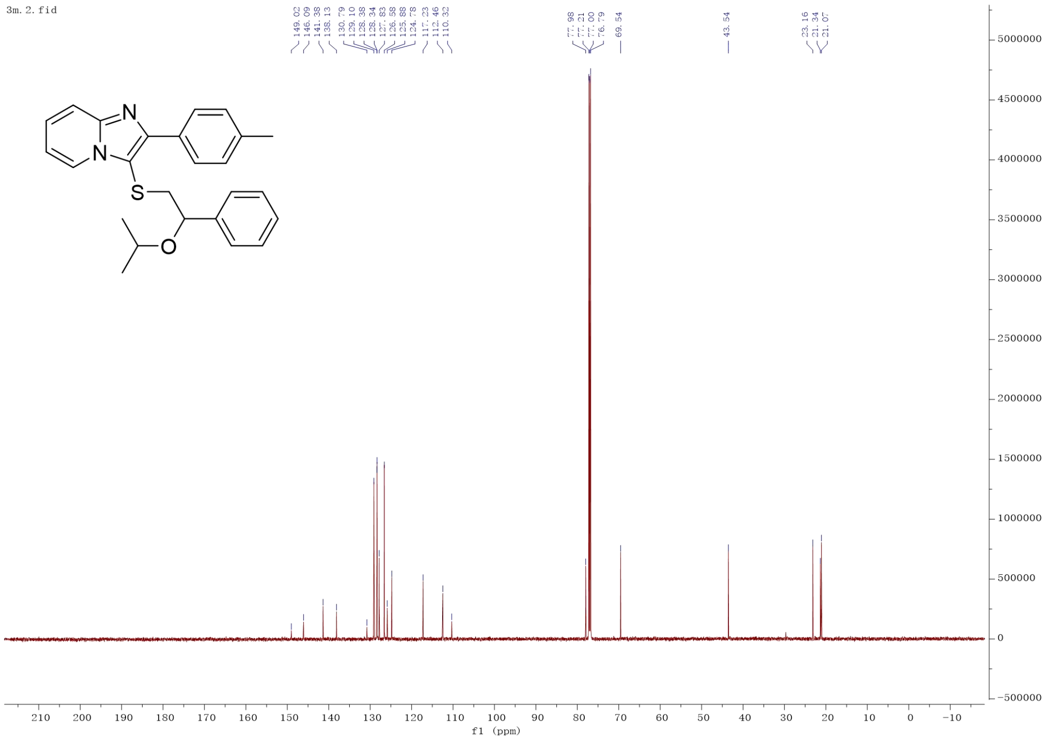

2-(4-chlorophenyl)-3-((2-isopropoxy-2-phenyl)ethylthio)imidazo[1,2-*a*]pyridine (3n)

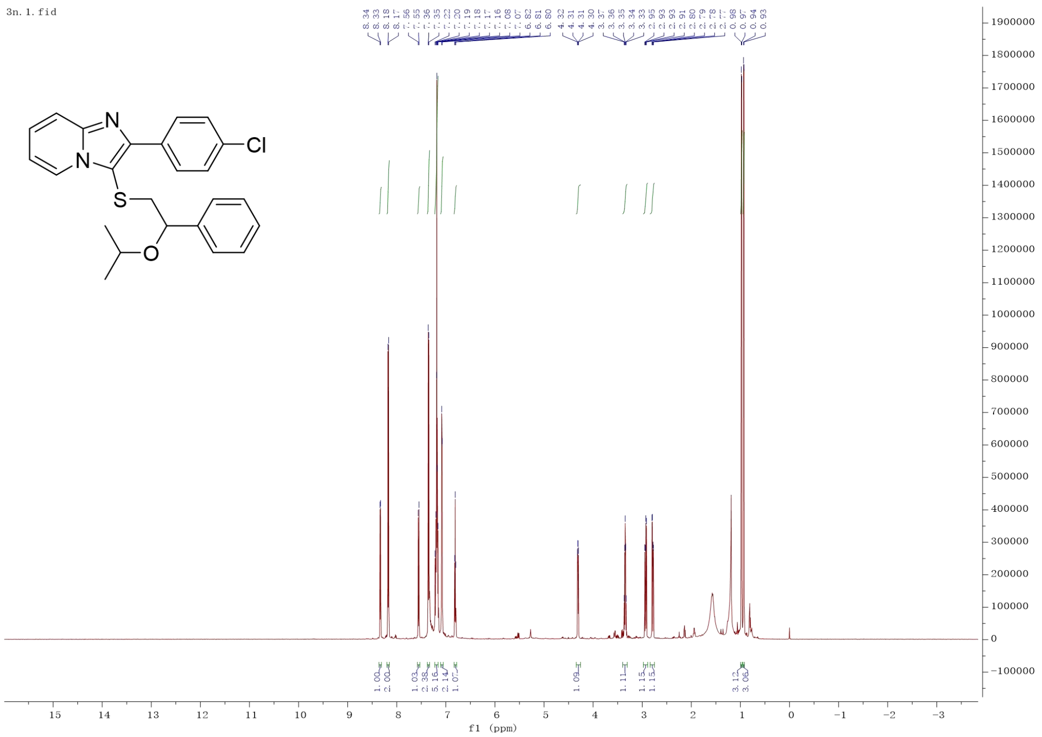

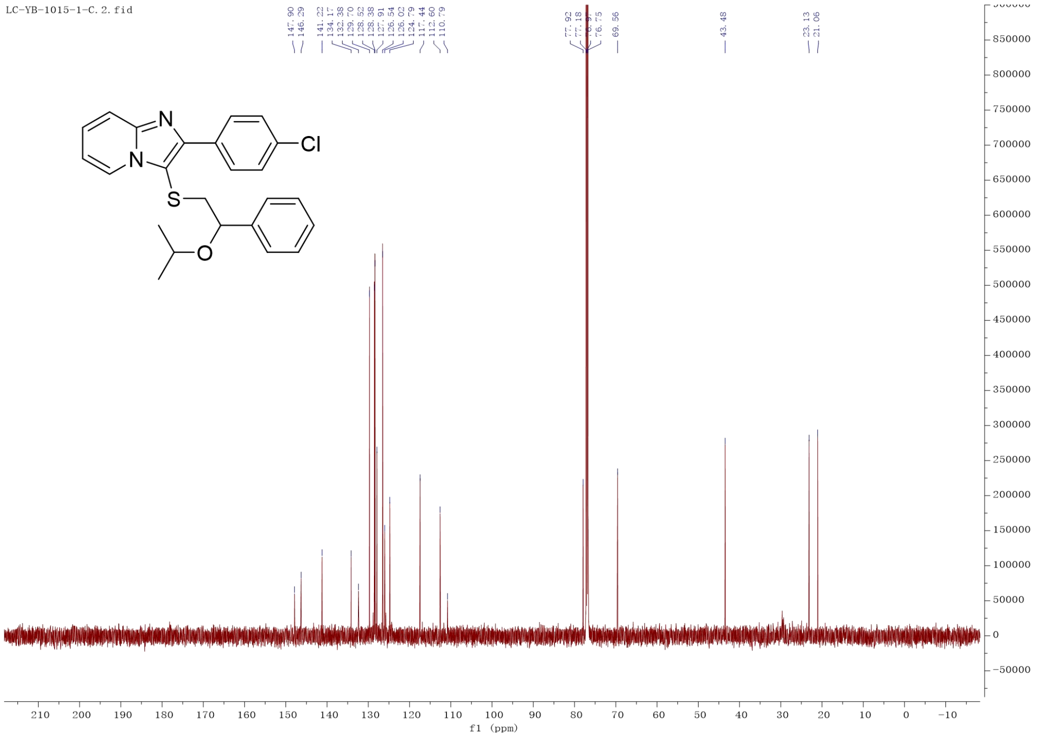

3-((2-butoxy-2-phenyl)ethylthio)-2-phenylimidazo[1,2-*a*]pyridine (3o)

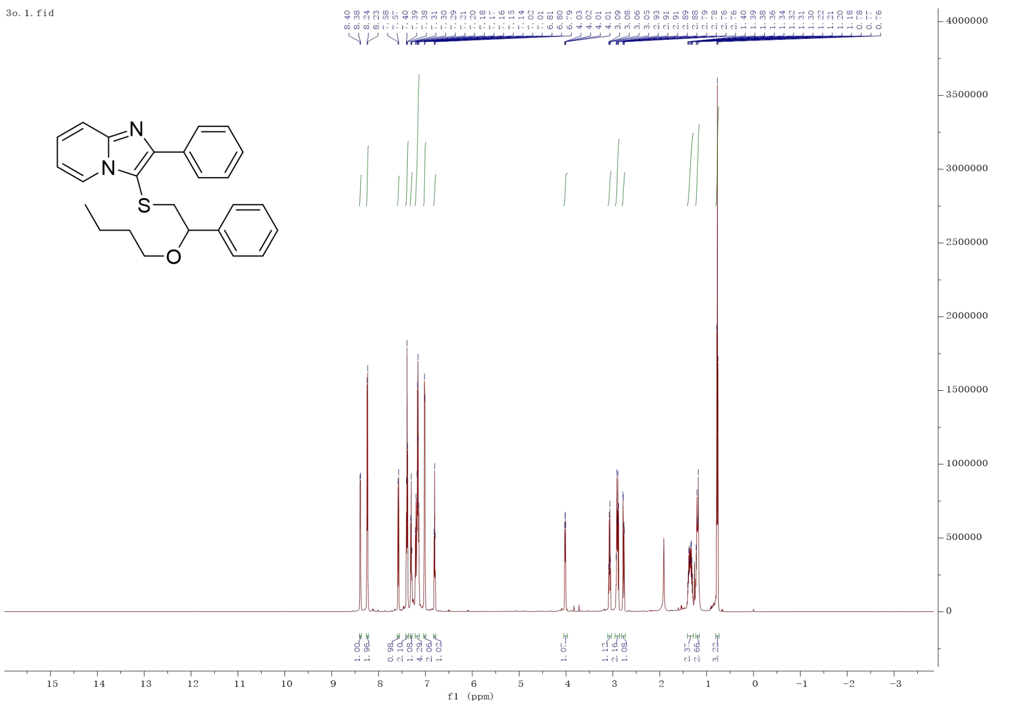

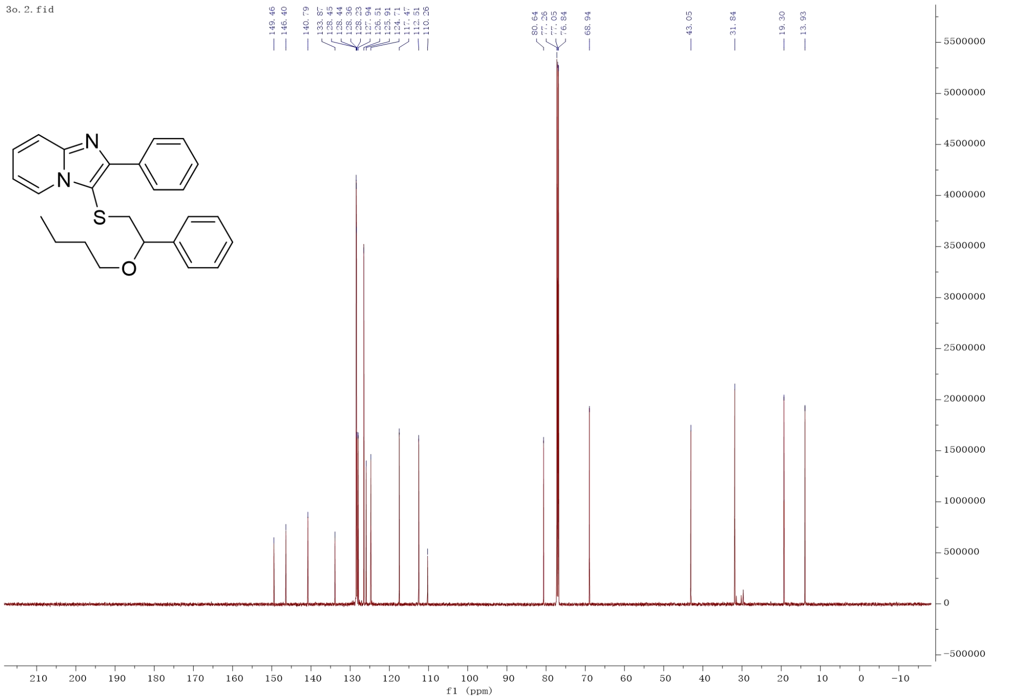

3-((2-butoxy-2-phenyl)ethylthio)-2-(4-methylphenyl)imidazo[1,2-*a*]pyridine (3p)

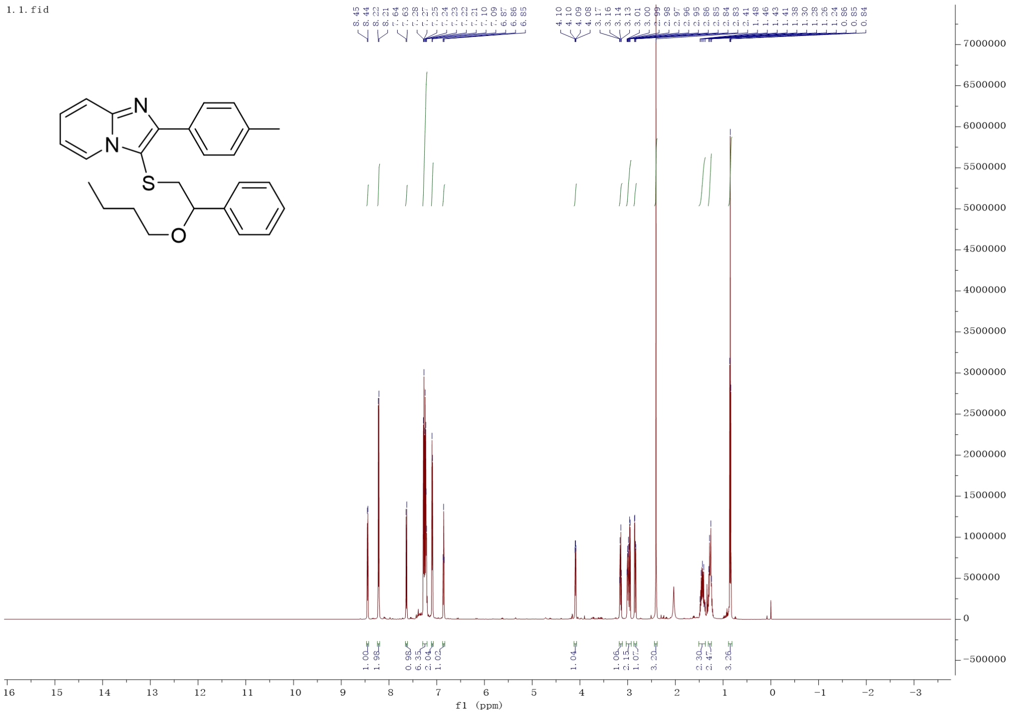

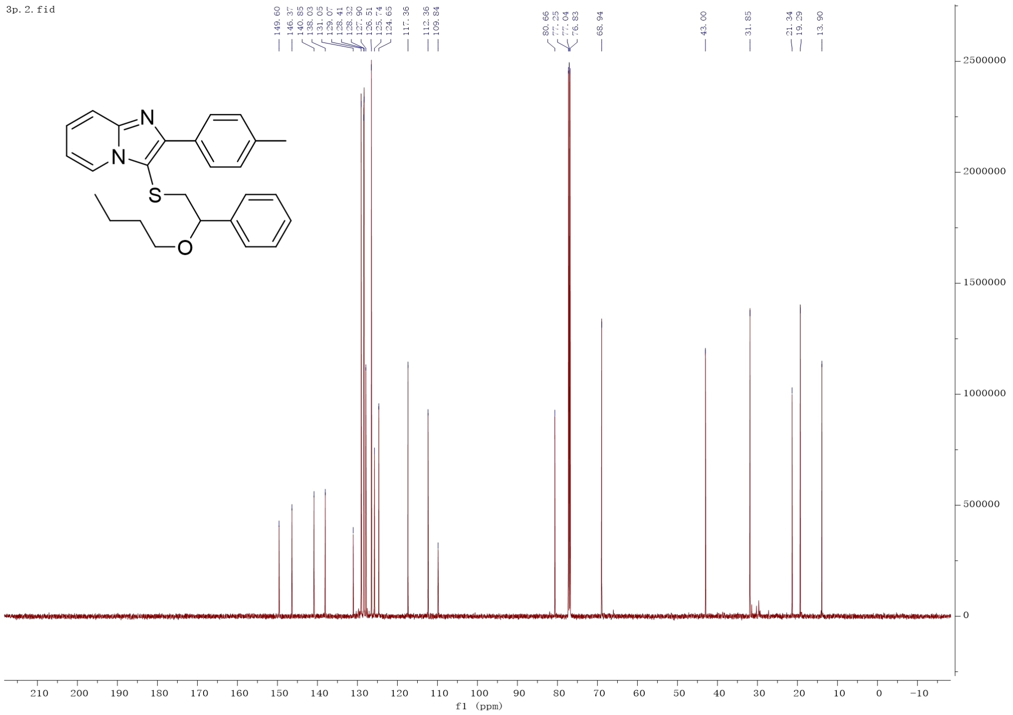

3-((2-butoxy-2-phenyl)ethylthio)-2-(4-chlorophenyl)imidazo[1,2-*a*]pyridine (3q)

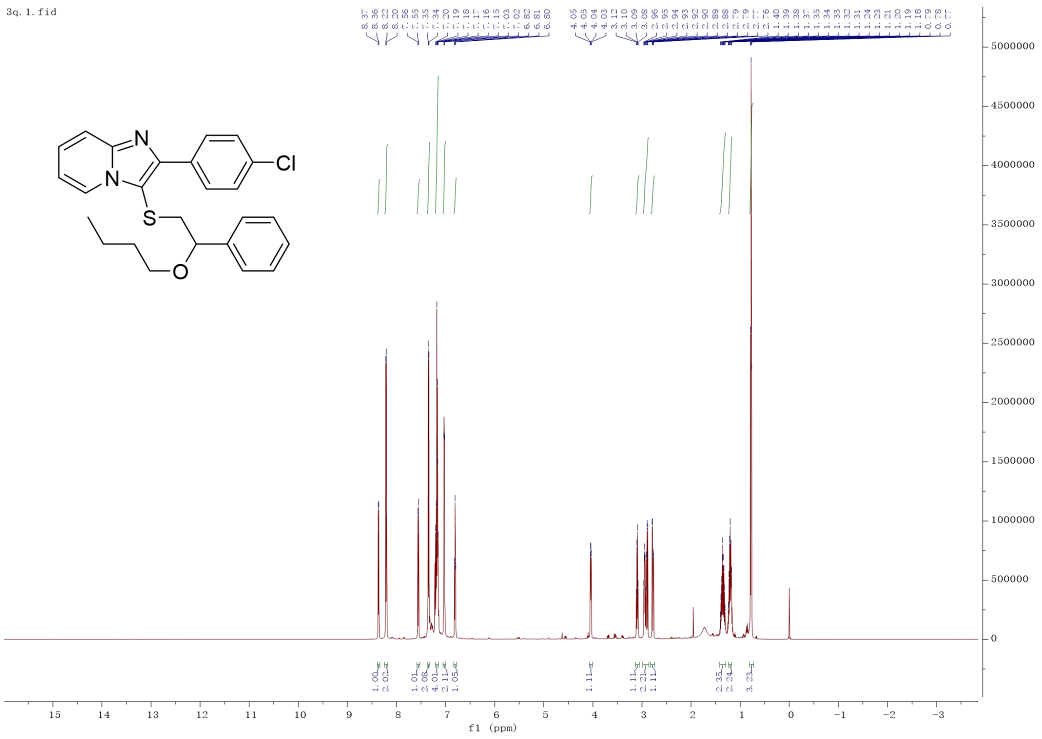

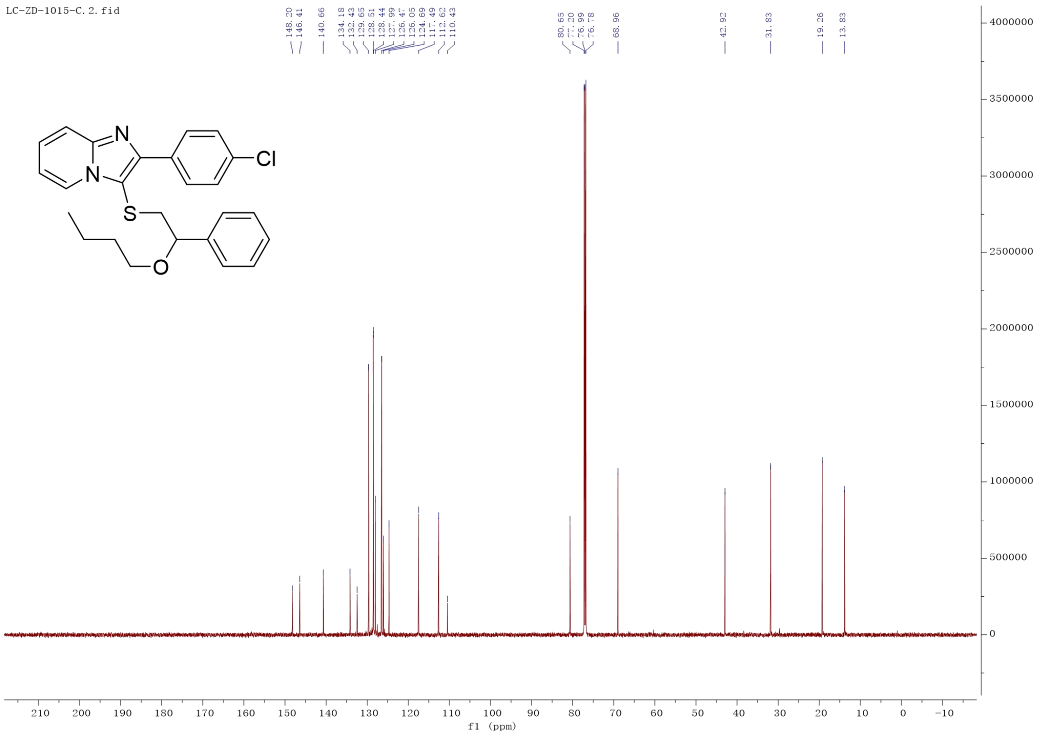

3-((2-methoxy-2-phenyl)ethylthio)-8-methyl-2-phenylimidazo[1,2-*a*]pyridine (3s)

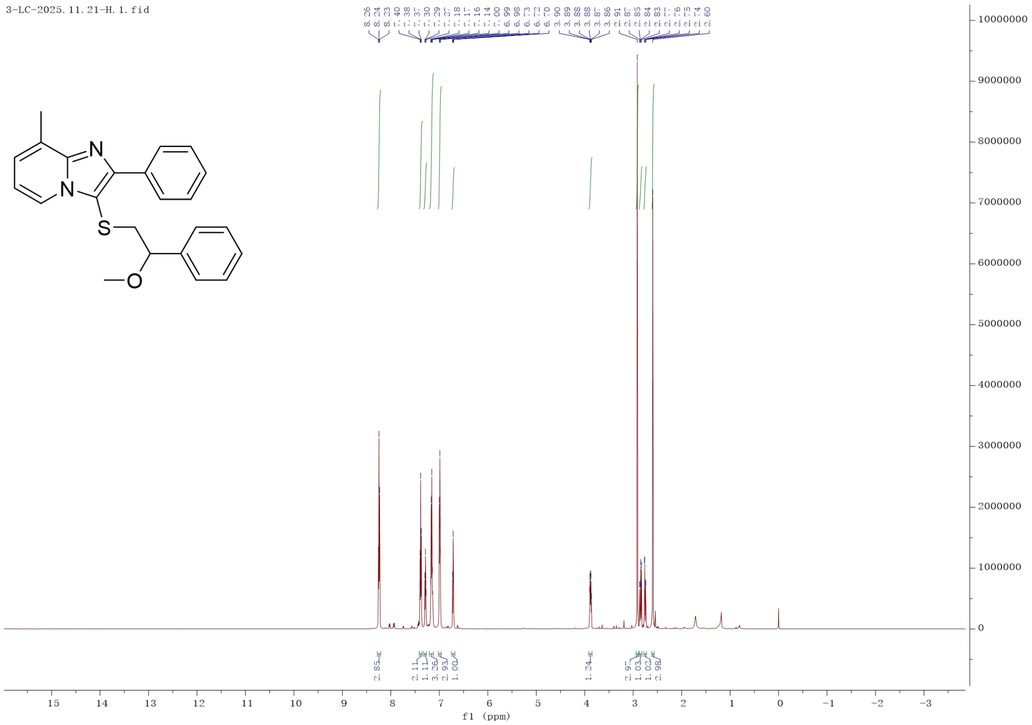

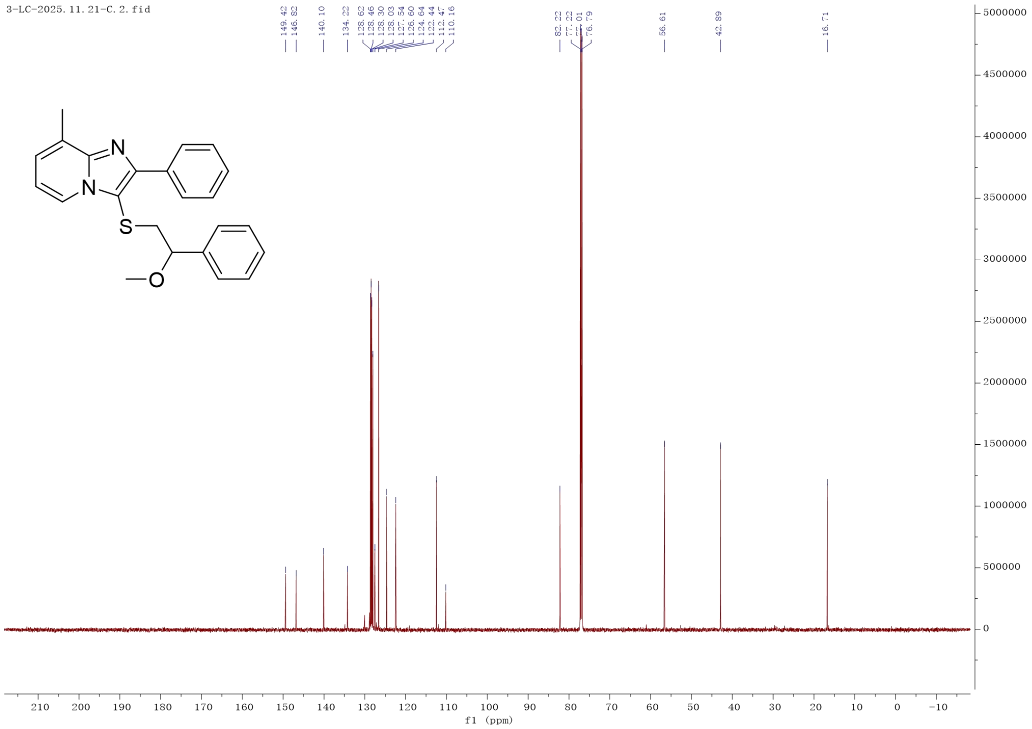

3-((2-methoxy-2-phenyl)ethylthio)-7-methyl-2-phenylimidazo[1,2-*a*]pyridine (3t)

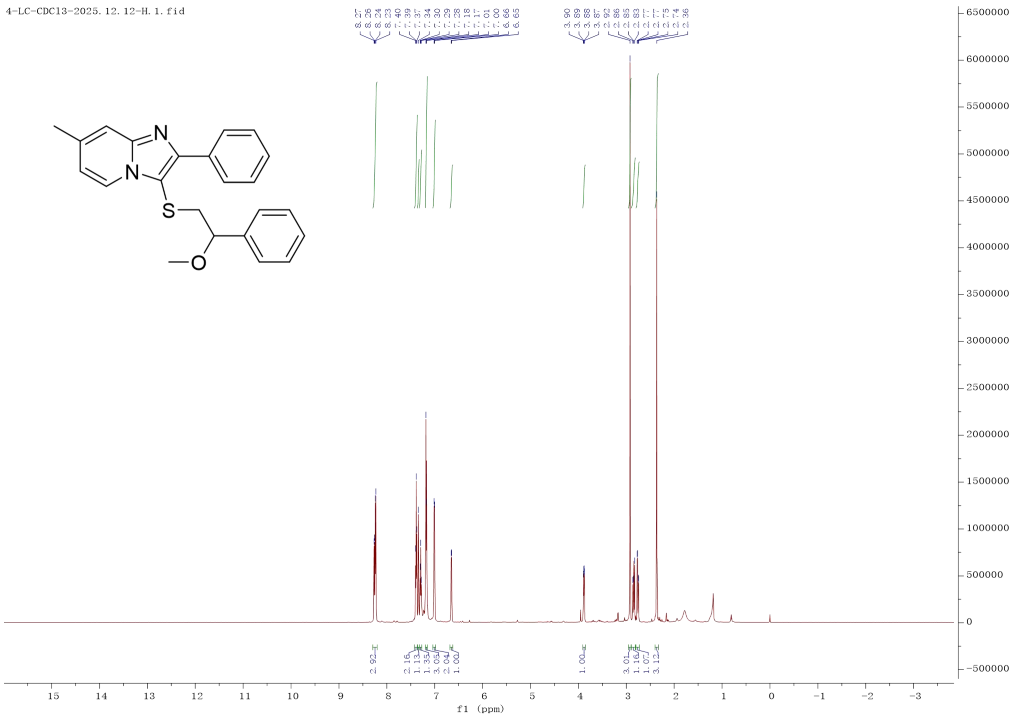

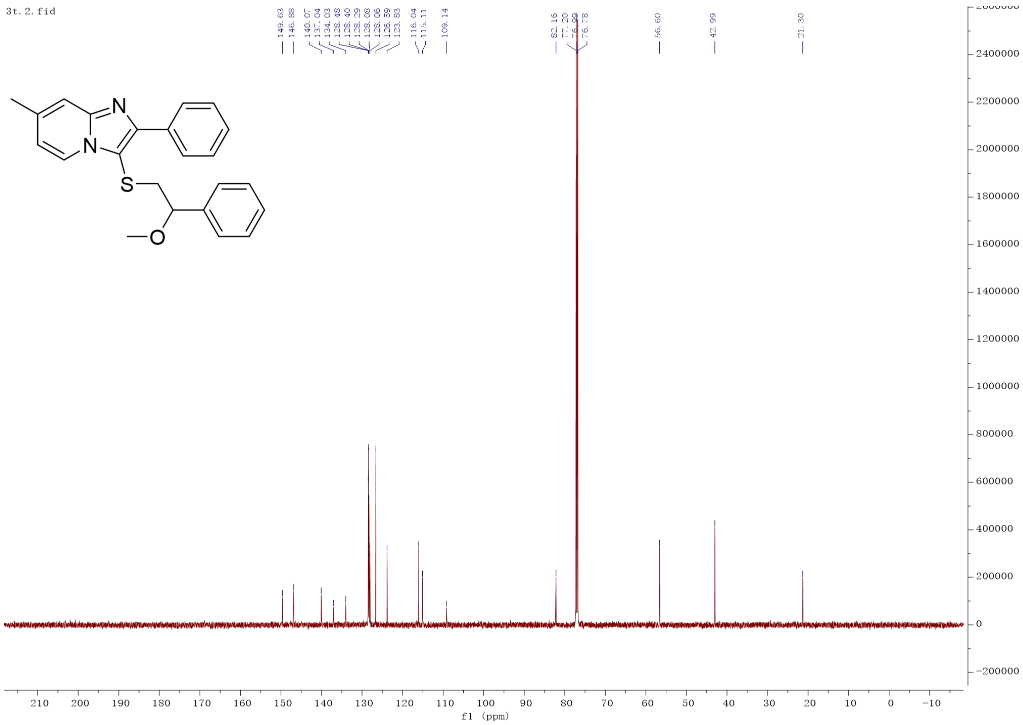

3-((2-methoxy-2-phenyl)ethylthio)-6-methyl-2-phenylimidazo[1,2-*a*]pyridine (3u)

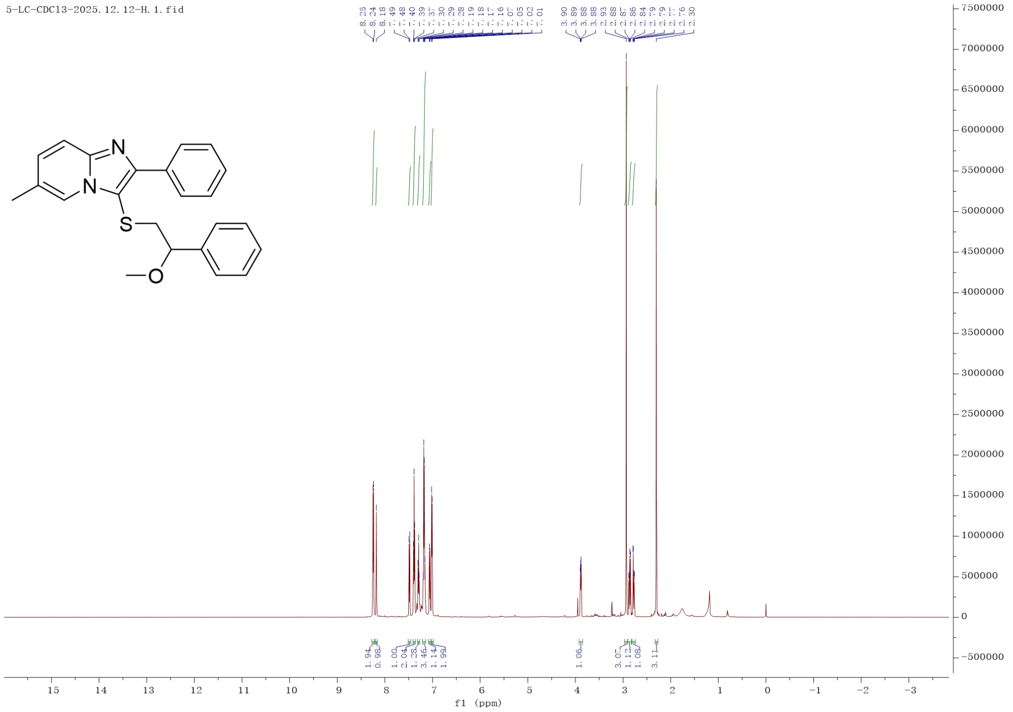

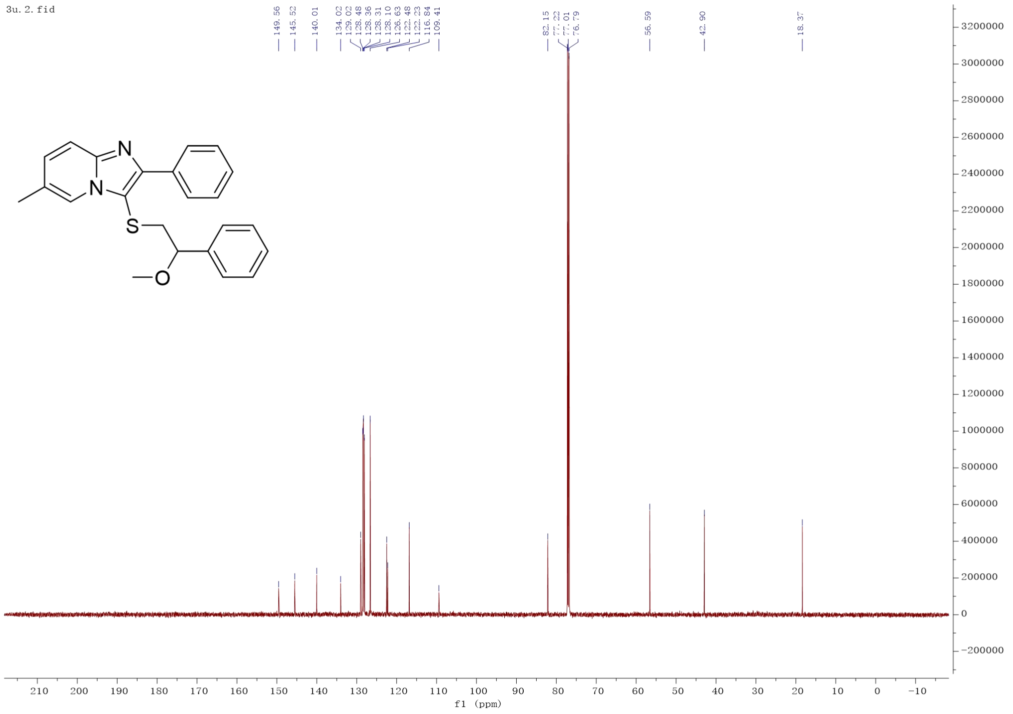

3-((2-methoxy-2-(2-methyl)phenyl)ethylthio)-2-phenylimidazo[1,2-*a*]pyridine (4a)

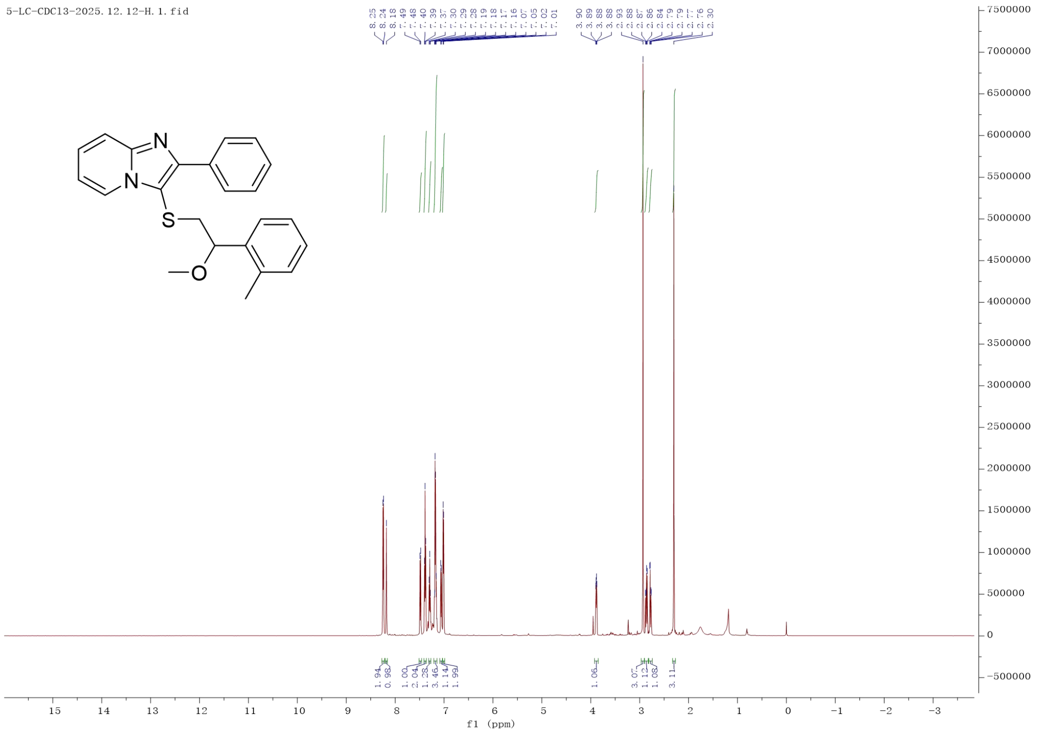

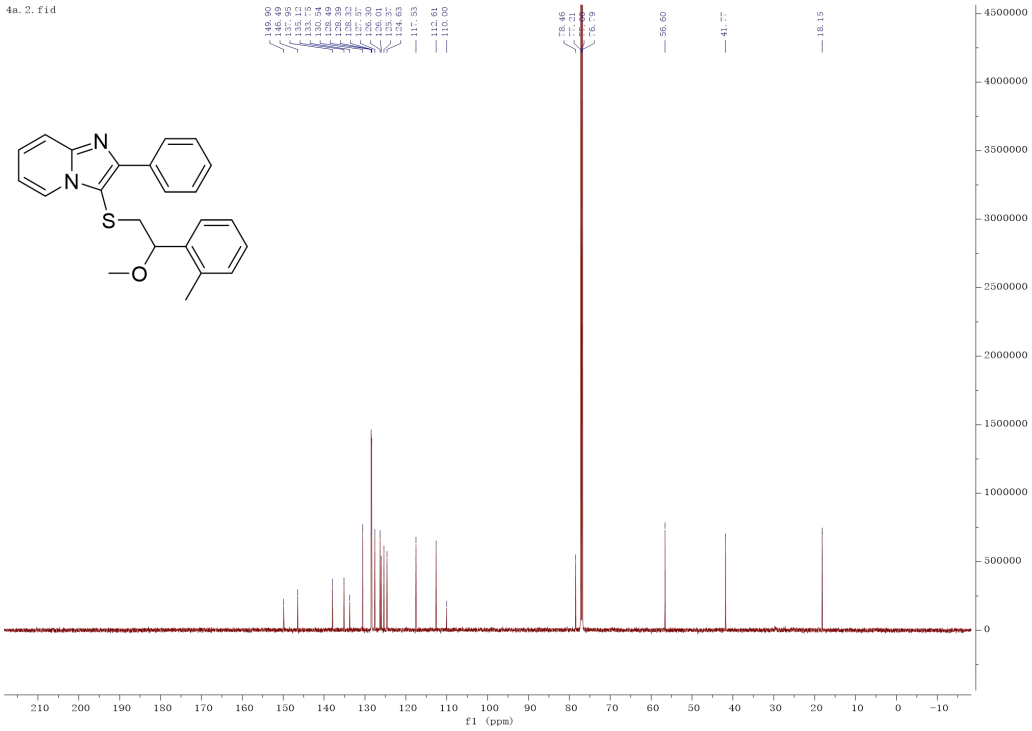

3-((2-ethoxy-2-(2-methyl)phenyl)ethylthio)-2-phenylimidazo[1,2-*a*]pyridine (4b)

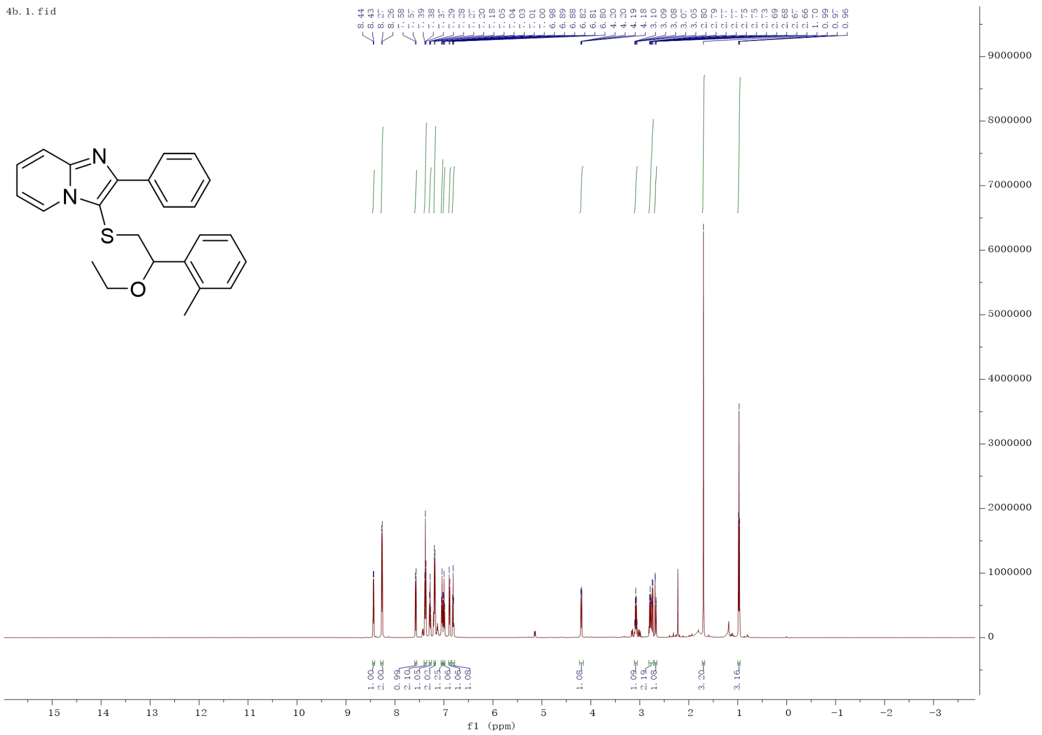

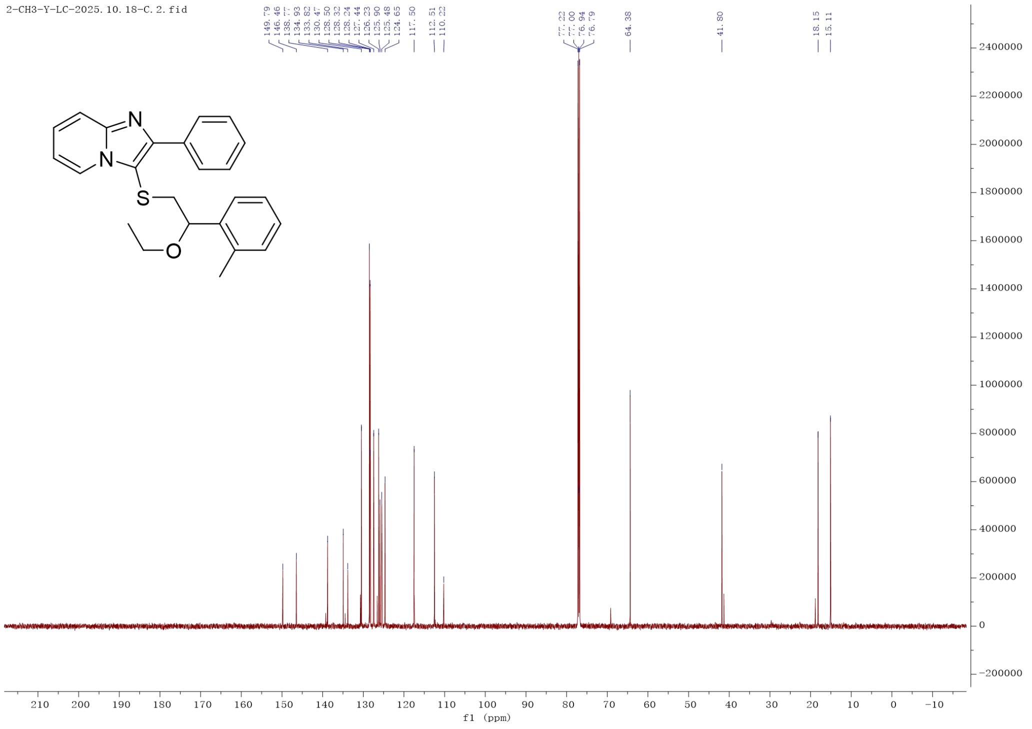

3-((2-methoxy-2-(3-methyl)phenyl)ethylthio)-2-phenylimidazo[1,2-*a*]pyridine (4c)

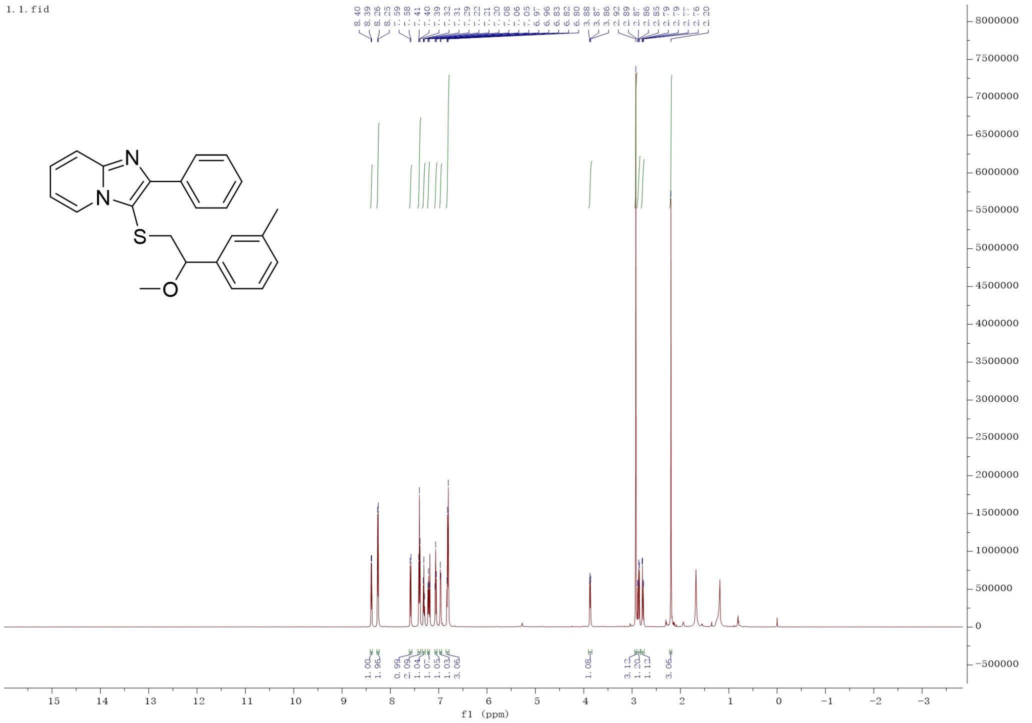

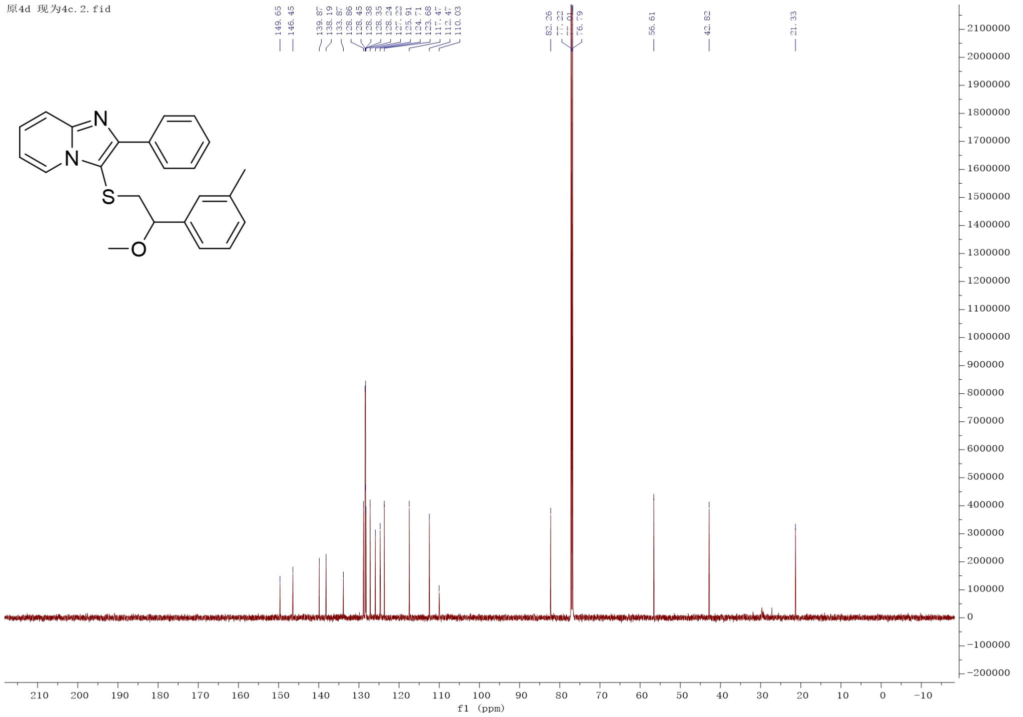

3-((2-ethoxy-2-(3-methyl)phenyl)ethylthio)-2-phenylimidazo[1,2-*a*]pyridine (4d)

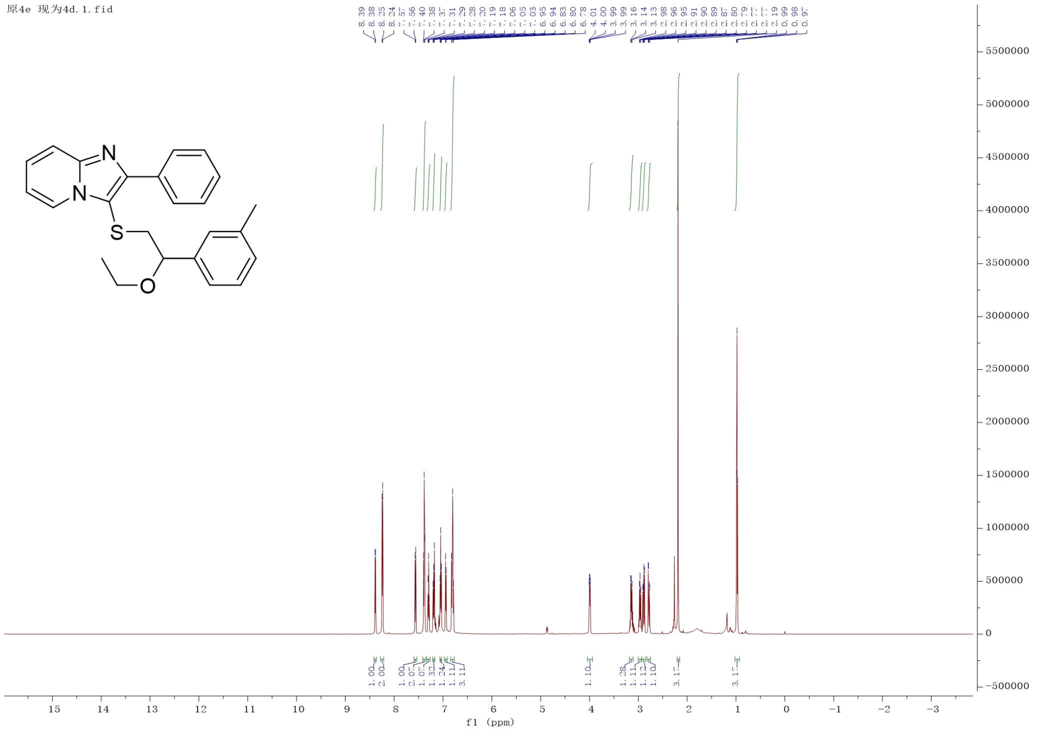

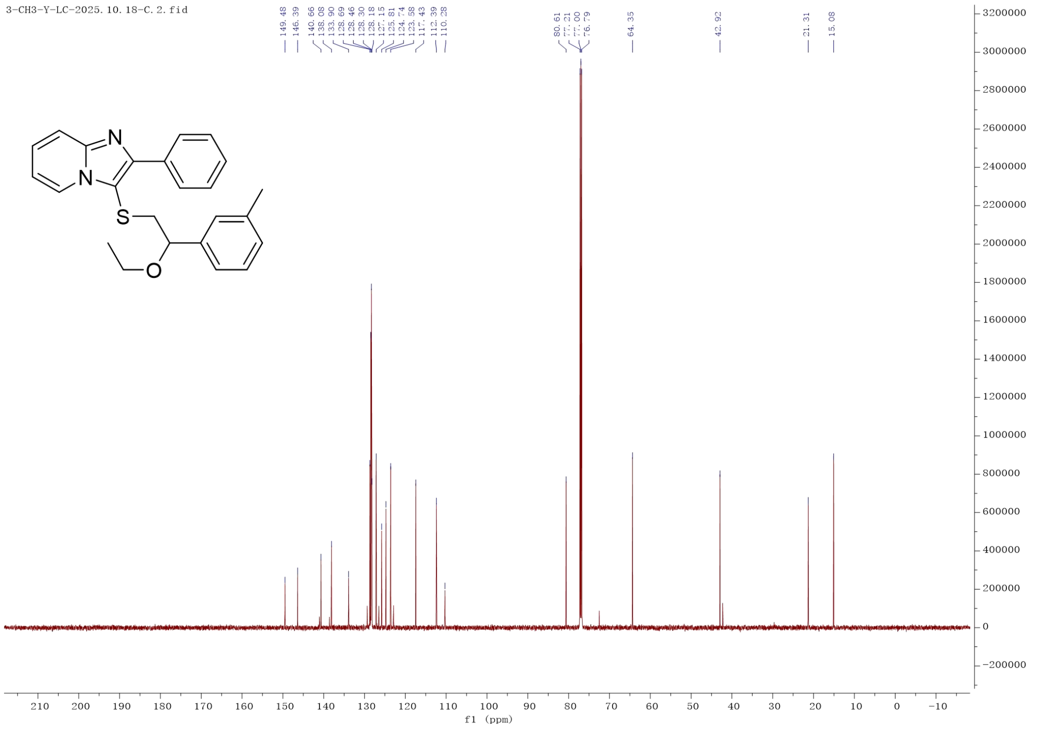

3-((2-methoxy-2-(4-methyl)phenyl)ethylthio)-2-phenylimidazo[1,2-*a*]pyridine (4e)

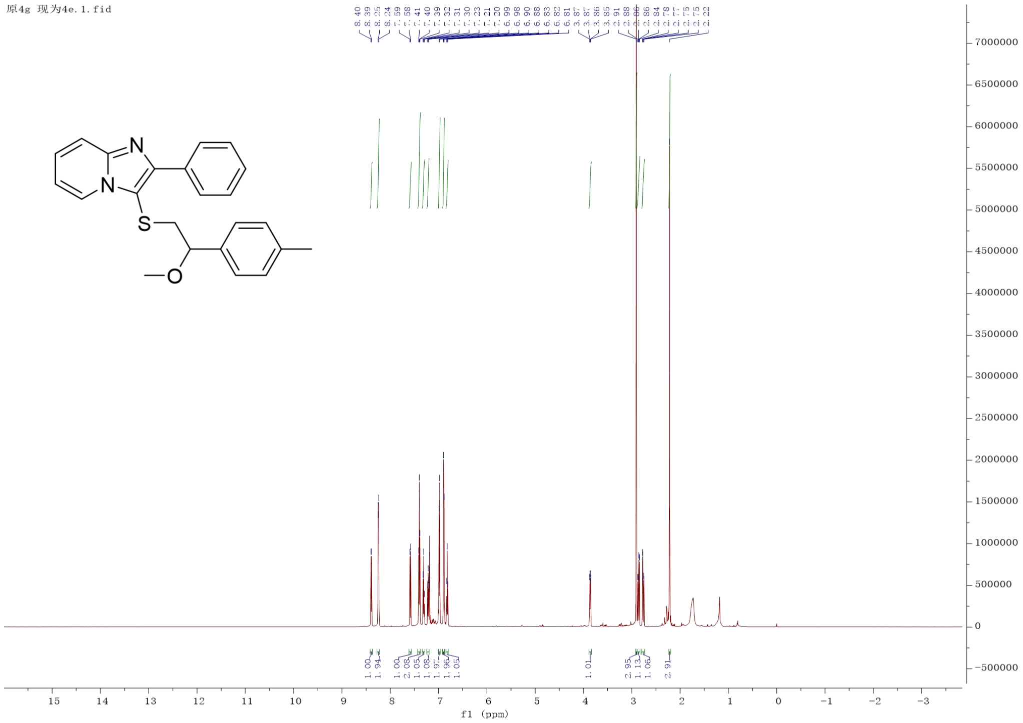

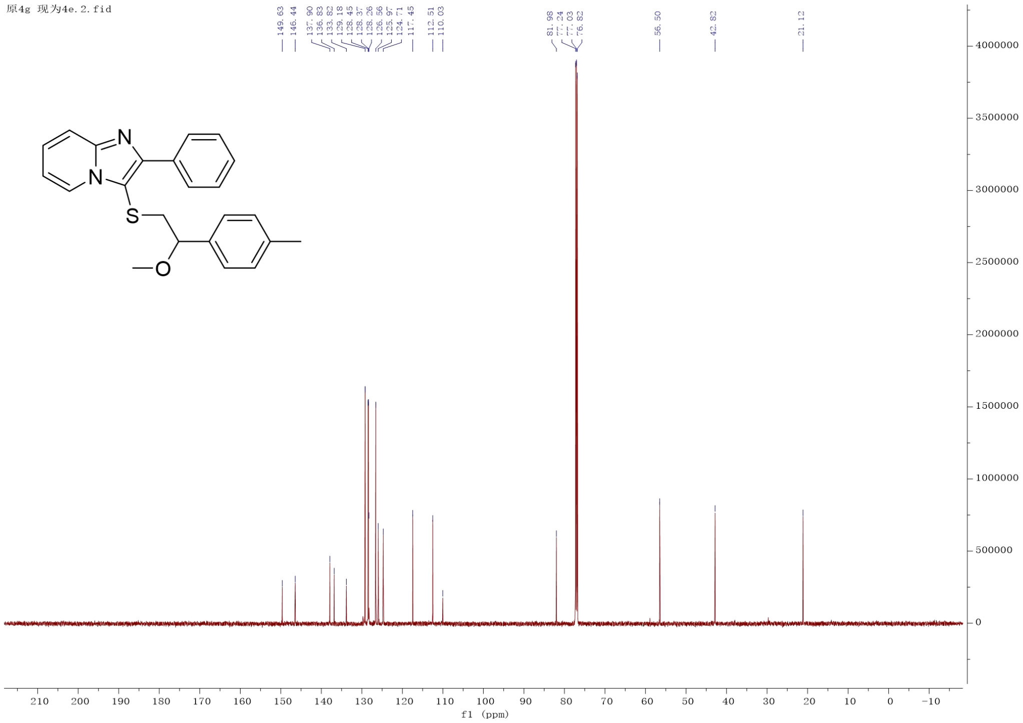

3-((2-ethoxy-2-(4-methyl)phenyl)ethylthio)-2-phenylimidazo[1,2-*a*]pyridine (4f)

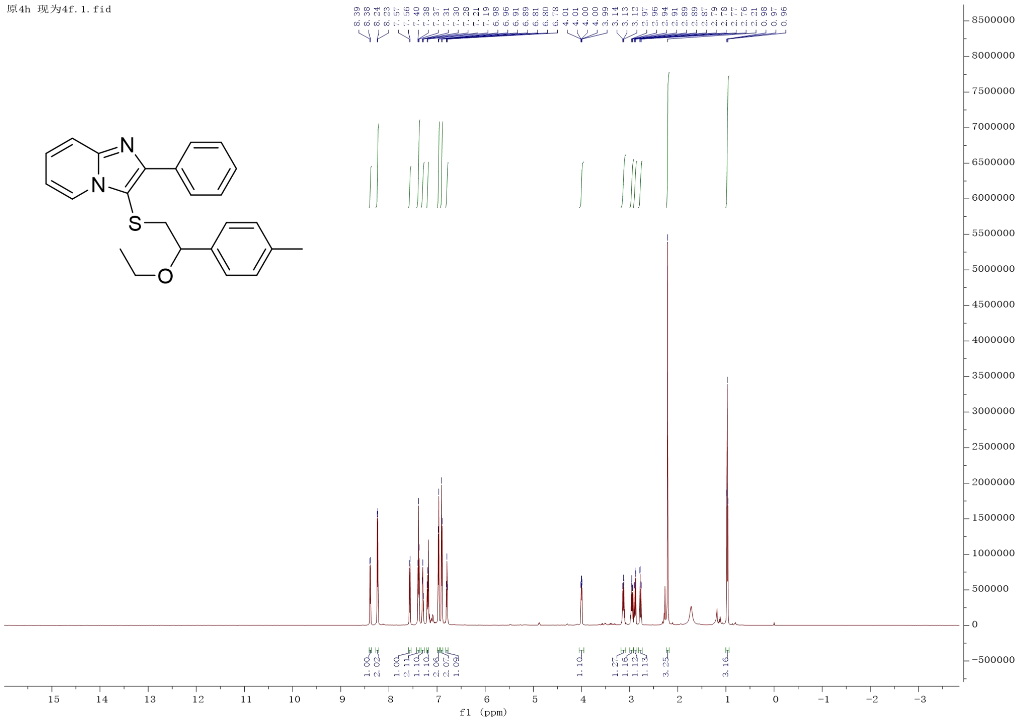

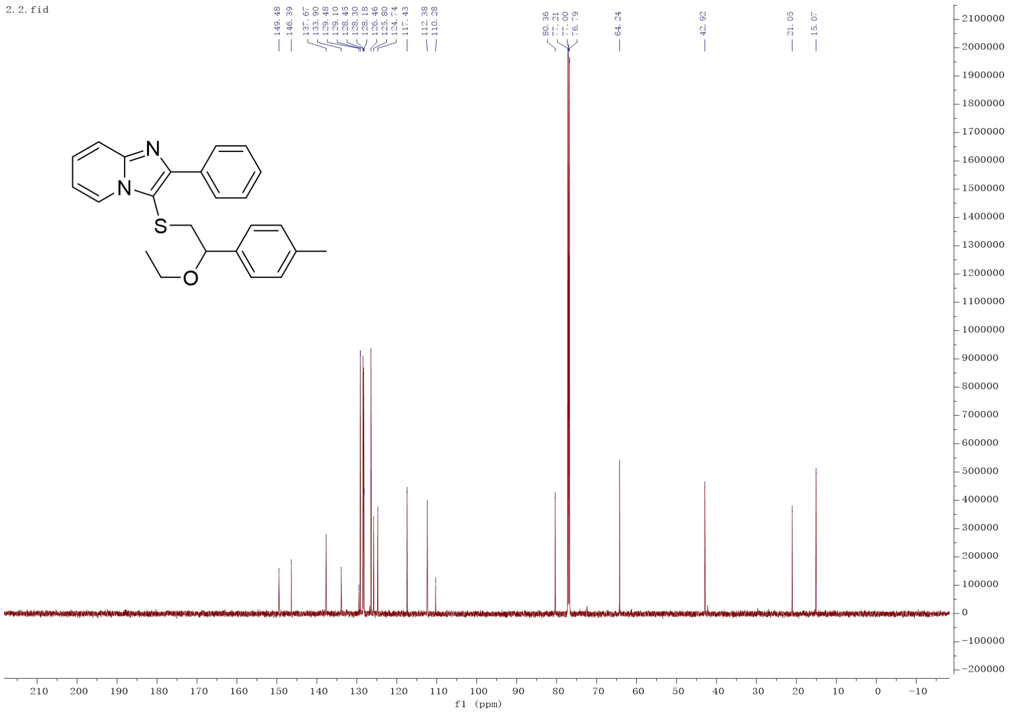

3-((2-(2-chlorophenyl)-2-methoxy)ethylthio)-2-phenylimidazo[1,2-*a*]pyridine (4g)

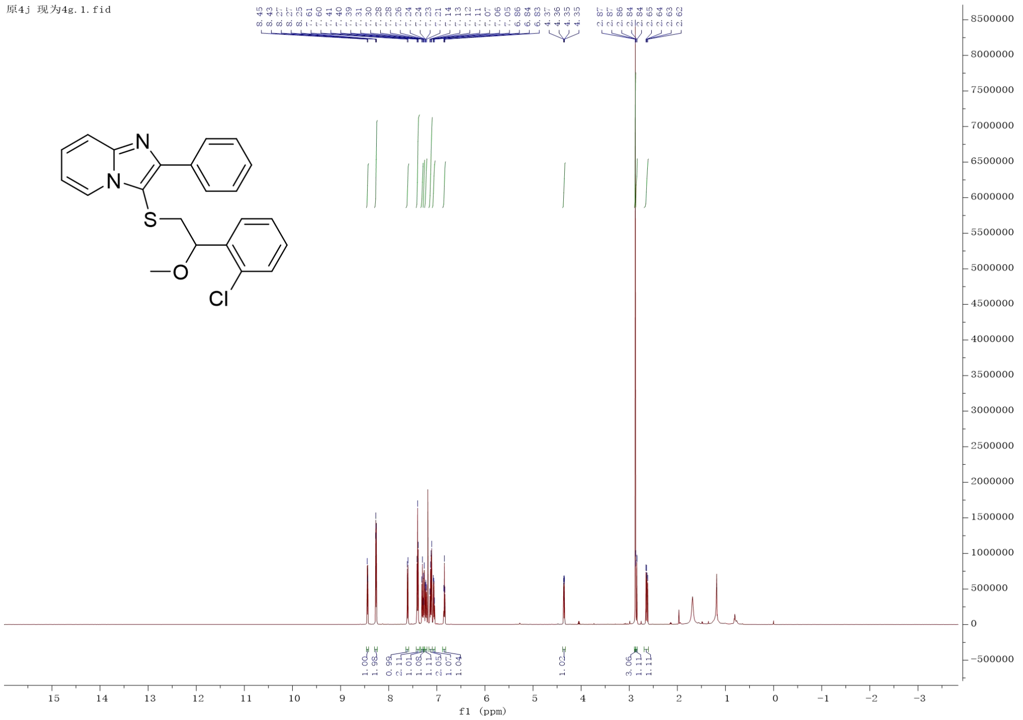

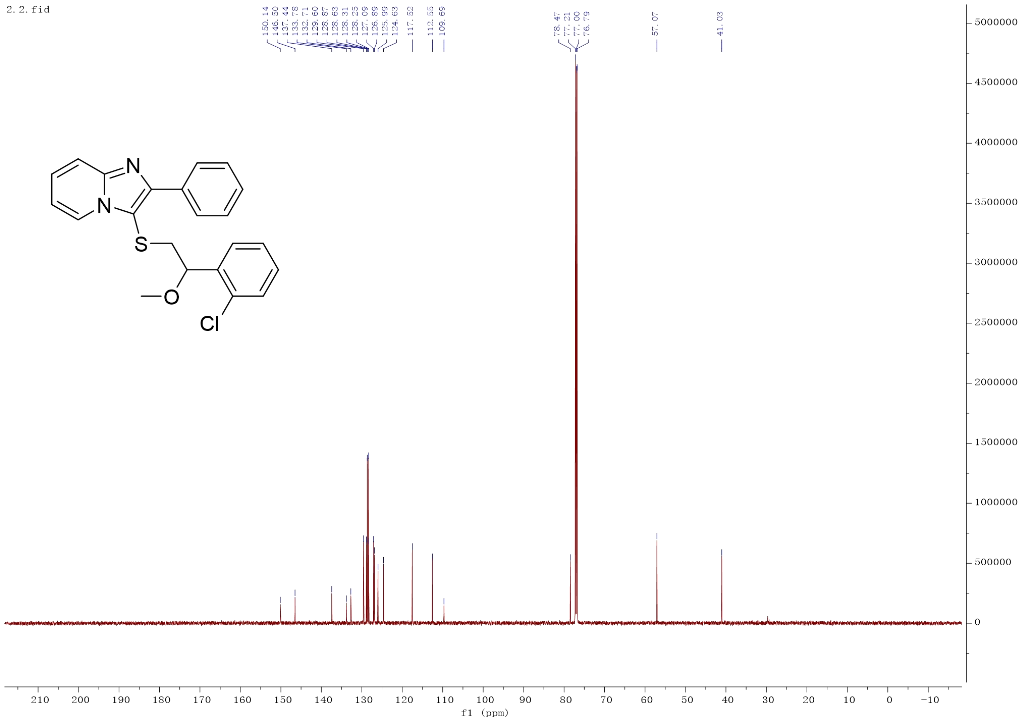

3-((2-(2-chlorophenyl)-2-ethoxy)ethylthio)-2-phenylimidazo[1,2-*a*]pyridine (4h)

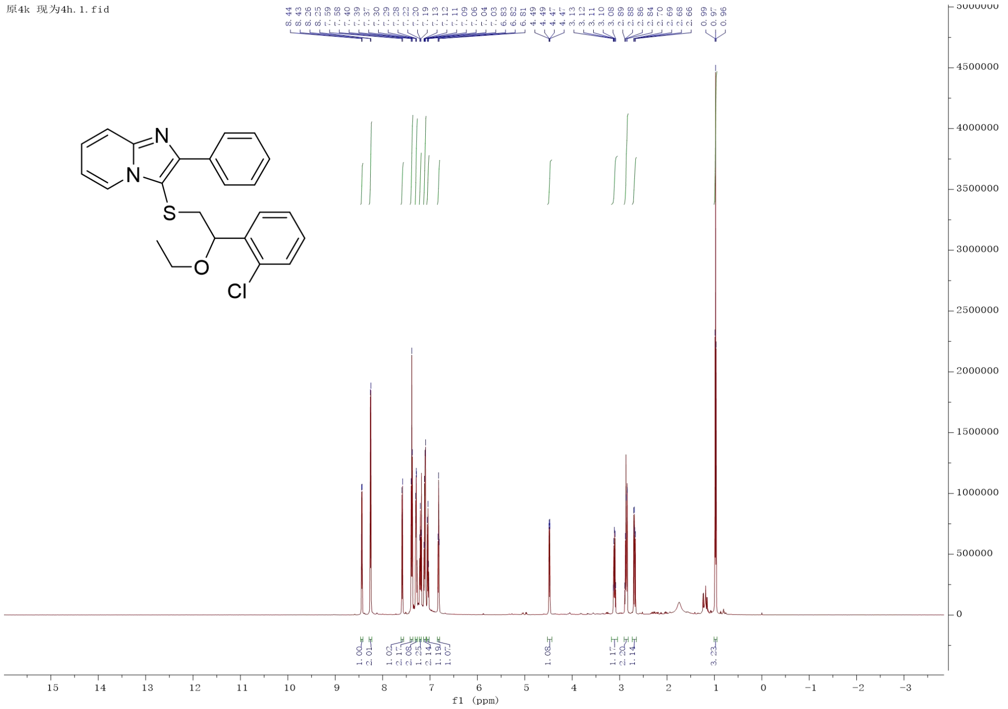

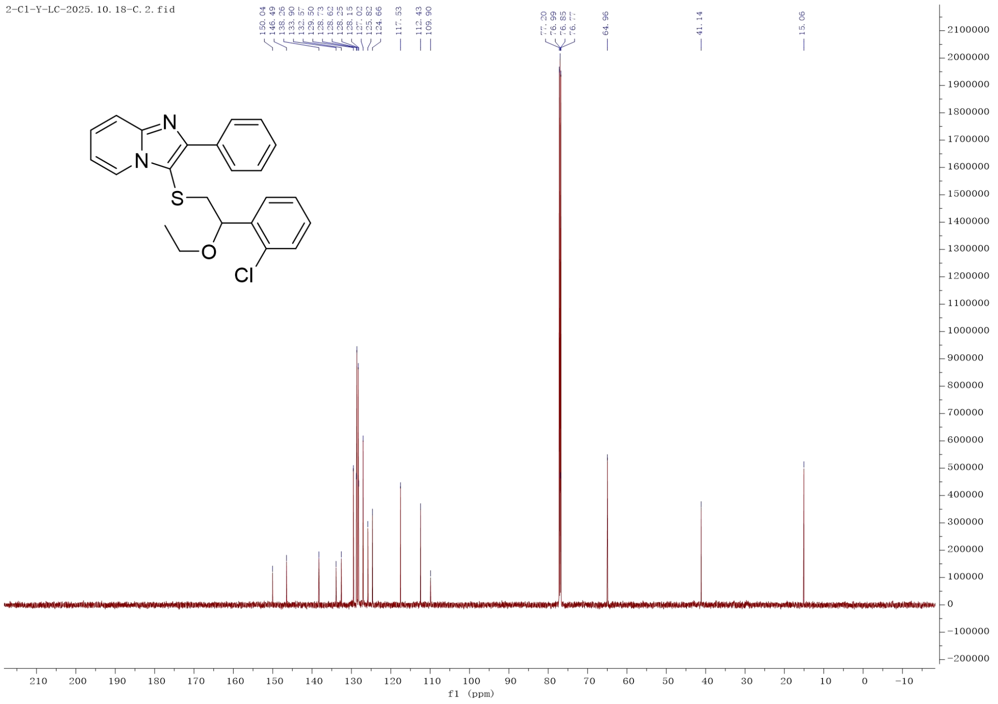

3-((2-(3-chlorophenyl)-2-methoxy)ethylthio)-2-phenylimidazo[1,2-*a*]pyridine (4i)

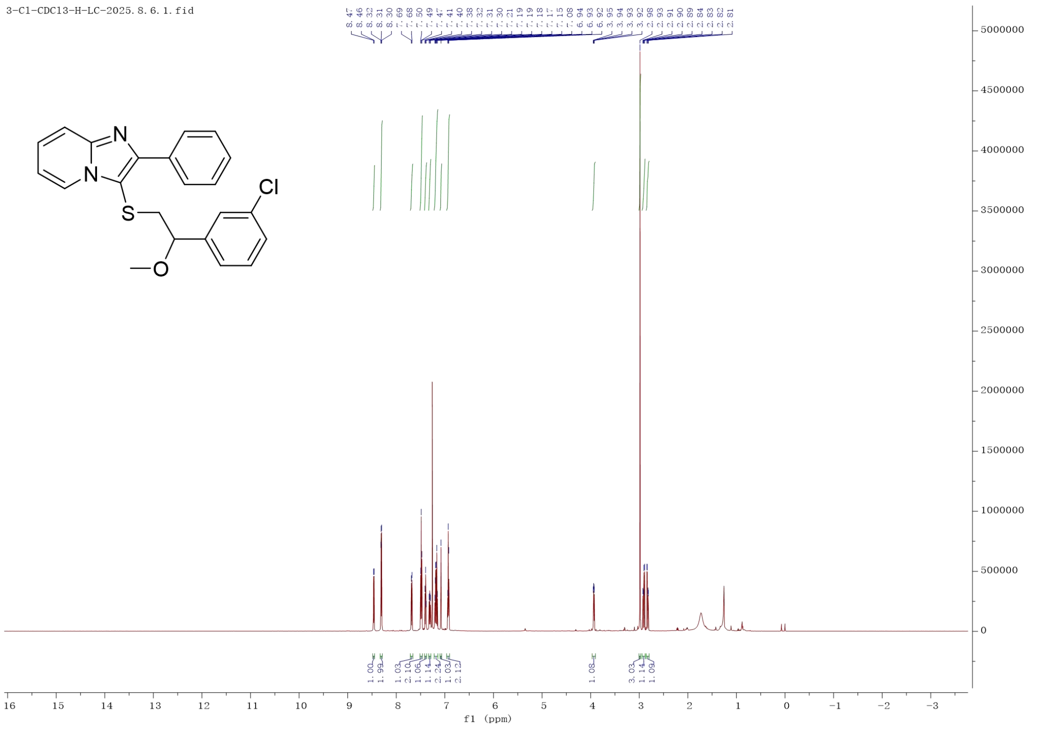

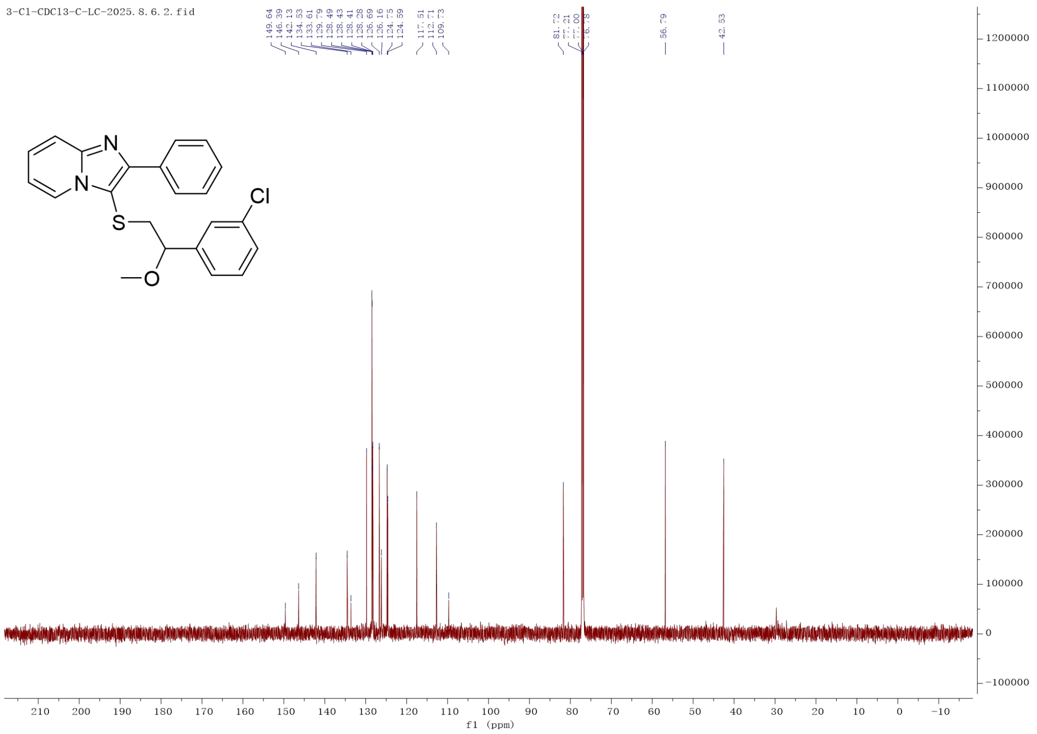

3-((2-(3-chlorophenyl)-2-ethoxy)ethylthio)-2-phenylimidazo[1,2-*a*]pyridine (4j)

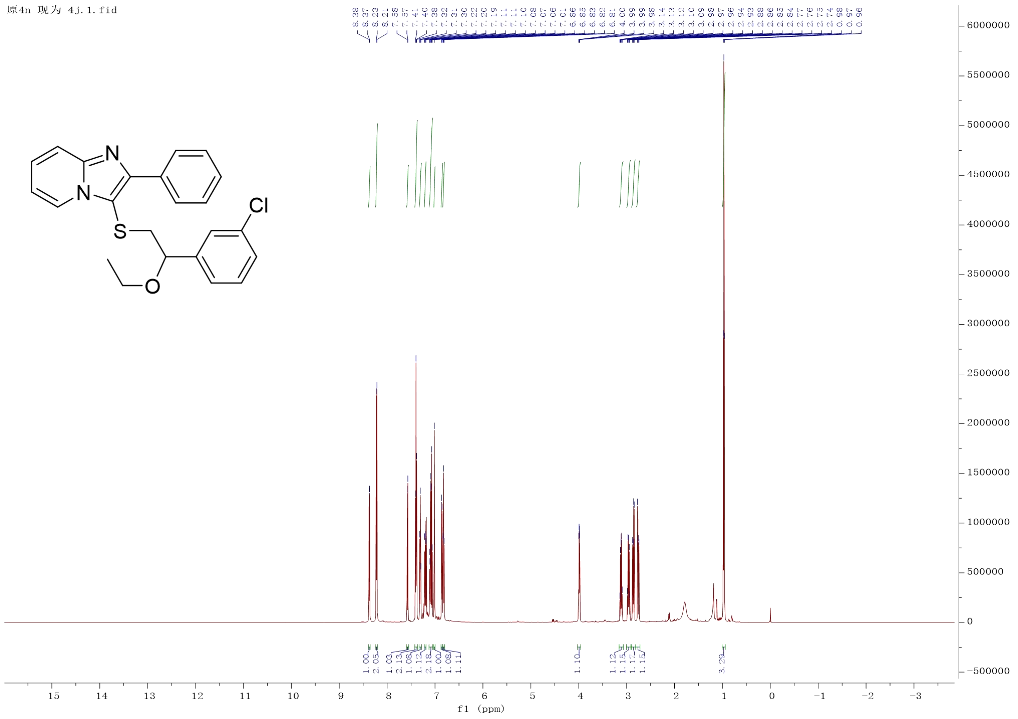

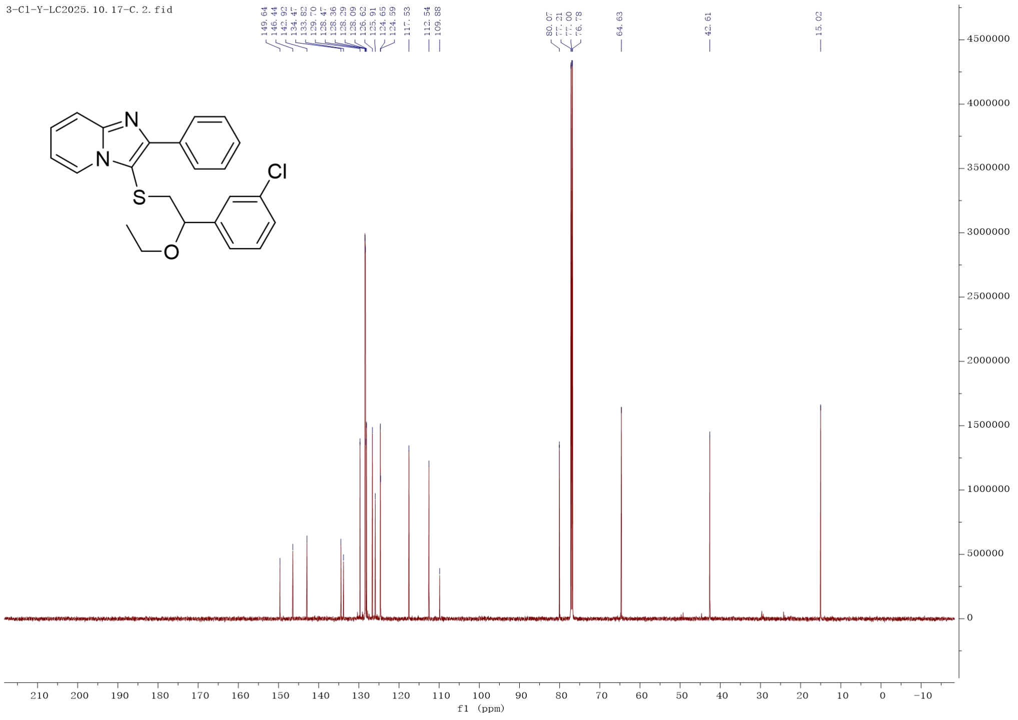

3-((2-(4-chlorophenyl)-2-methoxy)ethylthio)-2-phenylimidazo[1,2-*a*]pyridine (4k)

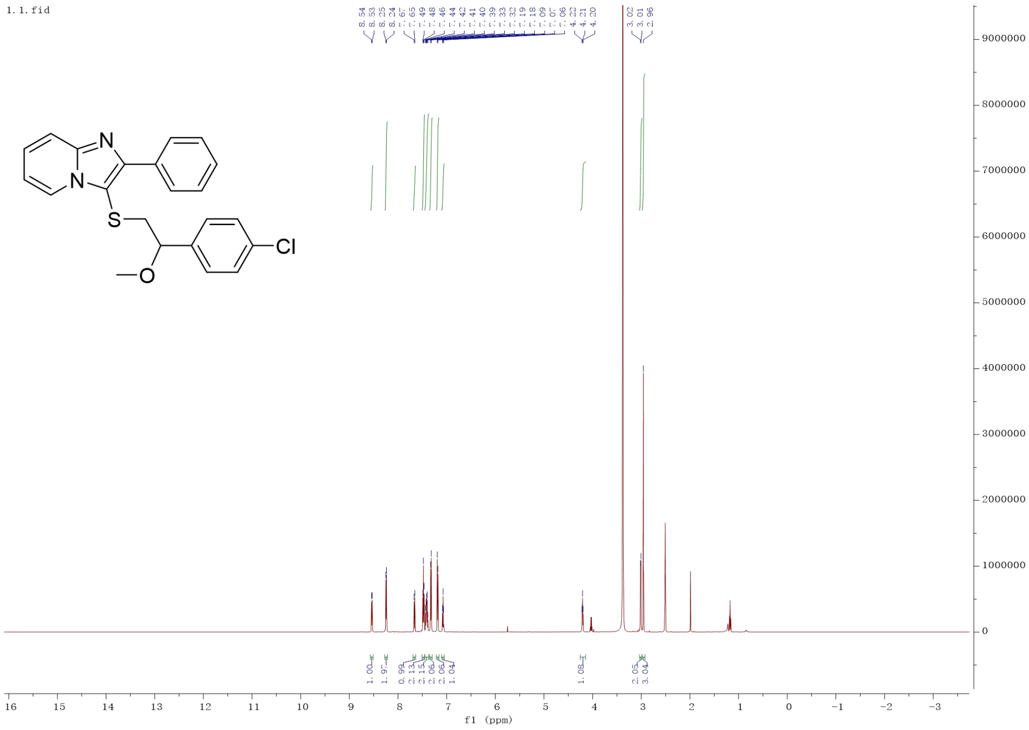

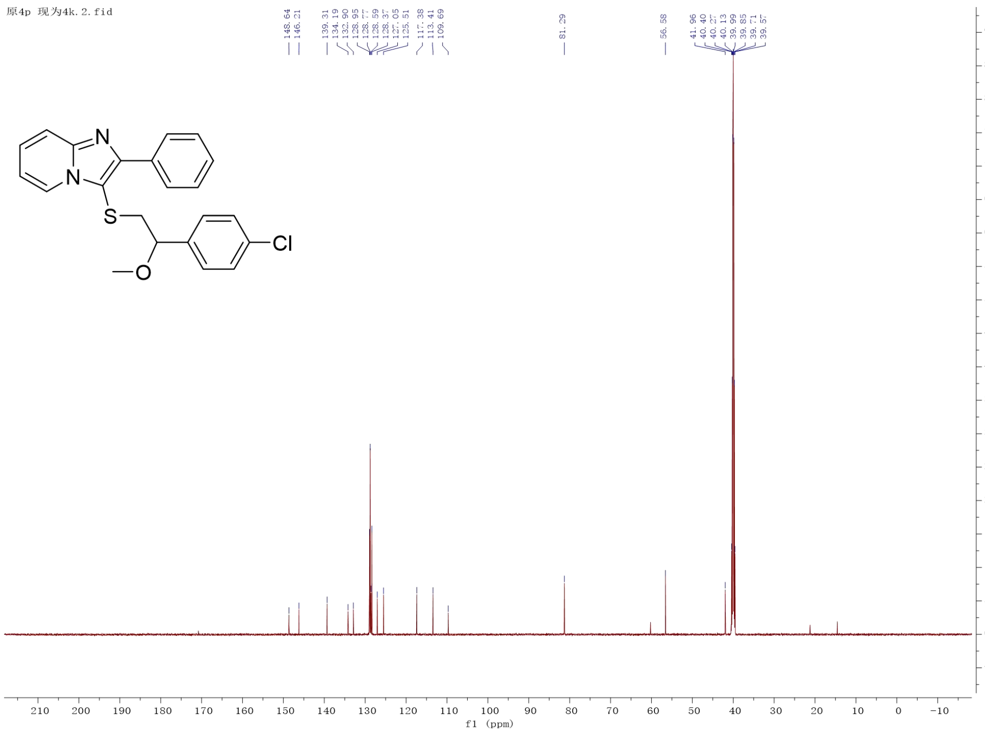

3-((2-(4-chlorophenyl)-2-ethoxy)ethylthio)-2-phenylimidazo[1,2-*a*]pyridine (4l)

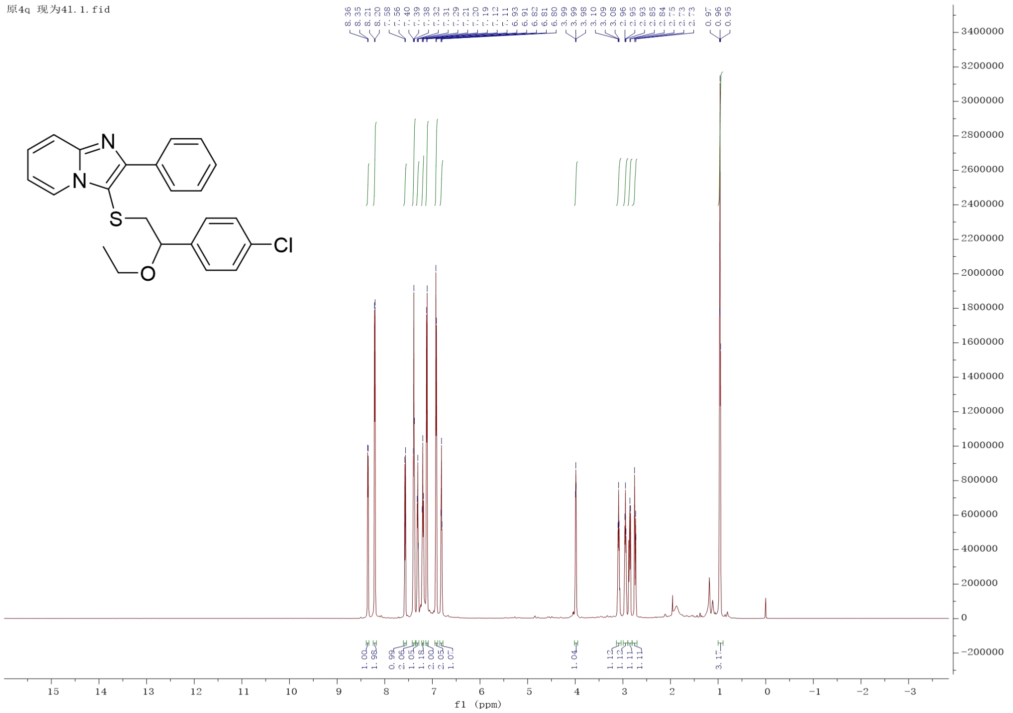

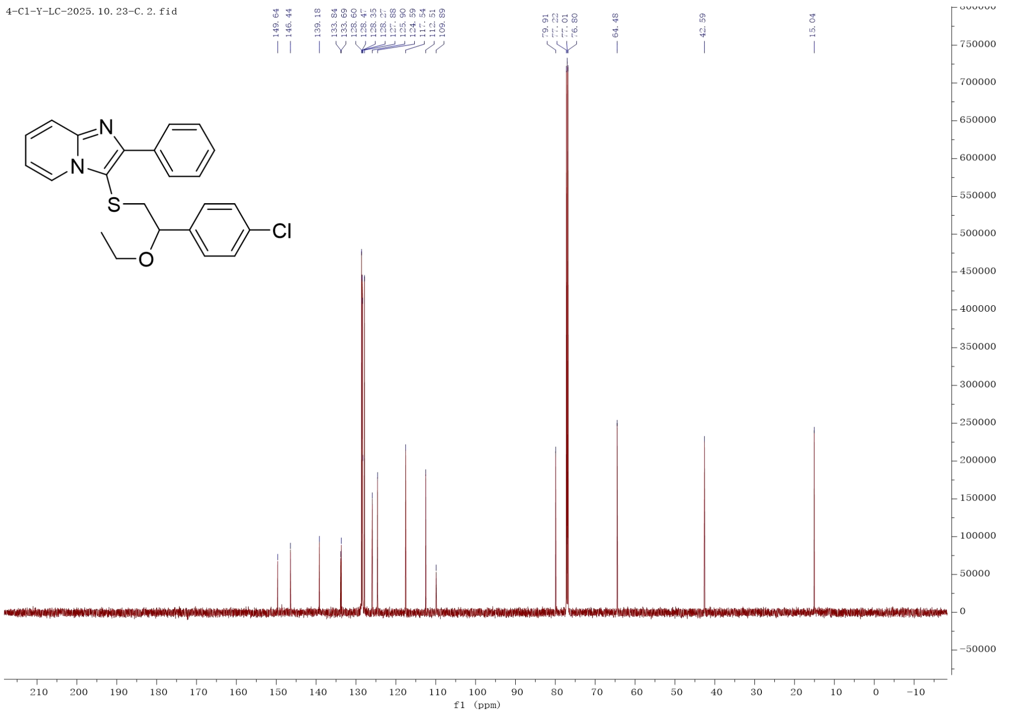

3-((2-(2-bromophenyl)-2-methoxy)ethylthio)-2-phenylimidazo[1,2-*a*]pyridine (4m)

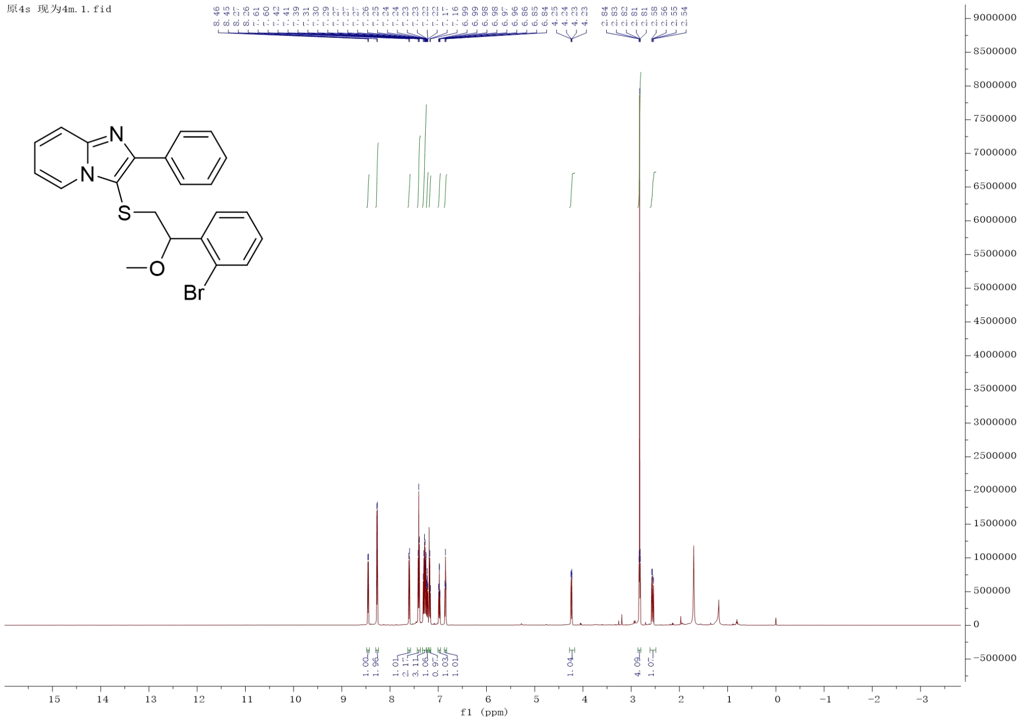

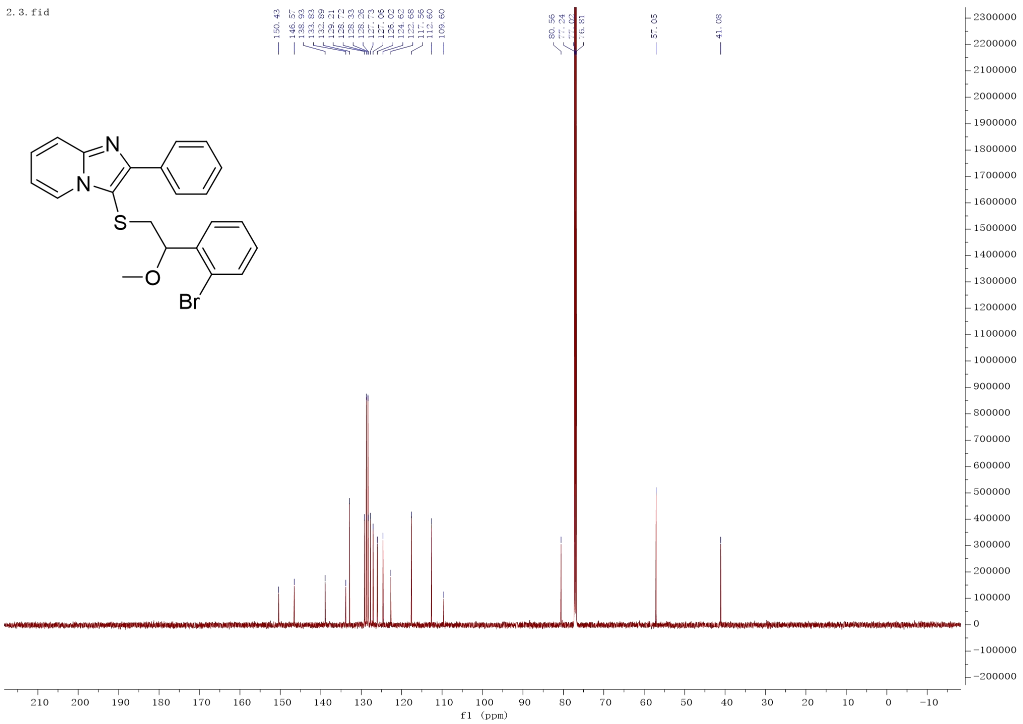

3-((2-(3-bromophenyl)-2-methoxy)ethylthio)-2-phenylimidazo[1,2-*a*]pyridine (4n)

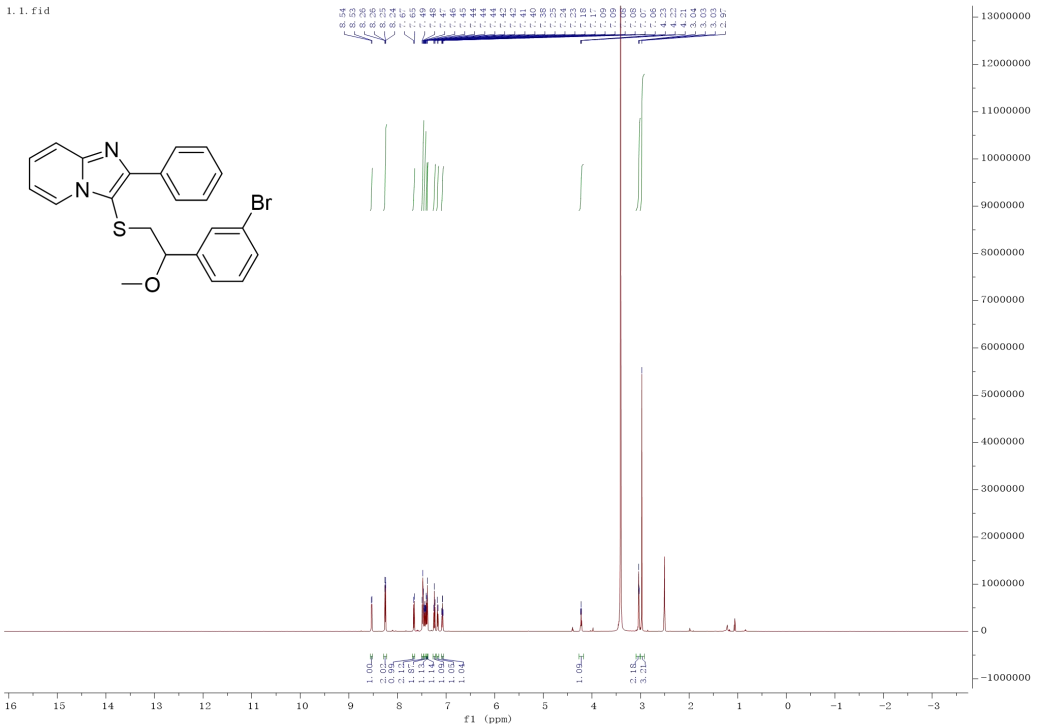

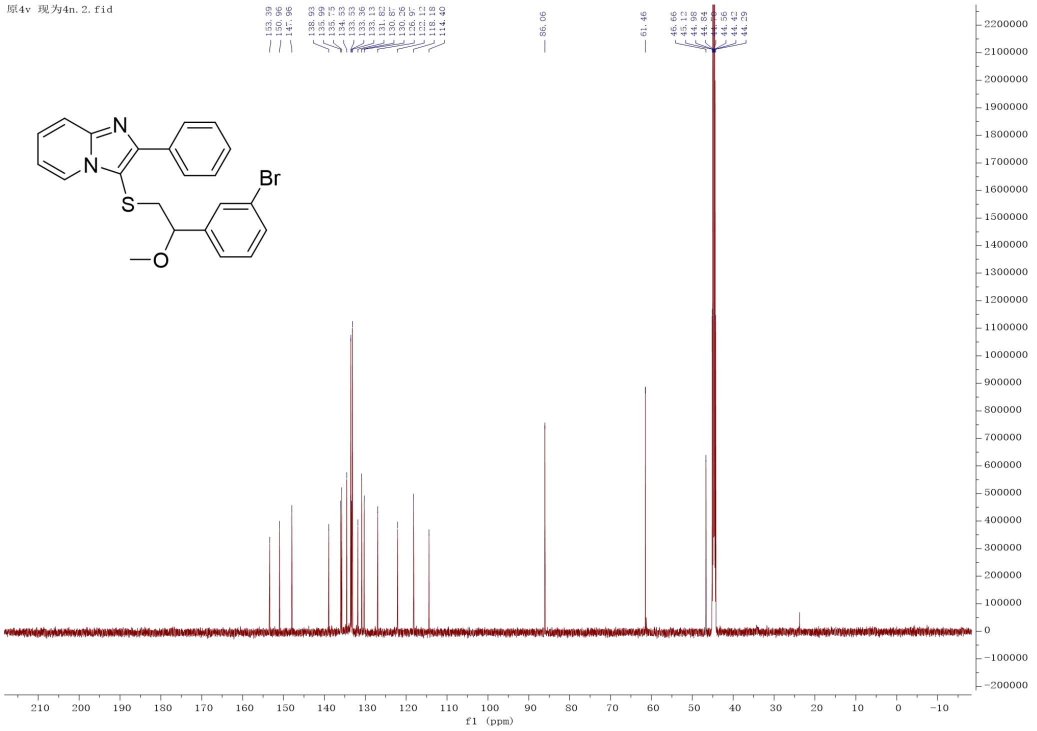

Supplement: RA-016-D6RA02944B-s001 [file RA-016-D6RA02944B-s001.pdf]
